# *Arabidopsis* RETINOBLASTOMA RELATED directly regulates DNA damage responses through functions beyond cell cycle control

**DOI:** 10.15252/embj.201694561

**Published:** 2017-03-20

**Authors:** Beatrix M Horvath, Hana Kourova, Szilvia Nagy, Edit Nemeth, Zoltan Magyar, Csaba Papdi, Zaki Ahmad, Gabino F Sanchez‐Perez, Serena Perilli, Ikram Blilou, Aladár Pettkó‐Szandtner, Zsuzsanna Darula, Tamas Meszaros, Pavla Binarova, Laszlo Bogre, Ben Scheres

**Affiliations:** ^1^ School of Biological SciencesCentre for Systems and Synthetic BiologyRoyal Holloway, University of LondonEghamUK; ^2^ Department of Molecular GeneticsUtrecht UniversityUtrechtThe Netherlands; ^3^ Institute of Microbiology CASv.v.i., Laboratory of Cell ReproductionPrague 4Czech Republic; ^4^ Department of Medical Chemistry, Molecular Biology and PathobiochemistrySemmelweis UniversityBudapestHungary; ^5^ Institute of Plant BiologyBiological Research CentreSzegedHungary; ^6^ Department of Plant SciencesWageningen University Research CentreWageningenThe Netherlands; ^7^ Laboratory of Proteomic ResearchBiological Research CentreSzegedHungary; ^8^ Technical Analytical Research Group of HASBudapestHungary

**Keywords:** *Arabidopsis*, BRCA1, DNA damage response, E2FA, RETINOBLASTOMA RELATED, Cell Cycle, DNA Replication, Repair & Recombination, Plant Biology

## Abstract

The rapidly proliferating cells in plant meristems must be protected from genome damage. Here, we show that the regulatory role of the *Arabidopsis* RETINOBLASTOMA RELATED (RBR) in cell proliferation can be separated from a novel function in safeguarding genome integrity. Upon DNA damage, RBR and its binding partner E2FA are recruited to heterochromatic γH2AX‐labelled DNA damage foci in an ATM‐ and ATR‐dependent manner. These γH2AX‐labelled DNA lesions are more dispersedly occupied by the conserved repair protein, AtBRCA1, which can also co‐localise with RBR foci. RBR and AtBRCA1 physically interact *in vitro* and *in planta*. Genetic interaction between the RBR‐silenced *amiRBR* and *Atbrca1* mutants suggests that RBR and AtBRCA1 may function together in maintaining genome integrity. Together with E2FA, RBR is directly involved in the transcriptional DNA damage response as well as in the cell death pathway that is independent of SOG1, the plant functional analogue of p53. Thus, plant homologs and analogues of major mammalian tumour suppressor proteins form a regulatory network that coordinates cell proliferation with cell and genome integrity.

## Introduction

The continuous post‐embryonic growth of plants is supported by rapidly proliferating cells in meristems. Protection against the accumulation of mutations in dividing cells is not only important to maintain cellular functions, but additionally to maintain the source for generative cells throughout plant life (Scheres, 2007; Hu *et al*, 2016). The *Arabidopsis* RETINOBLASTOMA RELATED (RBR) is a conserved regulator of cell proliferation, differentiation, and stem cell niche maintenance (Harashima & Sugimoto, 2016). RBR regulates cell proliferation by restraining E2F‐dependent transcription of cell cycle genes (Magyar *et al*, 2005; Gutzat *et al*, 2012; Kobayashi *et al*, 2015; Harashima & Sugimoto, 2016). Mitogenic signals promote RBR phosphorylation by cyclin‐dependent kinases (CDKs) in association with D‐type cyclins, the best characterised being CYCLIN D3;1 (CYCD3;1) (Dewitte *et al*, 2003; Magyar *et al*, 2012). Upon this RBR phosphorylation, the E2FB transcription factor is released and promotes cell cycle gene expression and cell proliferation, while E2FA remains associated with RBR and maintains meristems through repression of differentiation (Harashima *et al*, 2013; Kuwabara & Gruissem, 2014; Magyar *et al*, 2012; Polyn *et al*, 2015). The developmental role of RBR is best understood in the root meristem, where slowly dividing quiescent centre (QC) cells maintain surrounding root stem cells that divide more frequently. The low rate of cell division in the QC protects cells against DNA damage while surrounding stem cells are more sensitive (Fulcher & Sablowski, 2009; Furukawa *et al*, 2010). RBR, in complex with the transcription factor SCARECROW, was shown to regulate specific stem cell divisions but also impose quiescence, which is important to protect against replication stress‐induced cell death (Cruz‐Ramirez *et al*, 2012, 2013). RBR is also required during meiosis for chromosome condensation and synapsis of homologous chromosomes, but not for introducing DSBs for homologous recombination (Chen *et al*, 2011).

DNA damaging environmental factors, such as ionising radiation, ultraviolet light, excess of metalloid elements (Br, Al) and internal damage generated spontaneously during DNA metabolism, can all impact on genome integrity (Hoeijmakers, 2009). To counteract the consequences of DNA lesions, organisms have evolved DNA damage response pathways (DDR). The recognition of DNA damage by sensor proteins initiates a network of molecular events that recruit the DNA repair machinery, regulate transcription, control cell cycle progression, eliminate damaged cells by cell death and enter into terminal differentiation or senescence (Su, 2006; Cools & De Veylder, 2009; Ciccia & Elledge, 2010; Sherman *et al*, 2011; Hu *et al*, 2016).

Depending on whether DNA damage results in exposed single‐strand (SS) or double‐strand breaks (DSBs), different signalling pathways are induced, involving alternative sets of sensors, mediators and effectors (Ciccia & Elledge, 2010). The central components are largely conserved among yeasts, animals and plants, although kingdom‐specific proteins are also involved (Harper & Elledge, 2007; Waterworth *et al*, 2011; Amiard *et al*, 2013; Yoshiyama *et al*, 2013b). The conserved DNA damage sensing kinase ATAXIA‐TELANGIECTASIA MUTATED (ATM) is activated by double‐strand DNA breaks (DSBs) and acts during G1/S and G2/M checkpoints; its role recently was also implicated in the regulation of oxidative stress (Shiloh & Ziv, 2013; Shiloh, 2014). The ATAXIA‐TELANGIECTASIA‐AND‐RAD3 RELATED (ATR) mainly responds to free single‐stranded DNA, formed during processing of blocked replication forks, at G1/S and intra‐S checkpoints (Culligan *et al*, 2006; Cimprich & Cortez, 2008; Culligan & Britt, 2008; Flynn & Zou, 2011; Amiard *et al*, 2013).

In mammalian systems, the ATM kinase phosphorylates the histone variant H2AX (γH2AX) upon activation by DSB and initiates a cascade of events through recruiting numerous signalling proteins and DNA repair proteins, such as the breast and ovarian cancer type1 susceptibility protein, BRCA1. Single‐stranded DNA, the signal for replication stress, is sensed and bound by the mammalian replication protein A (RPA) to form a complex. The resulting complex activates ATR leading to the phosphorylation of the tumour suppressor protein, p53 and delay of S‐phase, allowing the recovery of collapsed replication forks (Ciccia & Elledge, 2010).

No direct homologs of p53 have been identified in plants (Yoshiyama *et al*, 2013b), but the plant‐specific transcription factor, SUPPRESSOR OF GAMMA RESPONSE 1 (SOG1) is considered to be a functional analogue of p53 (Cimprich & Cortez, 2008; Yoshiyama *et al*, 2013b). SOG1 is directly phosphorylated and activated by ATM. Active SOG1 induces transcription of genes related to DNA damage response and genes that impose cell cycle checkpoint or repair (Culligan *et al*, 2006; Ricaud *et al*, 2007; Yoshiyama *et al*, 2009). Upon DNA damage, ATM and ATR activate the WEE1 kinase, which mainly controls the replication checkpoint (De Schutter *et al*, 2007; Dissmeyer *et al*, 2009; Cools *et al*, 2011). The G2/M DNA damage checkpoint is controlled by the CDKA;1 inhibitors, SIAMESE RELATED 5 and 7, direct targets of phosphorylated SOG1 upon DNA damage (Yi *et al*, 2014).

Here we show that RBR, besides its well‐known function during cell cycle, maintains genome integrity in root meristematic cells. During DNA damage response, RBR together with E2FA accumulates at distinct heterochromatic foci labelled by γH2AX in an ATM/ATR‐dependent manner. AtBRCA1 is generally recruited to numerous γH2AX‐labelled foci upon damage, but less frequently it also co‐localises with RBR. Co‐immunoprecipitation and bimolecular fluorescence complementation (BiFC) studies show that these two proteins can interact and genetic data support that they act together in protecting the genome. In addition, RBR/E2FA acts as a transcriptional repressor of *AtBRCA1* transcription in parallel to the SOG1‐governed transcription of DDR genes.

## Results

### The role of RETINOBLASTOMA RELATED in mediating maintenance of genome integrity is separable from its function in cell cycle regulation

**Figure 1:**
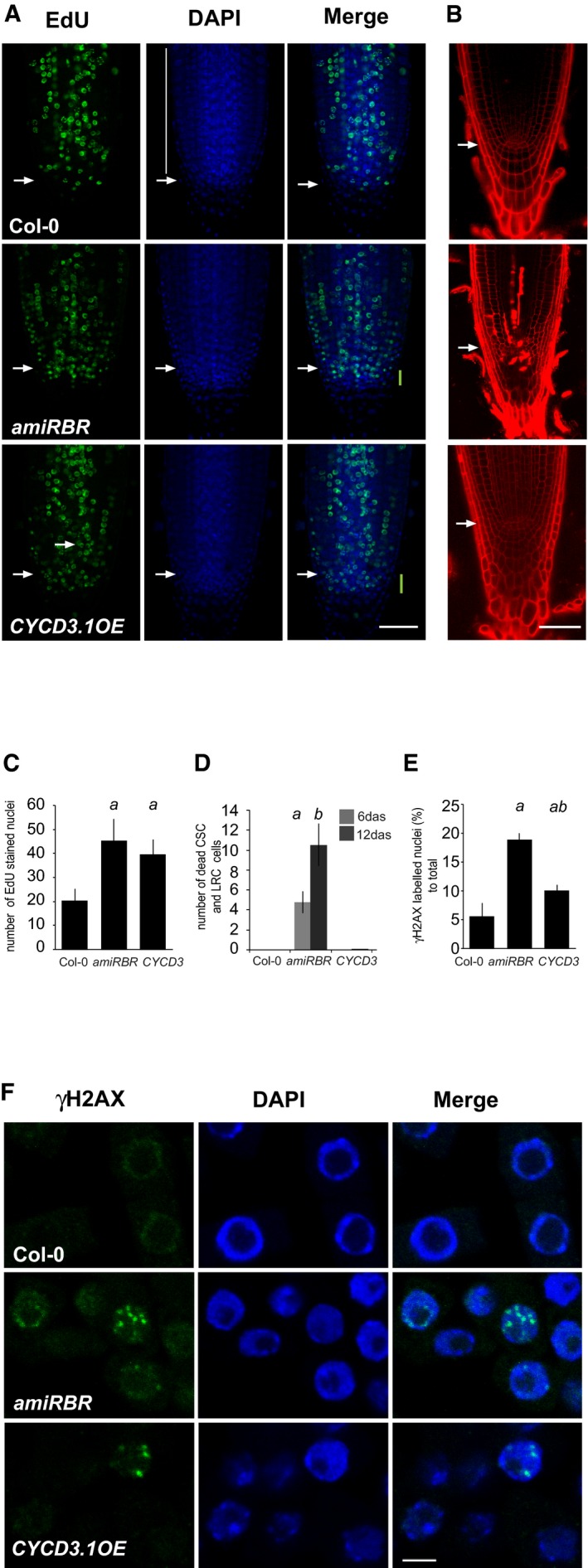
Silencing of RBR and *CYCD3.1* overexpression both promote S‐phase entry but affect cell death response and DNA damage accumulation differently Representative confocal laser scanning microscopy (CM) images of whole mount EdU‐labelled roots from 6‐day‐old (das) seedlings of Col‐0, *amiRBR* and Col‐0(*CYCD3.1OE*) lines with EdU (green) and DAPI (DNA, blue) staining. In the *amiRBR* and Col‐0(*CYCD3.1OE*) lines, the region of extra columella stem cell layers is labelled with a green bar in merged images. White vertical bar shows the region of cells where EdU counting was carried out. Images were taken in single median section, scale bar: 50 μm, arrow: QC position in each image.CM images of propidium iodide (PI)‐stained root tips from 12 das seedling; genotypes indicated as in (A). Images were taken in single median section, scale bar: 50 μm, arrow: QC position in each image.Number of EdU‐labelled cells as shown in (A) was counted in the epidermis, cortex and endodermis cell layers on both sides of the root. In each case, 10 roots (6 das) were quantified.Cell death response in 6 and 12 das seedlings, total number of dead columella stem cells (CSC) and lateral root cap initials (LRC) and their descendants were counted in median sections as shown in (B), *n > *2, *N > *15. Note that in Col‐0(*CYCD3.1OE*) only 1–2 dead cells were detected in the analysed population. Quantification of the dead cell area in *amiRBR* is shown in Fig 2C.Frequency of γH2AX‐labelled nuclei per total number of DAPI‐positive nuclei (%), *n* = 2, *N > *6 root of 6 das seedlings, analysed nuclei > 1,000.Representative CM images (single section) of γH2AX immune‐labelled cells of root tips from Col‐0, *amiRBR* and Col‐0(*CYCD3.1OE*). DAPI (blue), scale bar: 5 μm.Data information: Values represent means with standard deviation (SD). In (C–E), *a* indicates significant difference around 1% confidence using Student's *t‐*test comparing *amiRBR* and Col‐0(*CYCD3.1OE*) to Col‐0. In (D), *b* indicates 99% significance (*P* < 0.01) between time points and in (E) *ab* indicates 99% significance (*P* < 0.01) to Col‐0 and *amiRBR*. *n *= biological repeat, *N *= sample per biological repeat.

**Figure EV1:**
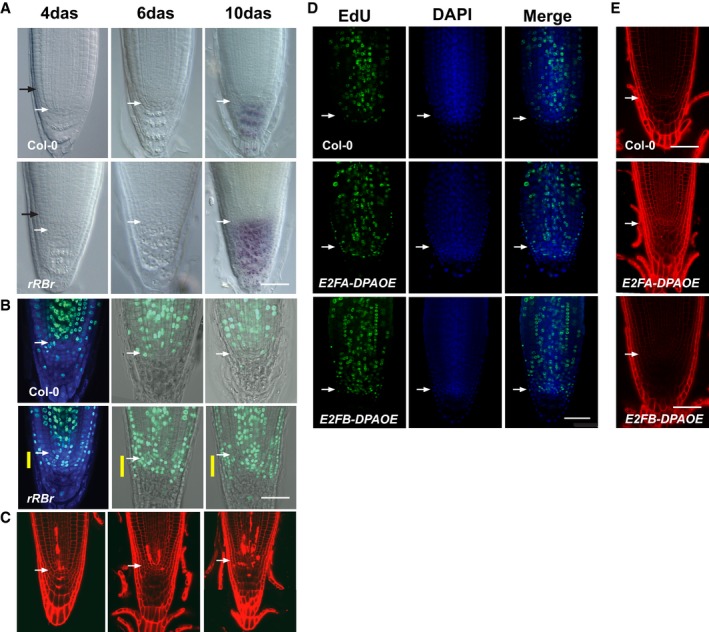
Both local silencing of RBR and overexpression of E2FA‐DPA and E2FB‐DPA result in extra S‐phase entry but only RBR silencing triggers cell death response Differential interference contrast (DIC) microscopy images using Lugol staining to detect differentiated columella cells. Note the increased number of columella cell layers upon local reduction of RBR. Black arrow indicates the position of the dissection used to collect material for micro‐array analysis.Confocal microscopy images (CM) of root tips after EdU staining (green, 2 h) at 4 das counterstained with DAPI (blue) and at 6 and 10 das using bright field. Yellow bar indicates the region with extra columella stem cell layers.CM images of PI‐stained root samples from *rRBr* seedlings showing accumulation of cell death in time.Representative CM images of whole mount EdU‐labelled (green) root tips of 6 das Col‐0, Col‐0(*E2FA/DPAOE*) and Col‐0(*E2FB/DPAOE*) seedlings; DNA was stained by DAPI.Representative PI‐stained CM images of 12 das root tips from the genotypes indicated. Note that no cell death response was detected at any time point analysed.Data information: Images were taken in median sections. Scale bars: 50 μm, genotype as indicated in the images. White arrows: QC position in each image.

Reduced RBR levels in the quiescent centre lead to extra cell divisions and sensitivity to genotoxic agents (Cruz‐Ramirez *et al*, 2013). To investigate whether the observed cell death was associated with S‐phase progression, we quantified DNA synthesis using 5‐ethynyl‐2′‐deoxyuridine (EdU) incorporation and cell death in two Col‐0 transgenic lines with reduced RBR levels; the *35S*_*pro*_ *:amiGORBR* (*amiRBR*) line, in which an artificial miRNA against RBR is expressed constitutively (Cruz‐Ramirez *et al*, 2013), and the *RCH1::RBR* RNAi (*rRBr*) line, in which an antisense RNA is expressed locally in the root meristem (Wildwater *et al*, 2005). Both lines conferred similar phenotypes in the root with respect to extra stem cell divisions and increased S‐phase labelling (Fig 1A and C for *amiRBR*; Fig EV1A and B for *rRBr*), which correlated with accumulating cell death both in the root tip of *amiRBR* (Fig 1B and D) and in *rRBr* (Fig EV1C).

To investigate whether cell death upon RBR silencing was due to a general deregulation of cell cycle entry, or reflected a specific role of RBR in cell viability, we analysed *CYCD3.1* overexpression, which promotes cell cycle progression through RBR phosphorylation (Dewitte *et al*, 2003, 2007; Magyar *et al*, 2012; Nowack *et al*, 2012) and *E2FA* and *E2FB* overexpression, which act downstream of RBR (De Veylder *et al*, 2002; Magyar *et al*, 2005, 2012). For proper comparison of accessions, the Ler line named G54, overexpressing *CYCD3.1* (Riou‐Khamlichi *et al*, 1999; Dewitte *et al*, 2003) was introgressed into Col‐0 (Appendix Supplementary Methods). The introgressed line showed increased EdU labelling and cell division compared to Col‐0 similar to *amiRBR* (Fig 1A and C). In contrast, no cell death was observed upon *CYCD3.1* overexpression (Fig 1B and D).

Similar to *CYCD3.1*, the overexpression of *E2FA‐DPA* (De Veylder *et al*, 2002) or *E2FB‐DPA* (Magyar *et al*, 2005; Appendix Supplementary Methods) in transgenic Col‐0 lines led to extra cell divisions in the stem cell niche and EdU labelling compared to wild‐type controls (Fig EV1D). However, the cell death response remained comparable to Col‐0 (Fig EV1E). Our *CYCD3.1* and *E2F* overexpression results indicated that the cell death response is not the consequence of deregulated cell proliferation by the RBR pathway but specifically linked to reduced RBR levels.

Cell death upon RBR silencing might be a consequence of replication stress‐mediated DNA damage. To visualise DNA damage, we followed the accumulation of the phosphorylated H2AX (γH2AX) histone variant. As shown above, the extent of EdU incorporation was comparable between *amiRBR* and Col‐0*(CYCD3.1OE)*, but the frequency of nuclei with γH2AX foci was around 4 times higher in *amiRBR* (~19%) and twice as much in Col‐0*(CYCD3.1OE)* (~10%) compared to Col‐0 (~5.5%; Fig 1E and F). Collectively, our data indicated that increased DNA damage upon reduction in RBR levels is separable from cell cycle regulation and associated with cell death.

**Figure 2:**
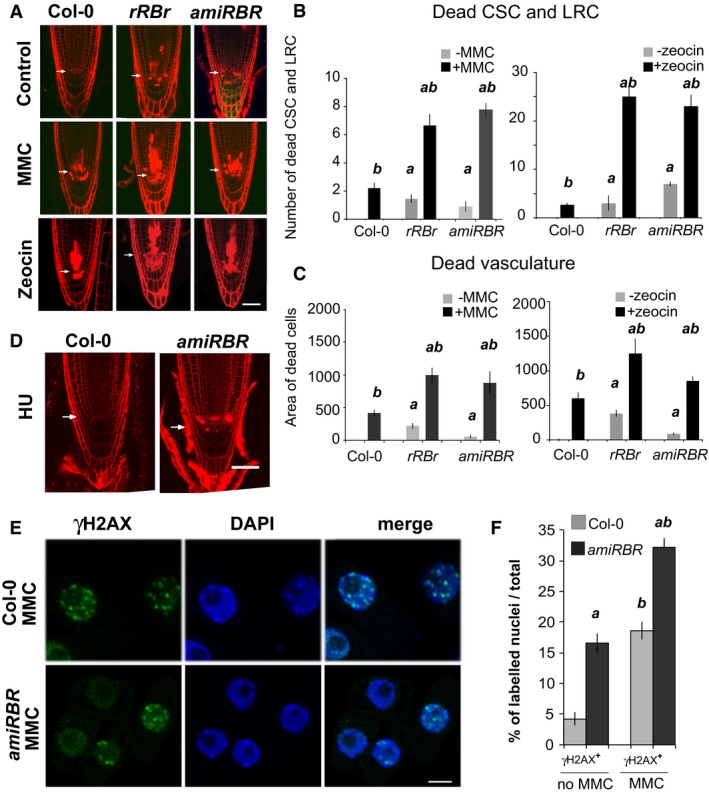
Genotoxic stress upon RBR silencing leads to hypersensitive DNA damage response ARepresentative (CM) images of Col‐0, *rRBr* and *amiRBR* root tips of 6‐ to 7‐day‐old seedlings after 16 h of mitomycin (MMC) and 20 h of zeocin treatment compared to non‐treated samples (Control).B, CCell death was quantified (B) by the number of the dead columella and lateral root cap stem cells (CSC, LRC) and their daughter cells, and (C) by measuring the area of dead vasculature above the QC in the presence of MMC for 16 h and zeocin for 20 h.DRepresentative (CM) images of Col‐0 and *amiRBR* root tips of 6‐ to 7‐day‐old seedlings after 16 h of hydroxyurea (HU) treatment compared to non‐treated samples (control shown in A).ERepresentative (CM) images of nuclei (single section) of Col‐0 and *amiRBR* 6 das root tips after 16 h of MMC treatment immune‐labelled for γH2AX (green). DAPI (blue), scale bar: 5 μm.FFrequency (%) of γH2AX foci‐harbouring nuclei compared to total nuclei in 6 das Col‐0 and *amiRBR* root tip after 16 h of MMC treatment compared to non‐treated samples.Data information: In (A and D), arrows indicate position of QC, scale bar: 50 μm. In (B, C and F), values represent mean with standard error, data are combined from *n* = 3 biological repeats, *N* > 15 roots for (B and C) and *N* > 5 in (F) of *amiRBR* and Col‐0, total nuclei > 1,000. *a* indicates significant difference within the 5 to 1% statistical confidence interval using Student's *t‐*test between *amiRBR* and *rRBr* versus Col‐0, and *b* indicates significant difference between treated versus non‐treated samples. *n *= biological repeats, *N *= samples per biological repeat.

Because RBR silencing led to spontaneous DNA damage and cell death, we tested whether the *amiRBR* line showed increased sensitivity to genotoxic stresses conferred by the DNA cross‐linker mitomycin (MMC), double‐strand break inducer zeocin, and replication stress inducer hydroxyurea (HU) (Hu *et al*, 2016). Cell death response both in *rRBr* and *amiRBR* lines were stronger than in Col‐0 upon MMC and zeocin treatments (Fig 2A–C), indicating that genotoxic stress‐induced cell death response is suppressed by RBR. In contrast, HU treatment neither triggered cell death in Col‐0 nor increased the response in *amiRBR* (Fig 2D). In line with the cell death response, the number of γH2AX‐positive nuclei upon MMC treatment increased further in the *amiRBR* line compared to Col‐0 (Fig 2E and F).

### DNA stress recruits RBR to γH2AX‐labelled heterochromatic foci

**Figure 3:**
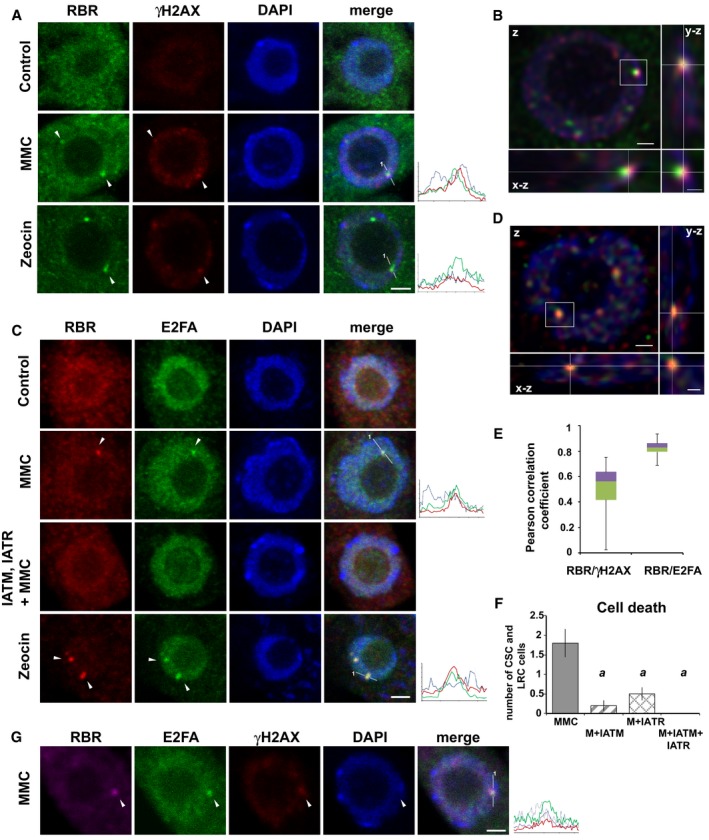
RBR and E2FA nuclear focus formation depends on ATM/ATR kinases and coincides with γH2AX‐positive sites upon MMC and zeocin treatments Representative CM images (single section) of nuclei with RBR foci at the γH2AX‐positive sites in Col‐0 upon 16 h of MMC and 3 h of zeocin treatment (white arrowheads); diffuse nuclear RBR signal is shown in the untreated control (RBR: green, γH2AX: red, DAPI: blue).Partial co‐localisation of RBR and γH2AX foci shown on Imaris section of the nucleus (RBR: green, γH2AX: red). Main panel (z) shows a single *z*‐stack of the nucleus, right panel (y‐z) shows cross section by *y* plane perpendicular to *z* plane in the main panel, lower panel (x‐z) illustrates cross section by *x* plane perpendicular to *z* plane in the main panel. Scale bar: 1 μm; scale bar of magnified insets: 0.5 μm.Representative CM images (single section) of nuclei showing accumulation of RBR (red) and E2FA‐GFP (green) in the same nuclear foci (white arrowheads) after 16 h of MMC and 3 h of zeocin treatment (RBR: red, E2FA‐GFP: green, DAPI: blue). RBR and E2FA‐GFP focus formation was not detected in untreated cells (control) or upon inhibition of ATM and ATR kinases (IATM+IATR+MMC). The activity of IATM and IATR inhibitors was followed on cell death response.Imaris section of a nucleus showing co‐localisation of RBR (red) and E2FA‐GFP (green) in foci (DAPI: blue). Main panel (*z*) shows a single *z*‐stack of the nucleus, right panel (y‐z) shows cross section by *y* plane perpendicular to *z* plane in the main panel, and lower panel (x‐z) illustrates cross‐section by *x* plane perpendicular to *z* plane in the main panel. Scale bar: 1 μm; scale bar of magnified insets: 0.5 μm.The range of Pearson correlation coefficients (PCCs) of RBR/E2FA‐ and RBR/γH2AX‐positive foci formed after 16 h of MMC treatment. PCCs are visualised in quartiles of ranked data (*n* = 30). While RBR/E2FA co‐localised in foci with high mean value of PCCs = 0.82, the RBR/γH2AX in foci showed PCCs ranging from 0.1 (side by side co‐localisation) to 0.75 (partial co‐localisation).The effect of IATM and IATR inhibitors on cell death response upon 16 h of MMC treatment in Col‐0 was quantified by the number of columella stem cells (CSC) and lateral cap stem cells (LRC) and their descendants. Values represent mean with standard deviation, *n* = 2, *N* > 15 roots for each, *a* indicates significant difference within the 5 to 1% statistical confidence interval using Student's *t‐*test comparing samples treated with inhibitors (single or combined) and MMC to MMC only.Representative CM image (single section) of a nucleus shows localisation of RBR and E2FA to a γH2AX‐positive site after 16 h of MMC treatment (white arrowheads, RBR: violet, γH2AX: red, E2FA‐GFP: green, DAPI; blue).Data information: In the intensity profiles (A, C and G), the *x*‐axis shows length in μm measured from 1 and *y*‐axis illustrates relative intensity. Scale bars: 2 μm. *n *= biological repeats, *N *= samples per biological repeat.

**Figure EV2:**
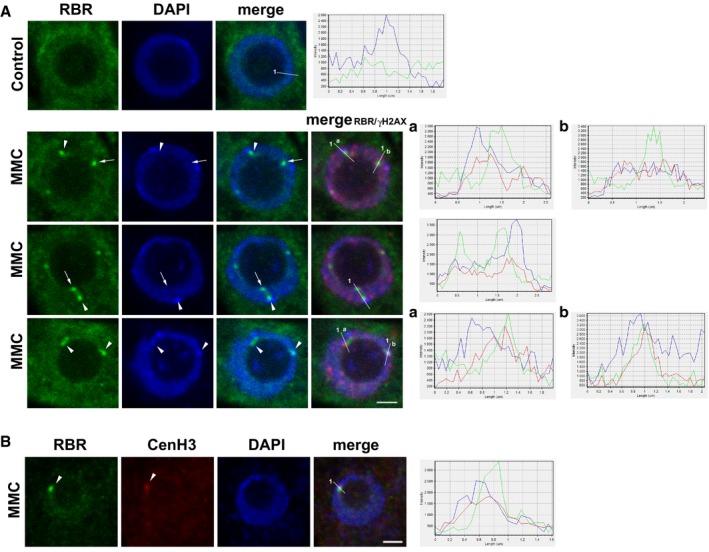
RBR nuclear foci can localise with condensed heterochromatin and CenH3 Representative CM images of nuclei (single section) of Col‐0 6 das root tips after 16 h of MMC treatment immunolabelled for RBR (green), γH2AX (red) and DAPI (blue). RBR foci localised at γH2AX‐positive sites and with DNA heterochromatin spots labelled by arrowheads, while RBR foci localised independently of condensed chromatin are marked by arrows and (a) and (b) illustrate intensity profiles for a section as given in merged images.Representative CM image of nuclei (single section) showing localisation of RBR foci to CenH3‐labelled region (arrowheads) in 6 das Col‐0 root tips after 16 h of MMC treatment (RBR: green, CenH3: red, DAPI: blue).Data information: In (A and B) intensity profiles: *x*‐axis shows length in μm measured from 1; *y*‐axis shows relative intensity. Scale bars: 2 μm. *N* > 3, *n* = 3. *n *= biological repeats, *N *= samples per biological repeat.

The role of RBR in maintaining genome stability and repressing genotoxic stress‐induced DNA damage might involve recruitment of RBR to DNA damage foci. Without genotoxic stress, RBR is diffusely localised within nuclei (Magyar *et al*, 2012; Fig 3A and C, control). MMC treatment (16 h) induced the accumulation of RBR in typically few large foci (1–5 foci per nucleus, Fig 3A and C, MMC). Around 17% of the examined nuclei contained RBR foci (total number of nuclei, *N* = 845, biological repeat, *n* = 3), mostly co‐localised with γH2AX‐positive sites (Fig 3A). 3D reconstruction of serial sections revealed a partial co‐localisation of RBR and γH2AX foci with a broad correlation range, which is typical for dynamic and transient protein interactions (Fig 3B and E). A large proportion (80%; *n* = 102) of the analysed RBR foci localised in close vicinity of heterochromatin, as confirmed by intensity profiles (Fig EV2A). The centromeric Histone 3 (CenH3) was also detected together with RBR foci upon MMC treatment (Fig EV2B).

Consistent with the localisation of tobacco E2F in genotoxic stress‐induced foci (Lang *et al*, 2012), an E2FA fusion protein under its native promoter (Henriques *et al*, 2010) significantly co‐localised with RBR in foci after treatment with genotoxic agents (Fig 3C and E). To test whether RBR and E2FA localisation to these foci depends on DNA damage signalling, we used inhibitors KU55933 for ATM (IATM) and VE‐821 for ATR (IATR), which revealed to be effective in plants by additively inhibiting the MMC‐induced cell death response (Fig 3F). The simultaneous inhibition of the ATM and ATR kinases by these drugs also abolished both RBR and E2FA focus formation (Fig 3C, IATM and IATR +MMC). In support of a role for both RBR and E2FA on DNA damage sites, RBR‐E2FA foci partially co‐localised at γH2AX‐positive sites (Fig 3G).

### RBR silencing triggers AtBRCA1 recruitment to DNA damage foci and AtBRCA1 co‐localises with RBR foci upon DNA stress

**Figure 4:**
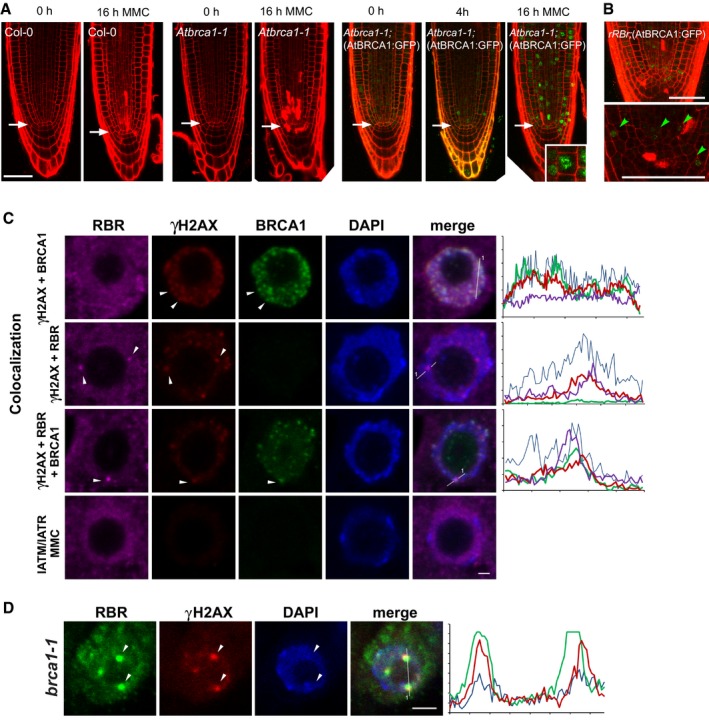
AtBRCA1 and RBR are recruited to γH2AX foci and partially co‐localise upon genotoxic stress, and locate to foci independent of each other Representative CM images of PI‐stained root tips of Col‐0, *Atbrca1‐1* (0, 16 h) and *Atbrca1‐1*(*AtBRCA1*_*pro*_ *:AtBRCA1*_*gen*_ *:GFP*) seedlings after 0, 4 and 16 h of MMC treatment. Arrows indicate position of QC, scale bar: 50 μm. Inset in the last image illustrates an enlarged nucleus with pronounced speckles.CM images of PI‐stained root tips of *rRBr;*(AtBRCA1:GFP) showing AtBRCA‐GFP accumulation into foci in QC and the stem cell niche labelled with green arrowheads. Top and bottom images represent different root tips. Scale bar: 50 μm.Representative CM images of nuclei (single section) with triple immunolabelling for RBR (violet), γH2AX (red) and AtBRCA1 (green) and stained for DAPI (blue) showing co‐localisation of AtBRCA1‐GFP with γH2AX (arrowheads), RBR with γH2AX (arrowheads) and RBR, γH2AX and BRCA‐GFP (arrowheads) after 16 h of MMC treatment. In the presence of ATM and ATR inhibitors (IATM+IATR+MMC), the γH2AX and AtBRCA1‐GFP nuclear signals and RBR foci formation were abolished. See also Table 1 for statistics.Representative CM image (single section) of RBR foci localised with γH2AX‐positive sites (arrowheads) in nuclei of *Atbrca1‐1* root meristematic cells after 16 h of MMC treatment (RBR: green, γH2AX: red, DAPI: blue).Data information: In (C and D) intensity profiles: *x*‐axis shows length in μm measured from 1; *y*‐axis shows relative intensity. Scale bars: 2 μm. *N* > 3, *n* = 3. *n *= biological repeats, *N *= samples per biological repeat.

**Figure EV3:**
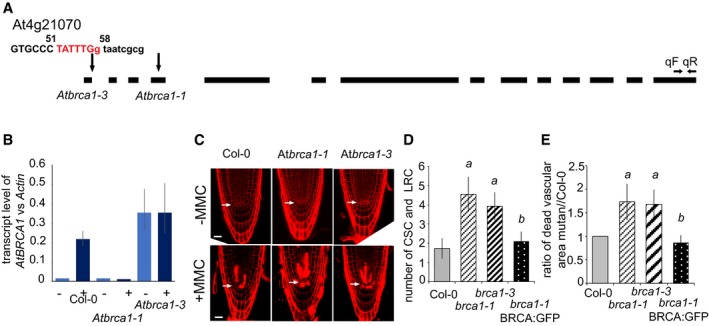
The *Atbrca1‐3* mutant, similarly to *Atbrca1‐1*, also shows hypersensitivity upon genotoxic stress APosition of the T‐DNA insertion in *Atbrca1‐1* (Reidt *et al*, 2006) and *Atbrca1‐3* mutants. The sequence indicates the insertion in *Atbrca1‐3,* and difference in letter type shows the exon–intron border. Arrows depict position of forward (qF) and reverse (qR) primers used for qRT–PCR reactions.BExpression level of the *AtBRCA1* transcript in Col‐0, *Atbrca1‐1* and *Atbrca1‐3* alleles compared to the *AtACTIN2* transcript level in Col‐0 in normal growth conditions. To control the inducibility of the transgenes, the alleles and Col‐0 were treated with MMC (+) and compared to non‐treated seedlings (−). The graph shows that genotoxic stress influenced the *AtBRCA1* transcript level only in the control, but not in the alleles. However, neither of the *Atbrca1* alleles are null alleles. *n* > 2, *N* > 100 seedlings (6 das) for each genotype and treatments.CCM images of PI‐stained root tips of Col‐0, *Atbrca1‐1* and *Atbrca1‐3* 6 das seedlings grown without (−MMC) and treated with MMC (+MMC) for 16 h. Scale bar: 20 μm, arrow: QC position in each image.D, EFunctional analysis of the (*AtBRCA1*_*pro*_ *:AtBRCA1*_*gen*_ *:GFP*) construct following cell death response in the introgressed line *Atbrca1‐1*(pgBRCA:GFP) compared to Col‐0, *Atbrca1‐1* and *Atbrca1‐3*. (D) Cell death response was quantified in the distal stem cell region after 16 h of MMC treatment. Dead columella stem and daughter cells (CSC) and lateral root initials and their descendants (LRC) were counted in median section as shown in (C);* n* = 3, *N* > 15. (E) Ratio of PI‐stained area in the proximal meristem comparing mutants and the complementing line to Col‐0. PI‐stained area was measured in each experiment from *N* > 15 mutants and Col‐0, and then means and ratio were calculated. Finally, the mean of the different experiments/ratios (*n* = 3–4) was calculated and depicted. Data Information: In (B, D and E), values represent means ± standard deviation. *a* indicates significant difference around 1% confidence using Student's *t‐*test comparing *Atbrca1‐1* and *Atbrca1‐3* to Col‐0, and *b* indicates 99% significance between *Atbrca1‐1*(AtBRCA1:GFP) and *Atbrca1‐1*. *n *= biological repeats, *N *= samples per biological repeat.

BRCA1 is a pivotal DNA repair protein of double‐strand DNA damage both in mammals (Rosen, 2013) and in *Arabidopsis* (Block‐Schmidt *et al*, 2011; Trapp *et al*, 2011). *Atbrca1‐1* (Reidt *et al*, 2006) and *Atbrca1‐3* loss of function mutants displayed hypersensitive cell death response to genotoxic stress (MMC treatment) compared to Col‐0 (Figs 4A and EV3A–E). We generated a genomic AtBRCA1‐GFP construct driven by the endogenous promoter (AtBRCA1‐GFP) (Appendix Supplementary Methods) and transformed it into the *Atbrca1‐1* line. The AtBRCA1‐GFP construct complemented the cell death response of the *Atbrca1‐1* mutant (Figs 4A and EV3D and E). In untreated *Atbrca1‐1*(AtBRCA1‐GFP) root meristems, the GFP signal was low and diffuse in the nucleus, while the signal increased upon MMC treatment and accumulated in pronounced speckles of an increasing number of meristematic nuclei in *Atbrca1‐1*(AtBRCA1‐GFP) (Fig 4A). Upon root meristem‐specific silencing of RBR in the *rRBr* line, AtBRCA1‐GFP also accumulated in nuclear speckles in and around the stem cell niche area indicating that the localisation of AtBRCA1 is induced by RBR reduction and is not critically dependent on RBR (Fig 4B).

AtBRCA1‐GFP nuclear speckles co‐localised with γH2AX foci, after MMC treatment (Fig 4C), and thus, we investigated whether RBR is co‐recruited with AtBRCA1 at γH2AX foci by triple immunoco‐localisations of RBR, AtBRCA1‐GFP and γH2AX in the *Atbrca1‐1*(BRCA1‐GFP) line (Fig 4C). Similar proportions of γH2AX‐positive nuclei showed co‐localisation of γH2AX foci either with AtBRCA1‐GFP or RBR (25 and 27%, respectively; Table 1). The AtBRCA1‐ and γH2AX‐overlapping foci were small and numerous in most nuclei and well distinguishable from the large and sparse RBR‐γH2AX co‐labelled foci. The two different classes of foci rarely coexisted within the same cell (Fig 4C, Table 1). Foci with RBR and AtBRCA1 together at γH2AX sites appeared at lower frequency (10% of the γH2AX^+^ nuclei, *N* = 452, *n* = 3; Table 1) and their appearance resembled the large RBR‐γH2AX foci. RBR and AtBRCA1 co‐localised only in the presence of γH2AX. When ATM and ATR kinase inhibitors were applied simultaneously with MMC, these inhibitors reduced the number of nuclei with γH2AX and AtBRCA1‐GFP foci and abolished the formation of RBR foci (Fig 4C).

**Table 1:** Number and ratio of nuclei showing co‐localisation of γH2AX, RBR and/or AtBRCA1

	Number of nuclei					Ratio	
	Root 1	Root 2	Root 3	Mean	SD	Mean (%)	SD
γH2AX (total)	156	144	152	151	6.1	100%	
γH2AX+ RBR	37	56	45	46	9.5	27%	3%
γH2AX+AtBRCA1	32	42	38	37	5.0	25%	4%
γH2AX+AtBRCA1+RBR	12	14	15	14	1.5	9%	1%

To test whether RBR can be recruited to γH2AX foci in the absence of AtBRCA1, we monitored RBR and γH2AX foci upon MMC treatment in the *Atbrca1‐1* mutant. We observed co‐localisation of RBR and γH2AX in large and sparse foci as in the control, suggesting that RBR recruitment is independent of AtBRCA1 (Fig 4D).

**Figure 5:**
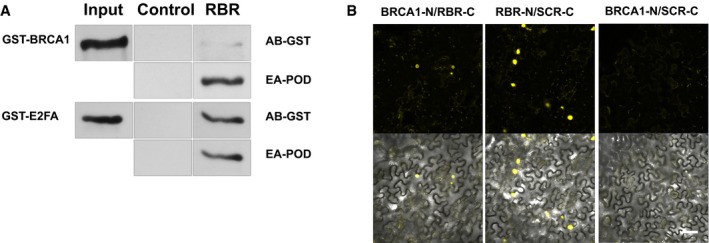
RBR and AtBRCA1 proteins can physically interact Co‐immunoprecipitation of RBR with AtBRCA1 and E2FA proteins. Control: streptavidin beads, RBR: streptavidin beads bound with RBR‐biotin, AB‐GST: GST (anti‐glutathione‐S‐transferase) antibody, EA‐POD: Extravidin‐POD (peroxidase‐conjugated streptavidin) labelling RBR‐biotin‐containing complexes, GST‐BRCA1: GST‐labelled AtBRCA1, and GST‐E2FA: GST‐labelled E2FA proteins, in the input of the wheat germ extract.BiFC assay *in planta* reveals physical interaction between AtBRCA1 and RBR (BRCA1‐N/RBR‐C). The RBR‐N/SCR‐C pair was used as a positive control, and BRCA1‐N/SCR‐C pair as a negative control. Young, growing tobacco leaves were infiltrated and analysed 36–48 h after infiltration. Scale bar: 50 μm, SCR: SCARECROW transcription factor.

To study whether AtBRCA1 and RBR proteins might physically interact, we translated both proteins *in vitro* in wheat germ extract and performed co‐immunoprecipitations (Appendix Supplementary Methods). RBR specifically interacted with AtBRCA1, but was weaker than the positive control, E2FA (Fig 5A). The observed direct interaction between RBR and AtBRCA1 was confirmed by bimolecular fluorescence complementation (BiFC) assays in young, growing tobacco leaves in the presence or absence of MMC. RBR–SCARECROW complex formation (Cruz‐Ramirez *et al*, 2012) served as positive control and AtBRCA1–SCARECROW interaction as negative control. RBR and SCARECROW formed a complex within 36 h after infiltration, while RBR and AtBRCA1 complex formation could be detected after 48 h in the nucleus (Fig 5B). In rare cases, the interaction was observed in foci. These data indicated that RBR and AtBRCA1 are independently recruited to DNA damage foci but have the ability to interact.

### RBR and AtBRCA1 genetic interaction suggests common roles in maintaining genome integrity

**Figure EV4:**
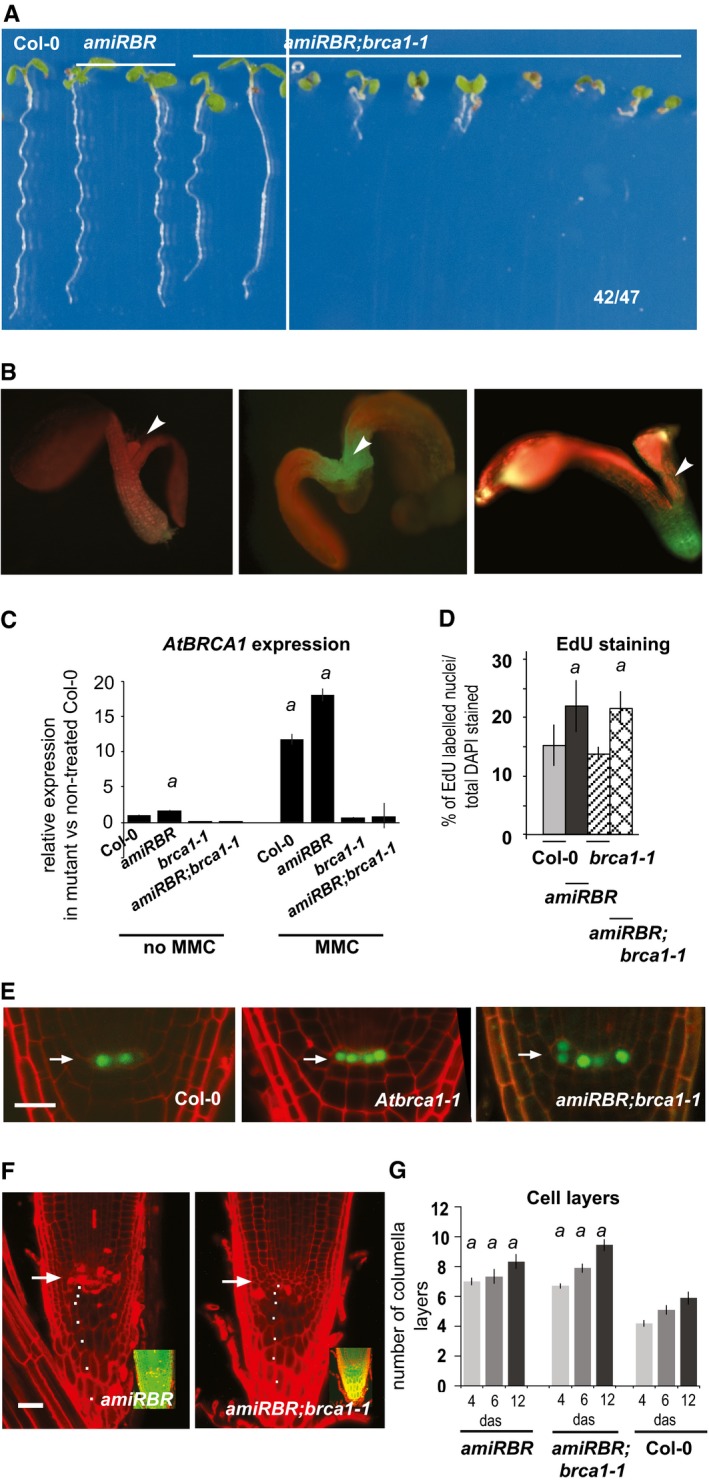
Lack of AtBRCA1 in conjunction with RBR silencing results in partially penetrant developmental arrest and suppresses cell death response, but does not influence extra stem cell division and S‐phase entry induced by RBR silencing in surviving individuals Segregation (47 severe/49 survival) and growth habit of *amiRBR;Atbrca1‐1* homozygous seedlings (F3).Developmental defects in germinating seedlings, arrowheads point to missing primary leaves.Relative transcript level of *AtBRCA1* in *amiRBR*,* Atbrca1‐1* and *amiRBR;brca1‐1* compared to Col‐0, where the level of expression was set arbitrarily to 1 in non‐treated samples. Upon MMC treatment, the graph shows the ratio of expression between treated and non‐treated samples. Values represent mean ± SD, *n* > 2, *N* > 100. *a*:* P* < 0.05 shows significant increase in expression compared to Col‐0 untreated control using Student's *t‐*test.Frequency (%) of EdU‐labelled nuclei (10‐min pulse) compared to total DAPI‐stained nuclei, *a*:* P* < 0.001, all compared to Col‐0 using Student's *t‐*test, *n* > 2, *N* > 10; error bars indicate ± SD.WOX5_*pro*_‐WOX5_*gen*_‐3xGFP expression in the mutant lines showing QC division in the *amiRBR;brca1‐1* compared to Col‐0 and *Atbrca1‐1*. Arrow indicates the position of the QC, scale bar: 20 μm.Confocal images of *amiRBR* and *amiRBR;brca1‐1* root tips of 12 das seedlings showing columella and stem cell layers (white dots). Arrow: QC position. Inset is showing the incorporation and presence of the *amiRBR* construct. Scale bar: 20 μm.Quantification of the number of columella and stem cell layers of 4‐, 6‐ and 12‐day‐old roots from *amiRBR*,* amiRBR;brca1‐1* and Col‐0. Values represent means ± SD, *N* > 15 for each mutant and Col‐0 (*n* = 3–4). *a*:* P* < 0.01 between the given genotype and Col‐0 at a given time point using Student's *t‐*test.Data information: *n *= biological repeats, *N *= samples per biological repeat.

**Figure 6:**
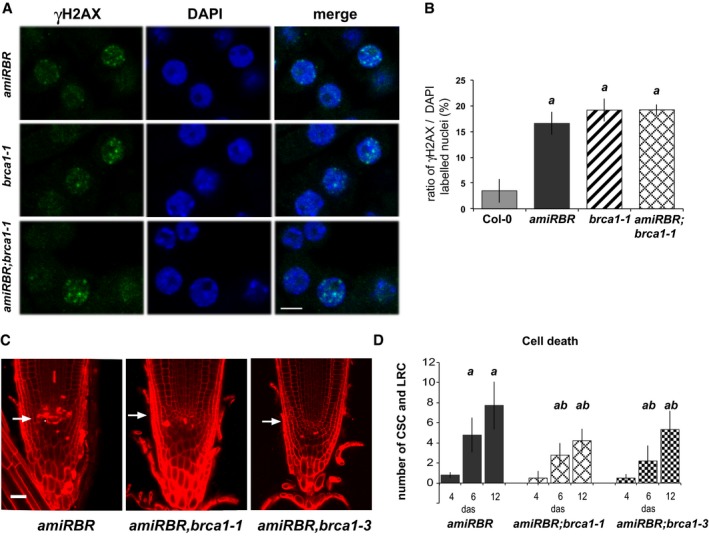
RBR and AtBRCA1 may act in a common process during DDR Representative (CM) images of nuclei (single section) of *amiRBR*,* Atbrca1‐1* and *amiRBR;Atbrca1‐1* 6 das root tips immunolabelled for γH2AX (green) and DAPI (blue). Scale bar: 5 μm.Frequency (%) of γH2AX‐labelled nuclei to total DAPI‐stained nuclei in Col‐0, *amiRBR*,* Atbrca1‐1, amiRBR;Atbrca1‐1* grown under normal conditions. Values represent means with SD, *n* = 3, and total nuclei > 1,000. *a* indicates significant difference within the 1% statistical confidence interval using Student's *t‐*test between *amiRBR, Atbrca1‐1, amiRBR;Atbrca1‐1* versus Col‐0.CM images of PI‐stained root tips from *amiRBR*,* amiRBR;brca1‐1* and *amiRBR;brca1‐3* of 12 das seedlings. Scale bar: 20 μm, arrow: QC position in each image.Cell death response of *amiRBR*,* amiRBR;brca1‐1*,* amiRBR;brca1‐3* seedlings at 4, 6 and 12 das. Values represent means with SD, *N* > 15 for each mutant and Col‐0 (*n* = 3–4) *a*:* P* < 0.01 between the given genotype and *Atbrca1‐1*, which did not develop cell death at any time point. *b*:* P* < 0.01 comparison between cross and *amiRBR*. The total number of dead columella stem and daughter cells (CSC), lateral root cap initials and their descendants (LRC) were counted in median sections as shown in (C).Data information: *n *= biological repeats, *N *= samples per biological repeat.

To study whether RBR and AtBRCA1 might function together, we studied their genetic interaction. Based on genotyping and segregation analysis of linked resistance markers, the *amiRBR;Atbrca1‐1* cross was homozygous for both loci, yet around half of the seedlings showed strong developmental abnormalities, such as mis‐positioned or missing organs or seedling lethality (Fig EV4A and B), indicating a variably penetrant window of sensitivity for the lack of AtBRCA1 and compromised RBR level during embryogenesis. The *amiRBR;Atbrca1‐1* seedlings that looked largely normal displayed extra stem cell divisions and increased S‐phase entry in the root meristem, both phenotypic confirmations for the effective RBR silencing (Fig EV4D–G), and the *AtBRCA1* expression could not be induced in the introgressed line confirming the presence of the mutant *AtBRCA1‐1* allele (Fig EV4C). The frequency of γH2AX‐positive nuclei in the *Atbrca1‐1* and *amiRBR* parents and the *amiRBR;Atbrca1‐1* cross was similar (Fig 6A and B), which is consistent with a scenario where RBR and AtBRCA1 act together in a common pathway to maintain genome integrity.

We also studied whether AtBRCA1 function is required for the cell death response observed in the *amiRBR*, and found that both in the *amiRBR,Atbrca1‐1* and *amiRBR;Atbrca1‐3* crosses, the cell death was substantially suppressed (Fig 6C), as quantified in the distal stem cell niche (Fig 6D). The lack of AtBRCA1 function had no substantial influence on other RBR‐regulated processes such as columella stem cell division or S‐phase entry (Fig EV4D–G).

To test whether *AtBRCA1* expression is sufficient to induce cell death, we expressed a *myc*‐tagged genomic *AtBRCA1* fusion under the control of the GVX β‐estradiol‐inducible promoter (*GVX1090*_*pro*_ *:AtBRCA1*_*gen*_ *:10xmyc*) in the *Atbrca1‐1* mutant. After 24 h of induction, no cell death developed, indicating that elevation of *AtBRCA1* transcription cannot trigger cell death on its own (Appendix Fig S1 and Appendix Supplementary Methods). These observations indicate that AtBRCA1 is required but not sufficient to trigger a cell death response when RBR cannot maintain genome integrity.

### RBR regulates DDR gene transcription through E2FA

**Figure 7:**
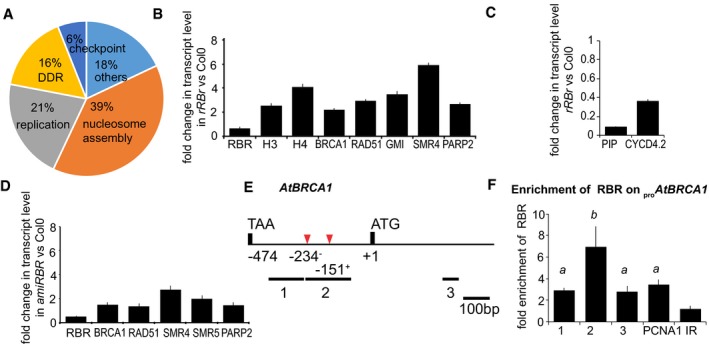
Genes regulated by RBR are annotated to nucleosome assembly, replication and DDR; RBR protein is enriched on the *AtBRCA1* promoter AThe pie chart represents the major processes regulated by RBR.B, CValidation of transcriptome analysis for a selected set of up‐ and down‐regulated genes upon RBR silencing, respectively, using qRT–PCR on dissected root tips of 4‐day‐old *rRBr* and Col‐0 seedlings.DGenes showing differential expression upon local RBR silencing are also de‐repressed in the constitutively silenced *amiRBR* line. Graph represents qRT–PCR on 4‐day‐old seedling material.ESchematic representation of the *AtBRCA1* promoter; black lines with numbers indicate the position and length of the amplified regions by qPCR analysis, the position of the start codon (ATG), the stop codon of the upstream neighbouring transcript and the position of putative E2F elements (red arrowheads) on the + and − strand, at positions −234 and −151, respectively, are indicated. Position of amplified regions: 1: −383 to −248; 2: −238 to −78; and 3: +313 to +455; positions are numbered from ATG (+1).FChromatin immunoprecipitation (ChIP) using RBR antibody; the graph shows fold enrichment calculated as a ratio of chromatin bound to the numbered section of the promoter with or without antibody. Values represent mean of three biological replicates with standard error, *a*:* P* < 0.01 compared to the negative control and *b*:* P* < 0.01 compared to the positive control using Student's *t‐*test. *PCNA1* promoter was used as a positive control and IR (an intergenic region between At3g03360‐70) as a negative control. The enrichment on IR was arbitrarily set to 1. Numbers 1, 2 and 3 on the *x*‐axis refer to the regions labelled in (E).Data information: In (B–D), values represent mean of fold change normalised to values of the relevant genes from Col‐0, and error bars indicate ± SD, *n* = 2, *N* > 100. All of the values were in the 1% statistical confidence interval using Student's *t‐*test. Abbreviations of genes are available in Appendix Table S1 and primers used in this study in Appendix Table S3. Data information: *n *= biological repeats, *N *= samples per biological repeat.

The observed recruitment of RBR together with E2FA as a complex at DNA lesions might start the signalling process for the transcriptional regulation of DNA damage response genes. To investigate transcriptional responses to RBR down‐regulation in the root tip, we performed genomewide transcriptome profiling of the meristematic region (representative root tips of each time point are shown in Fig EV1A) in three independent biological replicates (Appendix Supplementary Methods). We identified 99 differentially expressed genes between *rRBr* and Col‐0 root tips, of which 82 genes were up‐ and 17, including *RBR,* were down‐regulated (Appendix Table S1). Gene ontology (GO) analysis revealed significant enrichment for genes encoding nuclear proteins functionally related to three major processes: (i) nucleosome and chromosome assembly and maintenance; (ii) replication and cell cycle checkpoint control; (iii) DNA damage response and repair (Fig 7A, Appendix Table S1 and Appendix Supplementary Methods). The transcriptional changes in a set of genes representing different functional and co‐expressional categories (Appendix Table S2, Appendix Fig S2 and Appendix Supplementary Methods) were confirmed by qRT–PCR both in *rRBr* root tips, where *RBR* is silenced in root meristems (Fig 7B and C), and in seedlings from *amiRBR* where post‐embryonic RBR levels are reduced constitutively using the *35S* promoter (Fig 7D). The transcriptional changes were comparable in *rRBr* and *amiRBR* lines and in full agreement with the micro‐array data. Importantly, *AtBRCA1* was among the DDR targets that were up‐regulated upon RBR silencing.

The presence of canonical E2F binding sites in the 1‐kb promoter region of the differentially expressed genes (53 out of 99, summarised in Appendix Table S1 column *N*, based on Naouar *et al*, 2009) suggested that RBR exerts its repressive activity through E2F proteins. Chromatin immunoprecipitation (ChIP) assays were carried out on root tissues using RBR‐specific antibody (Horvath *et al*, 2006) in three independent biological repeats followed by qRT–PCR on the promoter of the *AtBRCA1* (Fig 7F). Significant enrichment of RBR was detected on distinct regions of the *AtBRCA1* promoter. The enrichment on fragment 2 that contained two putative E2F binding motifs (Fig 7E; −234^+^:ggggcaa and −151^−^:tttggcgc) exceeded the enrichment detected on the *PCNA1* promoter used as a positive control (Fig 7F). A reduced level of enrichment (± 3 times) was also observed in neighbouring regions lacking putative binding sites, which may be attributed either to the heterogeneous size of sonicated fragments (± 300–500 bp) or to E2F binding to non‐consensus sequences.

**Figure 8:**
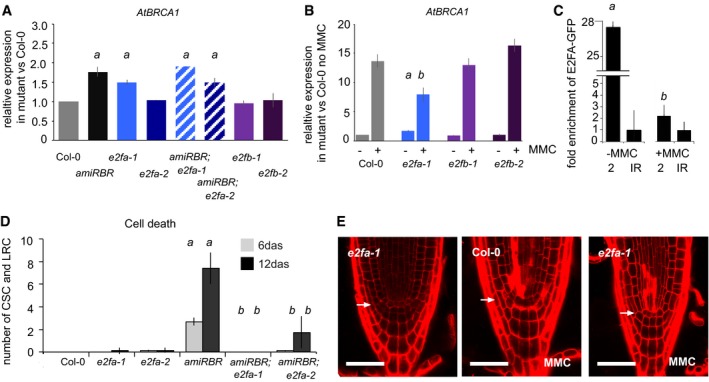
Spontaneous cell death upon RBR silencing is suppressed by E2FA and DNA damage response upon genotoxic stress is dependent on E2FA Relative transcript level of *AtBRCA1* in *amiRBR*,* e2fa‐1*,* e2fa‐2*, their double mutants and *e2fb‐1*,* e2fb‐2* compared to Col‐0, where the level of expression was set arbitrarily to 1.Relative transcript level of *AtBRCA1* in Col‐0, *e2fa‐1*,* e2fb‐1* and *e2fb‐2* without and upon 16 h of MMC treatment. All the values are compared to the expression level measured in non‐induced Col‐0 which was set to 1.ChIP using GFP antibody to chromatin isolated from Col‐0(AtE2FA‐GFP) seedlings; the graph shows fold enrichment on the *AtBRCA1* promoter region 2 without and upon genotoxic treatment (MMC, 16 h). The graph illustrates a representative experiment. *a*:* P* < 0.01 without MMC, *b*:* P* < 0.01 in MMC compared to the non‐treated and IR control using Student's *t‐*test. The enrichment on IR was arbitrarily set to 1.Quantitative analysis of cell death response in Col‐0, *e2fa‐1*,* e2fa‐2*,* amiRBR, amiRBR;e2fa‐1* and *amiRBR;e2fa‐2* mutants at 6 and 12 das. Values represent mean ± SD, at least two biological replicates testing more than 20 seedlings for each mutant. Note the absence and insignificant number of spontaneous cell death in the distal stem cell niche in Col‐0 and *e2fa* mutants, respectively, at these time points. *a*:* P* < 0.05 significance comparing single mutant to Col‐0 and *b*:* P* < 0.05 comparing double mutants to *amiRBR* using Student's *t‐*test. CSC: columella stem cells, LRC: lateral root cap initials and their descendents.CM images of PI‐stained root tips in non‐treated *e2fa‐1* mutant, and MMC‐treated Col‐0 and *e2fa‐1* (6 das). Images were taken in median section, scale bar: 50 μm. Arrow: QC position in each image.Data information: In (A and B), values represent mean ± SD, *n* > 2, *N* > 100 in each experiment. *a*:* P* < 0.05 comparing single mutant to Col‐0 and in (B) *b*:* P* < 0.05 comparing values upon MMC treatment using Student's *t‐*test. *n *= biological repeats, *N *= samples per biological repeat.

**Figure EV5:**
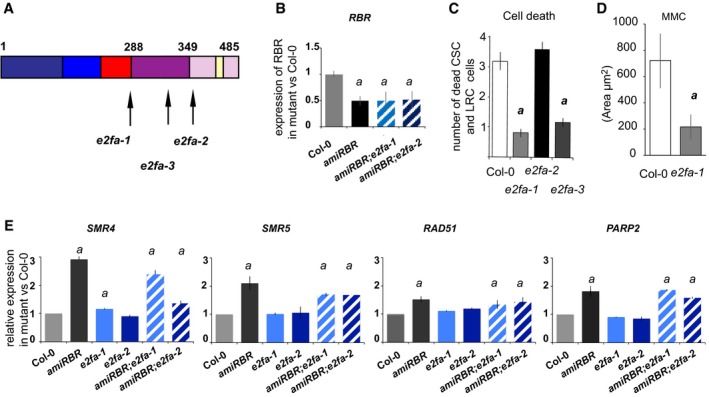
Both transcription of *AtBRCA1* and *SMR4* upon RBR silencing and cell death response upon genotoxic stress are dependent of E2FA Position of different T‐DNA insertions in AtE2FA, colours represent different domains: dark blue, N‐terminal; light blue, DNA‐binding domain; red, dimerisation domain; purple, marked box; lilac, transactivation domain; yellow, RBR binding domain. Drawing based on Magyar *et al* (2012).Relative transcript level of RBR in *amiRBR* and *amiRBR;e2fa‐1* and *amiRBR;e2fa‐2* double mutants.Cell death response in MMC‐treated 6 das seedlings of different *e2fa* alleles; total number of dead columella stem cells (CSC), lateral root cap initials (LRC) and their descendants were counted.Quantification of cell death by measuring the area of dead vasculature (μm^2^) in the presence of MMC for 16 h. No cell death response was observed in non‐treated samples.Relative transcript level of *SMR4*,* SMR5, RAD51* and *PARP2* in *amiRBR, e2fa‐1, e2fa‐2* and double mutants compared to Col‐0, where the level of expression was set arbitrarily to 1. *a*:* P* < 0.05 significance between mutant versus Col‐0 using Student's *t‐*test; values represent mean of relative expression.Data information: In (B–E), bars represent mean ± SD, *n* > 2, *N* > 10 seedlings in (C and D) and *N* > 100 in (B and E). *a*:* P* < 0.05 significance between different *e2fa* mutants versus Col‐0 using Student's *t‐*test. *n *= biological repeats, *N *= samples per biological repeat.

To address which of the activator E2Fs might partner with RBR to regulate DDR gene expression, we quantified transcription of *AtBRCA1* in *e2fa‐1*,* e2fa‐2*,* e2fb‐1* (MPIZ_244, GABI‐348E09, SALK_103138, respectively; Berckmans *et al*, 2011b) and *e2fb‐2* (SALK_120959) mutants. Similar to *amiRBR, AtBRCA1* expression increased in *e2fa‐1* but not in *e2fa‐2* mutants nor in any of the *e2fb* mutants when compared to Col‐0 (Fig 8A). The difference in the *AtBRCA1* expression in the two *e2fa* lines likely relates to the different sites of insertion in the two alleles. Both *e2fa* mutant alleles are predicted to encode truncated E2FA proteins that lack the transactivation and the canonical RBR binding domains, but retain the DNA‐binding and dimerisation domains. In contrast to *e2fa‐2*, the *e2fa‐1* allele also lost the putative “marked box” domain (Fig EV5A), which was described in mammalian E2Fs to provide a second interaction interface with Rb's C‐terminal domain (Ianari *et al*, 2009; Dick & Rubin, 2013). *AtBRCA1* derepression in *amiRBR;e2fa‐1* and *amiRBR;e2fa‐2* double homozygous lines did not exceed the derepression seen in *amiRBR*, further validating that RBR represses *AtBRCA1* through the DNA‐binding E2FA transcription factor (Fig 8A). The level of RBR silencing in the double mutants is shown in Fig EV5B. Among the RBR‐repressed DDR‐related genes tested, only *SMR4* was similarly regulated as *AtBRCA1* by RBR and E2FA (Fig EV5E). Interestingly, MMC‐induced *AtBRCA1* expression was suppressed in *e2fa‐1* but not in *e2fb* mutants (Fig 8B), suggesting that E2FA is specifically required for genotoxic stress‐induced *AtBRCA1* expression.

### RBR represses E2FA activity to inhibit cell death response

To test whether E2FA can directly bind to the *AtBRCA1* promoter, we performed ChIP analysis using *AtE2FA*_*pro*_ *:AtE2FA*_*gen*_ *:GFP* seedlings and *35S*_*pro*_ *:GFP* controls (Magyar *et al*, 2012). E2FA‐GFP was highly enriched on the segment of the *AtBRCA1* promoter containing two putative E2F binding sites (Fig 8C) and the enrichment was reduced when seedlings were treated with MMC (Fig 8C), indicating that, upon genotoxic stress, RBR‐E2FA‐mediated repression of *AtBRCA1* is released. To investigate whether the release may rely on a change in E2FA‐RBR interaction upon genotoxic stress, we pulled down the complex through the E2FA‐GFP and quantified known complex components by label‐free mass spectrometry (MS). We found that the association of RBR with E2FA and the DPs became stronger upon MMC treatment as indicated by the ratio of the quantified MS spectra of the complex components (Appendix Table S4). Interestingly, in the E2FA‐GFP pull downs we could never detect any of the components of the multi‐protein complex *D*P, *R*B‐like *E*2F and *M*uvB (DREAM, Sadasivam & DeCaprio, 2013), while with E2FB‐GFP, these proteins were readily pulled down (Appendix Table S5). This may suggest that E2FA functions in different complex(es) than the DREAM associated with E2FB and E2FC (Kobayashi *et al*, 2015).

As RBR repression acts through E2FA to regulate transcription of at least two DDR genes, we investigated whether this regulation functions also in the cell death response. We quantified cell death in the two *e2fa* mutant lines alone and in combination with *amiRBR*. Neither *e2fa‐1* nor *e2fa‐2* showed any cell death response, and root development was also normal. Importantly, spontaneous cell death in *amiRBR* was completely suppressed in the *amiRBR;e2fa‐1* and strongly delayed and reduced in the *amiRBR;e2fa‐2* crosses (Fig 8D) while RBR silencing remained effective (Fig EV5B), demonstrating that the RBR silencing‐induced cell death response is dependent on E2FA function. To test whether E2FA is also required for genotoxic stress‐induced cell death, we treated *e2fa* mutants with MMC. Cell death upon genotoxic stress was partially suppressed in *e2fa‐1* and *e2fa‐3* (Xiong *et al*, 2013) lines, but not by *e2fa‐2* (Fig 8E and quantified in Fig EV5C and D), confirming that the cell death is generally dependent on E2FA and is mediated through the marked box.

### E2FA and RBR are required for genotoxic stress‐induced DDR in a SOG1‐independent pathway

**Figure EV6:**
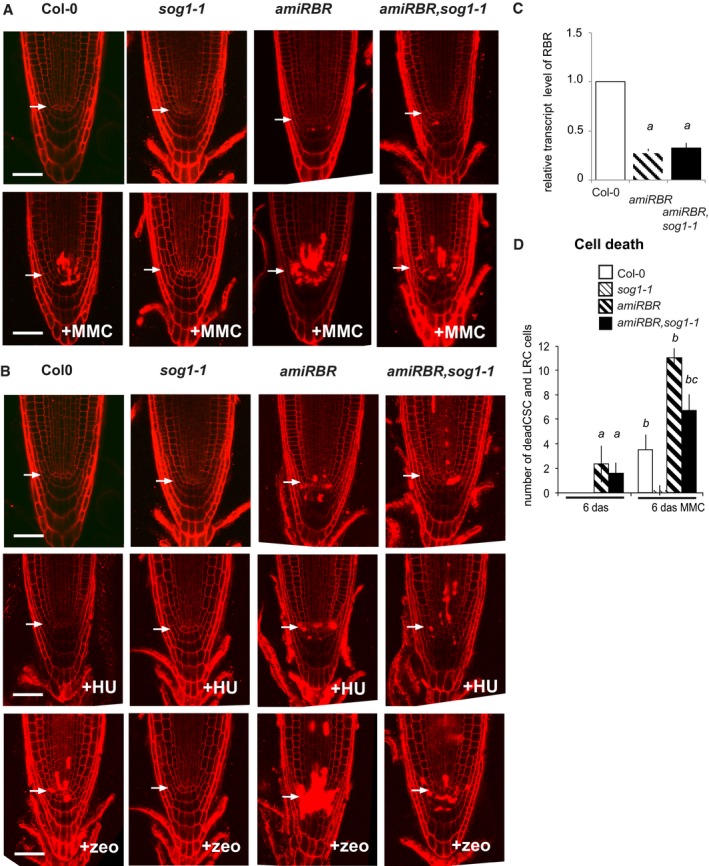
Hypersensitive cell death response after genotoxic stress only partially depends on SOG1 upon RBR silencing CM images of PI‐stained root tips from *sog1‐1*,* amiRBR* and *amiRBR,sog1‐1* lines compared to Col‐0 as indicated above the columns. Images were taken in median section of 6 das seedlings treated with and without MMC (10 μg/ml). Scale bar: 50 μm, arrow: QC position in each image.Representative images of 9 das seedlings showing cell death response after hydroxyurea (+HU, 1 mM) or zeocin (+zeo, 20 μg/ml) treatment. Scale bar: 50 μm, arrow: QC position in each image.Relative *RBR* transcript level in *amiRBR* and *amiRBR,sog1‐1* lines, taking mean of several independent lines. *a*:* P* < 0.05 significance genotypes versus Col‐0 using Student's *t‐*test.Cell death response upon MMC treatment in *sog1‐1*,* amiRBR* and *amiRBR,sog1‐1* lines compared to Col‐0. The total number of dead columella stem and daughter cells (CSC), lateral root cap initials (LRC) and their descendants were counted in median section as shown in (A). *a*:* P* < 0.05 significance genotypes versus Col‐0, *b*:* P* < 0.05 comparison of treated samples to non‐treated counterparts, *c*:* P* < 0.05 significance between *amiRBR* versus *amiRBR,sog1‐1* using Student's *t‐*test. Note, that Col‐0 at 6 das and *sog1‐1* at 6 and 12 das developed no dead cells.Data information: Values represent mean ± SD, *n* = 3, *N* > 15 in (A, B and D) and *N* > 100 in (C) for each mutant and Col‐0. *n *= biological repeats, *N *= samples per biological repeat.

**Figure 9:**
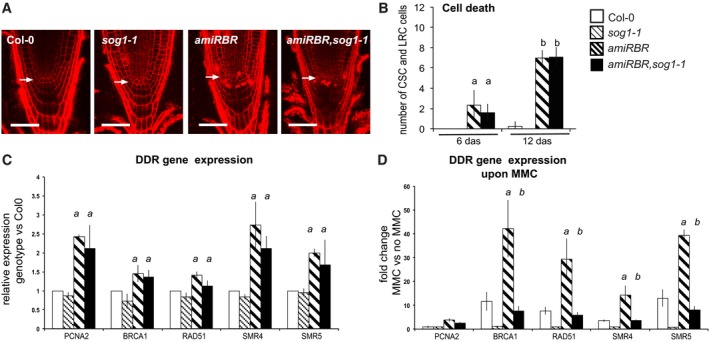
DNA damage response upon RBR silencing is independent of SOG1 CM images of PI‐stained root tips (12 das seedlings) from the genotypes indicated. Arrow indicates the position of the QC; scale bar: 50 μm.Cell death response from 6 and 12 das seedlings, total number of dead columella stem cells (CSC), lateral root cap initials (LRC) and their descendants were counted as shown in (A). Note that neither Col‐0 at 6 das nor *sog1‐1* at 6 and 12 das showed cell death. *a*:* P* < 0.05 at 6 das, *b*:* P* < 0.05 at 12 das mutants versus Col‐0 using Student's *t‐*test. Value represents mean ± SD, *n* > 3, *N* > 15 for each mutant and Col‐0. The genotype legend in the graph also holds for (C and D).Relative expression level of DDR genes in *sog1‐1*,* amiRBR* and *amiRBR,sog1‐1* lines (6 das) compared to Col‐0 (6 das), where the level of expression for each gene was set arbitrarily to 1. *a*:* P* < 0.05, mutants compared to Col‐0 using Student's *t‐*test.Transcriptional induction of the indicated genes is depicted as fold change comparing MMC (16 h) to non‐treated samples of Col‐0, *sog1‐1*,* amiRBR* and *amiRBR,sog1‐1*. *a*:* P* < 0.05 *amiRBR* versus Col‐0 and *b*:* P* < 0.05 *amiRBR,sog1‐1* to *sog1‐1* using Student's *t‐*test.Data Information: In (C) and (D), 6 das seedlings were analysed and data represent means with ± SD. At least three biological replicates were analysed, in each case around 100 seedlings for each mutant. For *amiRBR,sog1‐1* the mean was calculated from the analysis of six independent transformants (T2 generation). *n *= biological repeats, *N *= samples per biological repeat.

SOG1 is a pivotal transcription factor for the induction of DDR genes upon genotoxic stress. We observed significant overlap between DNA repair genes regulated by RBR (Appendix Table S1 column B) and genes with compromised induction by irradiation in the *sog1‐1* mutant (Appendix Table S1 columns B and L, respectively; ratio: 8/10). The *sog1‐1* mutation can fully suppress cell death response upon genotoxic stress (Yoshiyama *et al*, 2009, 2013a). Based on this comparison, we asked whether the activation of the DNA damage response pathway upon RBR silencing is dependent on SOG1 function. Homozygous *sog1‐1* plants (Preuss & Britt, 2003) were transformed with the *35S*_*pro*_ *:amiGORBR* construct (Cruz‐Ramirez *et al*, 2013), and RBR silencing was confirmed in the *amiRBR*,*sog1*‐*1* line (Fig EV6C). The cell death response in the *amiRBR*,*sog1‐1* root meristem was comparable to the *amiRBR* line (Fig 9A and B), demonstrating that cell death induced upon RBR silencing is independent of SOG1. In the *amiRBR*,*sog1*‐*1* lines, RBR silencing was effective (Fig EV6C) and transcription of all the tested DDR genes also remained elevated as in *amiRBR* (Fig 9C), showing that the release of RBR‐mediated transcriptional repression is also SOG1 independent. As expected, the genotoxic stress‐induced DDR gene expression (Fig 9D) and cell death response by MMC (Fig EV6A and D) and zeocin (Fig EV6B) treatments were fully suppressed in the *sog1‐1* plants, but not in the *amiRBR*,*sog1*‐*1* lines, further confirming that RBR acts on a SOG1‐independent pathway. HU does not activate SOG1 to induce DDR (Yoshiyama *et al*, 2013a), and hence accordingly did not have any effect in the *amiRBR* and *amiRBR*,*sog1*‐*1* lines (Fig EV6B). Taken together, RBR regulates DDR gene transcription and cell death at least in part through a SOG1‐independent pathway.

## Discussion

Here we show that the plant Retinoblastoma homologue, RBR, has a direct role in maintaining genome integrity. RBR is recruited to a limited number of large heterochromatic DNA damage foci together with E2FA in an ATM‐ and ATR‐dependent manner upon DNA damage. AtBRCA1 and RBR are independently recruited to these specific damage foci, they interact and our genetic study show that they partially act together to maintain genome integrity and to prevent cell death. The accompanying paper of Biedermann *et al* (2017) shows that RBR is required to localise RAD51 and that RBR and RAD51 co‐localise in these large foci, corroborating a non‐transcriptional role for RBR in the maintenance of plant genome integrity. We further show that RBR and E2FA also have transcriptional DNA damage response roles that act in parallel to the well‐established SOG1 pathway. Below, we discuss how this evidence for a possible dual cell cycle‐independent role of RBR in the meristematic DNA damage response at γH2AX foci and at target gene promoters fits in with recent evidence from plant and mammalian experimental systems.

### RBR‐mediated DNA damage control at γH2AX foci

When mammalian Rb proteins are deregulated, aberrant S‐phase progression can result in nucleotide pool deficiency, replication fork stalling and DNA damage (Bester *et al*, 2011). Even with haploid wild‐type Rb, mammalian cells show chromosome defects and aneuploidy (Coschi *et al*, 2014; Hinds, 2014). Similarly, in plants, reduction of RBR function in the *rbr‐3* mutant (Johnston *et al*, 2010) and in transgenic plants overexpressing the viral RepA protein inactivating RBR (Henriques *et al*, 2010) resulted in aneuploidy. Our data corroborate that, in plants, S‐phase progression due to RBR deregulation can contribute to DNA damage but we show that this effect is separable from a direct role of RBR in DNA damage control.

Our finding that DNA damage induces ATM/ATR‐dependent recruitment of RBR and E2FA to γH2AX foci suggests a direct, non‐transcriptional role for RBR in DNA damage control. The reported accumulation of *Nt*E2F in γH2AX‐labelled foci at the G1/S transition in tobacco cells is consistent with this notion (Lang *et al*, 2012). Also, consistent with non‐transcriptional roles for RBR is the finding that, during meiosis, RBR is recruited to chromosomes in a DNA DSB‐dependent manner, where it was suggested to facilitate the assembly of chromatin modifiers, repair proteins and condensin complexes for homologous recombination through their LxCxE motifs (Chen *et al*, 2011).

In mammals, Rb localises to chromatin at S‐phase after DNA damage (Avni *et al*, 2003). Furthermore, E2F1 (Coschi *et al*, 2014) and E2F7 (Zalmas *et al*, 2013) have transcription‐independent roles to bring protein complexes to damaged DNA. E2F1 and Condensin II are recruited by pRb to the pericentromeric region of the chromosome and to replication origins, thus facilitating correct replication, accurate chromosome condensation, and chromosome segregation (Coschi *et al*, 2014; Hinds, 2014). Rb heterozygosity leads to loss of E2F1 and Condensin II binding, accompanied by replication stress labelled by increased γH2AX foci. Recently, it was shown that Rb localises to DSBs dependent on E2F1 and ATM, to promote DSB repair through homologous recombination (Velez‐Cruz *et al*, 2016). We show that a similar mechanism might operate in plants, where RBR localisation to DSBs requires ATM and ATR activities. Further similarities to the animal scenario are that, homogenously distributed tobacco E2F partly relocalises upon genotoxic stress and forms 2–3 foci per nucleus in BY‐2 tobacco cells. For this focus formation, the transactivation domain and the RBR binding site were shown to be critical. Also, the plant Condensin complex II appears to play a role in alleviating DNA damage by HR or compacting the genome in response to genomic stress (Sakamoto *et al*, 2011). In future, it will be interesting to investigate whether a similar non‐transcriptional role for RBR, E2FA and Condensin II complexes in genome integrity is also operational in plants.

In mammalian cells, Rb interacts with HsBRCA1, which was suggested to be important to repress cell proliferation (Aprelikova *et al*, 1999). In *Arabidopsis,* we did not detect any cell proliferation effect either after induced *AtBRCA1* overexpression or in the *Atbrca1* mutants. In human cells, Rb was also shown to recruit HsBRCA1 in order to facilitate processing and repair of topoisomerase II‐induced DSB (Xiao & Goodrich, 2005). Recently, Rb was also shown to be directly involved in DSB repair, independently of its cell cycle function, through its interaction with components of the canonical non‐homologous end‐joining repair pathway (Cook *et al*, 2015). It will be interesting to investigate the mechanism of RBR and AtBRCA1 interaction at these specific heterochromatic sites with damaged DNA, and their joint function in DNA damage control in *Arabidopsis*.

### RBR‐mediated transcriptional responses to DNA damage

Cells with excessive damage are eliminated. The coordination of cell proliferation and apoptosis in mammalian cells relies on the formation of the Rb‐E2F1 complex by interaction of Rb's carboxy‐terminal domain and the marked box of E2F1 (Carnevale *et al*, 2012; Dick & Rubin, 2013). During S‐phase, the phosphorylated Rb‐E2F1 complex represses pro‐apoptotic genes, while in response to DNA damage upon ATM‐dependent phosphorylation of E2F1, this complex becomes a transcriptional repressor on the cell cycle genes and activator on the pro‐apoptotic genes (Ianari *et al*, 2009; Dick & Rubin, 2013). There are indications that a similar mechanism may function in *Arabidopsis*. RBR forms a complex with E2FA, which remains stable upon CYCD3;1‐CDKA phosphorylation during the cell cycle (Magyar *et al*, 2012). In animal cells, phosphorylation of Rb by CycD:Cdk4/6 kinases diversifies rather than merely inactivates Rb complexes (Narasimha *et al*, 2014). RBR phosphorylation upon CYCD3.1 overexpression in plants might similarly lead to the formation of distinct regulatory complexes with roles in activation of G1 to S transition and roles protecting against cell death or differentiation. In agreement, we find that silencing of RBR leads to a very different outcome than RBR phosphorylation. It initiates cell death response fully relying on E2FA with an intact “marked box” domain, suggesting a conserved mechanism between kingdoms. Importantly, not all the RBR‐repressed DDR genes are E2FA regulated. AtBRCA1 is an essential target, as its function was required but not sufficient to induce cell death upon transcriptional derepression. As cell death response was fully suppressed in *e2fa‐1*, in relation to AtBRCA1, additional genes should be involved in the induction of cell death process. Interestingly, both AtBRCA1 and E2F functions are required also for the pathogen‐induced cell death during hypersensitive response in plant defence (Bao & Hua, 2015; Zebell & Dong, 2015).

Active SOG1 is the pivotal transcription factor in plant DDR upon genotoxic stress (Yoshiyama *et al*, 2013b). Here we show that E2FA also carries out this function, since MMC‐induced activation of DDR genes, such as *AtBRCA1* and *SMR4,* is compromised both in *sog1‐1* and *e2fa‐1* mutants. The ability of E2FA to activate DDR genes is dependent on RBR levels or activity, which are responsive to intrinsic cell cycle‐dependent and cell extrinsic signals in a SOG1‐independent pathway.

In conclusion, RBR, mainly known as a regulator of cell cycle and asymmetric cell division in plant meristems, is also involved in maintaining genome integrity in these growth zones through two functions, (i) assembly at a limited number of γH2AX foci together with E2F and, possibly, AtBRCA1; (ii) transcriptional regulation of important DDR genes including *AtBRCA1*. It will be interesting to investigate in the future whether and how assembly of E2F‐RBR complexes at particular γH2AX foci is coupled to the transcriptional role of these complexes in DDR gene regulation.

## Materials and Methods

### Plant material and growth conditions

Seeds were sterilised and grown as described earlier (Wildwater *et al*, 2005) except that seedlings used for micro‐array analysis and qRT–PCR were germinated on 1.2% plant agar. *Arabidopsis thaliana* ecotype Columbia 0 (Col‐0) was used as wild type; T‐DNA insertion lines *Atbrca1‐3* (SALK_099751) and *e2fb‐2* (SALK_120959) were obtained from the Nottingham *Arabidopsis* Stock Centre. The transgenic lines *sog1‐1* (Yoshiyama *et al*, 2009), *e2fa‐1, e2fa‐2* and *e2fb‐1* (MPIZ_244, GABI‐348E09, SALK_103138, respectively (Berckmans *et al*, 2011b) (Berckmans *et al*, 2011a))*, e2fa‐3* (Xiong *et al*, 2013), *Atbrca1‐1* (Reidt *et al*, 2006), *rRBr* (Wildwater *et al*, 2005) and *amiRBR* (Cruz‐Ramirez *et al*, 2012) were described earlier. The T‐DNA insertions and mutations were confirmed by PCR‐based genotyping or sequencing and gene silencing was demonstrated via gene expressional studies and phenotyping. To study the *amiRBR;sog1‐1* phenotype, more than 20 independent transformants were generated, genotyped by sequencing the *sog1‐1* locus and analysed for *RBR* silencing. The overexpression lines *E2FA‐DPA* (De Veylder *et al*, 2002) and *CYCD3.1OE* (Riou‐Khamlichi *et al*, 1999; Dewitte *et al*, 2003) were described earlier. The construction of *E2FB‐DPA* (Magyar *et al*, 2005) is described in the Appendix Supplementary Methods.

### Chemical treatments and induction studies

To induce DNA damage response, 5‐ to 6‐day‐old seedlings were transferred to tissue culture plates (unless stated otherwise), containing fresh MS liquid medium without or with 10 μg/ml mitomycin C (MMC), 20 or 3 μg/ml zeocin or 1 mM hydroxyurea (HU) and treated for 16 h or alternatively for short treatment periods of 1–4 h. For kinase inhibitory assay, 5‐ to 6‐day‐old seedlings were pre‐incubated for 2 h in ATM or ATR kinase inhibitors (Selleckchem, KU55933, VE‐821, respectively) which was applied to the MS liquid medium at 10 μM final concentration, afterwards MMC was given, as described above. Appropriate controls and mutants were treated simultaneously, and all treatments were repeated at least three times (*n* = biological repeat) with 15–20 (*N* = sample size) replicates. Although the level of MMC induction was varied between the different experiments, the ratio between controls and treated samples were comparable. Cell death in root tips was quantified by counting the number of PI‐stained cells in the columella stem cells (CSC) and lateral root cap initials (LRC) and their daughter cells, and by measuring the contiguously PI‐stained cell area directly adjacent to the QC in the proximal meristematic vasculature.

### Immunofluorescence labelling and fluorescence microscopy

Root excision and slide preparation of squashed root tips and immunolabelling with *Arabidopsis* anti‐γH2AX and others were performed according to Amiard *et al* (2010) and Friesner *et al* (2005) with slight modifications; 3.7% paraformaldehyde with 0.05% Triton was used for 1 h and enzyme treatment was applied on root tips transferred and attached to microscopic slides. For dilution of primary and secondary antibodies, see Appendix Supplementary Methods. 5‐Ethynyl‐2′‐deoxyuridine (EdU) labelling was performed in whole mount preparation of root tips (for details, see also in Appendix Supplementary Methods).

For fluorescence microscopy Olympus IX‐81 FV‐1000 confocal imaging system was used. For details of confocal laser scanning microscopy, image acquisition and processing, see Appendix Supplementary Methods. For Imaris section, *z*‐stacks were taken with 0.2 μm *z*‐step. Images were de‐convolved using Huygens (Scientific Volume Imaging, Hilversum, The Netherlands) to remove out‐of‐focus information and sectioning of gained 3D objects was performed using Imaris software (Bitplane) in the section mode. The quantitative co‐localisation analysis was performed using ImageJ software with JACoP (Just Another Co‐localisation Plug‐in, (Bolte & Cordelieres, 2006) based on Pearson's coefficient. A region of interest was defined by a square of unified pixel size (26 × 26), and image correlation analysis was performed by combining single stacks of green and red fluorescent images. The data analysis was generated using the Real Statistics Resource Pack software (Charles Zaiontz; www.real-statistics.com).

For phenotypic analysis, roots were stained in 5 μg/ml propidium iodide (PI) and analysed on Leica SP2 or Olympus IX‐81‐FV1000 inverted laser scanning microscope. For qualitative and quantitative comparison, images were recorded with identical microscope settings in all cases. EdU staining of replicating cells was performed using Click‐iT EdU Alexa Fluor 488 HCS Assay (Molecular Probes, Eugene, OR, USA) as described earlier (Vanstraelen *et al*, 2009).

### Bimolecular fluorescent complementation and transient transfection assay

For BiFC, AtBRCA1 cDNA was subcloned to pGEMT‐easy 221 (see primers Appendix Table S3). Subcloning of SCR and RBR cDNAs were described earlier (Welch *et al*, 2007) (Cruz‐Ramirez *et al*, 2012) respectively). To generate split YFP construct, the binary BiFC GATEWAY‐Destination vectors were used (Gehl *et al*, 2009). Four‐week‐old *Nicotiana benthamiana* plants were infiltrated by *Agrobacterium tumefaciens* containing different constructs as described by (Liu *et al*, 2010). The infiltrated region of the leaf was then mounted in water and checked for expression. YFP fluorescence was visualised using a Zeiss LSM 710 confocal laser scanning microscope, and images were processed with the confocal microscope Zeiss ZEN software. Results from at least three independent experiments, and more than 20 infiltrated leaves were visualised.

### Transcriptional profiling, expressional studies and cloning

A detailed description is provided in Appendix Supplementary Methods. The micro‐array data are submitted to GEO under accession GSE47715. Link: http://www.ncbi.nlm.nih.gov/geo/query/acc.cgi?acc=GSE47715.

### Chromatin immunoprecipitation (ChIP)

ChIP was carried out on root material of 5‐day‐old Col‐0 seedlings to study RBR enrichment and on Col‐0(*E2FA*_*pro*_ *:E2FA*_*gen*_ *:GFP*) (Magyar *et al*, 2012) seedlings without and with 16‐h MMC treatment to analyse E2FA‐GFP enrichment. Here, *35S*_*pro*_ *:GFP* was used as a control. To determine RBR enrichment, IP was performed in the absence and presence of antibody specific for RBR protein as described by Horvath *et al* (2006). For the detection of E2FA‐GFP, GFP‐trap beads (Chromotek) were used as described earlier (Schepers *et al*, 2001).

Primers for quantitative RT/PCR were designed to amplify fragments between 100 to 200 bp spanning the putative promoter region of *AtBRCA1*. The negative and positive controls are described earlier (Cruz‐Ramirez *et al*, 2012). Primer pairs were analysed on the same biological material, repeated three times with three technical replicates for RBR and twice for E2FA. Enrichment for RBR was calculated by comparing the PCR data derived from immunoprecipitation samples with and without antibody and for E2FA between Col‐0(*E2FA*_*pro*_ *:E2FA*_*gen*_ *:GFP*) and *35S*_*pro*_ *:GF*P lines. Student's *t*‐tests were performed to analyse statistical significance. List of primers is given in Appendix Table S3.

### *In vitro* translation and pull‐down

Full‐length cDNAs for AtBRCA1 and RBR were obtained from the RIKEN Plant Science Centre and recloned into the pEU3II‐HLICNot vector by ligation‐independent cloning. *In vitro* transcription, cell‐free translation, pull‐down and immunoblotting were performed as described earlier (Nagy *et al*, 2015). See also Appendix Supplementary Methods.

## Author contributions

BMH and BS conceived the idea of analysing RBR targets and regulation of cell death, BMH, ZM and LB the links to E2Fs and BMH link to *sog1‐1*. BMH performed expressional studies, transcriptome analysis, generation and characterisation of mutants ChiP with RBR on *AtBRCA1* promoter; the immunolocalisation and microscopical studies on localisation of RBR, E2FA, γH2AX and AtBRCA1 were performed by HK and PB, the expressional and phenotypic studies by EN, experiments on RBR and AtBRCA1 binding in wheat germ system by SN and TM and using BiFC technique by IB, the analysis of the micro‐array data by BMH and GFS‐P. ZA performed the cell death experiments with *CYCD3.1OE* line. ChIP analysis with E2FA‐GFP protein was carried out by CP and SP generated and analysed the *amiRBR,e2fa* mutant. Protein complex isolation and LC‐MS/MS identification were performed by AP‐S and ZD. The manuscript was written by BMH, LB, PB and BS and seen and commented by all authors.

## Conflict of interest

The authors declare that they have no conflict of interest.

## Supporting information



Appendix

Expanded View Figures PDF

Review Process File

## References

[embj201694561-bib-0001] Amiard S , Charbonnel C , Allain E , Depeiges A , White CI , Gallego ME (2010) Distinct roles of the ATR kinase and the Mre11‐Rad50‐Nbs1 complex in the maintenance of chromosomal stability in *Arabidopsis* . Plant Cell 22: 3020–3033 20876831 10.1105/tpc.110.078527 PMC2965537

[embj201694561-bib-0002] Amiard S , Gallego ME , White CI (2013) Signaling of double strand breaks and deprotected telomeres in *Arabidopsis* . Front Plant Sci 4: 405 24137170 10.3389/fpls.2013.00405 PMC3797388

[embj201694561-bib-0003] Aprelikova ON , Fang BS , Meissner EG , Cotter S , Campbell M , Kuthiala A , Bessho M , Jensen RA , Liu ET (1999) BRCA1‐associated growth arrest is RB‐dependent. Proc Natl Acad Sci USA 96: 11866–11871 10518542 10.1073/pnas.96.21.11866 PMC18378

[embj201694561-bib-0004] Avni D , Yang H , Martelli F , Hofmann F , ElShamy WM , Ganesan S , Scully R , Livingston DM (2003) Active localization of the retinoblastoma protein in chromatin and its response to S phase DNA damage. Mol Cell 12: 735–746 14527418 10.1016/s1097-2765(03)00355-1

[embj201694561-bib-0005] Bao Z , Hua J (2015) Linking the cell cycle with innate immunity in *Arabidopsis* . Mol Plant 8: 980–982 25843011 10.1016/j.molp.2015.03.013

[embj201694561-bib-0006] Berckmans B , Lammens T , Van Den Daele H , Magyar Z , Bogre L , De Veylder L (2011a) Light‐dependent regulation of DEL1 is determined by the antagonistic action of E2Fb and E2Fc. Plant Physiol 157: 1440–1451 21908689 10.1104/pp.111.183384 PMC3252145

[embj201694561-bib-0007] Berckmans B , Vassileva V , Schmid SP , Maes S , Parizot B , Naramoto S , Magyar Z , Alvim Kamei CL , Koncz C , Bogre L , Persiau G , De Jaeger G , Friml J , Simon R , Beeckman T , De Veylder L (2011b) Auxin‐dependent cell cycle reactivation through transcriptional regulation of *Arabidopsis* E2Fa by lateral organ boundary proteins. Plant Cell 23: 3671–3683 22003076 10.1105/tpc.111.088377 PMC3229142

[embj201694561-bib-0008] Bester AC , Roniger M , Oren YS , Im MM , Sarni D , Chaoat M , Bensimon A , Zamir G , Shewach DS , Kerem B (2011) Nucleotide deficiency promotes genomic instability in early stages of cancer development. Cell 145: 435–446 21529715 10.1016/j.cell.2011.03.044 PMC3740329

[embj201694561-bib-0009] Biedermann S , Harashima H , Chen P , Heese M , Bouyer D , Sofroni K , Schnittger A (2017) The retinoblastoma homolog RBR1 mediates localization of the repair protein RAD51 to DNA lesions in *Arabidopsis* . EMBO J 36: 1279–1297 10.15252/embj.201694571 PMC5412766 28320735

[embj201694561-bib-0010] Block‐Schmidt AS , Dukowic‐Schulze S , Wanieck K , Reidt W , Puchta H (2011) BRCC36A is epistatic to BRCA1 in DNA crosslink repair and homologous recombination in *Arabidopsis thaliana* . Nucleic Acids Res 39: 146–154 20817926 10.1093/nar/gkq722 PMC3017590

[embj201694561-bib-0011] Bolte S , Cordelieres FP (2006) A guided tour into subcellular colocalization analysis in light microscopy. J Microsc 224: 213–232 17210054 10.1111/j.1365-2818.2006.01706.x

[embj201694561-bib-0012] Carnevale J , Palander O , Seifried LA , Dick FA (2012) DNA damage signals through differentially modified E2F1 molecules to induce apoptosis. Mol Cell Biol 32: 900–912 22184068 10.1128/MCB.06286-11 PMC3295199

[embj201694561-bib-0013] Chen Z , Higgins JD , Hui JT , Li J , Franklin FC , Berger F (2011) Retinoblastoma protein is essential for early meiotic events in *Arabidopsis* . EMBO J 30: 744–755 21217641 10.1038/emboj.2010.344 PMC3041947

[embj201694561-bib-0014] Ciccia A , Elledge SJ (2010) The DNA damage response: making it safe to play with knives. Mol Cell 40: 179–204 20965415 10.1016/j.molcel.2010.09.019 PMC2988877

[embj201694561-bib-0015] Cimprich KA , Cortez D (2008) ATR: an essential regulator of genome integrity. Nat Rev Mol Cell Biol 9: 616–627 18594563 10.1038/nrm2450 PMC2663384

[embj201694561-bib-0016] Cook R , Zoumpoulidou G , Luczynski MT , Rieger S , Moquet J , Spanswick VJ , Hartley JA , Rothkamm K , Huang PH , Mittnacht S (2015) Direct involvement of retinoblastoma family proteins in DNA repair by non‐homologous end‐joining. Cell Rep 10: 2006–2018 25818292 10.1016/j.celrep.2015.02.059 PMC4386026

[embj201694561-bib-0017] Cools T , De Veylder L (2009) DNA stress checkpoint control and plant development. Curr Opin Plant Biol 12: 23–28 19010080 10.1016/j.pbi.2008.09.012

[embj201694561-bib-0018] Cools T , Iantcheva A , Weimer AK , Boens S , Takahashi N , Maes S , Van den Daele H , Van Isterdael G , Schnittger A , De Veylder L (2011) The *Arabidopsis thaliana* checkpoint kinase WEE1 protects against premature vascular differentiation during replication stress. Plant Cell 23: 1435–1448 21498679 10.1105/tpc.110.082768 PMC3101530

[embj201694561-bib-0019] Coschi CH , Ishak CA , Gallo D , Marshall A , Talluri S , Wang J , Cecchini MJ , Martens AL , Percy V , Welch I , Boutros PC , Brown GW , Dick FA (2014) Haploinsufficiency of an RB‐E2F1‐Condensin II complex leads to aberrant replication and aneuploidy. Cancer Discov 4: 840–853 24740996 10.1158/2159-8290.CD-14-0215

[embj201694561-bib-0020] Cruz‐Ramirez A , Diaz‐Trivino S , Blilou I , Grieneisen VA , Sozzani R , Zamioudis C , Miskolczi P , Nieuwland J , Benjamins R , Dhonukshe P , Caballero‐Perez J , Horvath B , Long Y , Mahonen AP , Zhang H , Xu J , Murray JA , Benfey PN , Bako L , Maree AF *et al* (2012) A bistable circuit involving SCARECROW‐RETINOBLASTOMA integrates cues to inform asymmetric stem cell division. Cell 150: 1002–1015 22921914 10.1016/j.cell.2012.07.017 PMC3500399

[embj201694561-bib-0021] Cruz‐Ramirez A , Diaz‐Trivino S , Wachsman G , Du Y , Arteaga‐Vazquez M , Zhang H , Benjamins R , Blilou I , Neef AB , Chandler V , Scheres B (2013) A SCARECROW‐RETINOBLASTOMA protein network controls protective quiescence in the *Arabidopsis* root stem cell organizer. PLoS Biol 11: e1001724 24302889 10.1371/journal.pbio.1001724 PMC3841101

[embj201694561-bib-0022] Culligan KM , Robertson CE , Foreman J , Doerner P , Britt AB (2006) ATR and ATM play both distinct and additive roles in response to ionizing radiation. Plant J 48: 947–961 17227549 10.1111/j.1365-313X.2006.02931.x

[embj201694561-bib-0023] Culligan KM , Britt AB (2008) Both ATM and ATR promote the efficient and accurate processing of programmed meiotic double‐strand breaks. Plant J 55: 629–638 18435824 10.1111/j.1365-313X.2008.03530.x

[embj201694561-bib-0024] De Schutter K , Joubes J , Cools T , Verkest A , Corellou F , Babiychuk E , Van Der Schueren E , Beeckman T , Kushnir S , Inze D , De Veylder L (2007) *Arabidopsis* WEE1 kinase controls cell cycle arrest in response to activation of the DNA integrity checkpoint. Plant Cell 19: 211–225 17209125 10.1105/tpc.106.045047 PMC1820959

[embj201694561-bib-0025] De Veylder L , Beeckman T , Beemster GT , de Almeida Engler J , Ormenese S , Maes S , Naudts M , Van Der Schueren E , Jacqmard A , Engler G , Inze D (2002) Control of proliferation, endoreduplication and differentiation by the *Arabidopsis* E2Fa‐DPa transcription factor. EMBO J 21: 1360–1368 11889041 10.1093/emboj/21.6.1360 PMC125359

[embj201694561-bib-0026] Dewitte W , Riou‐Khamlichi C , Scofield S , Healy JM , Jacqmard A , Kilby NJ , Murray JA (2003) Altered cell cycle distribution, hyperplasia, and inhibited differentiation in *Arabidopsis* caused by the D‐type cyclin CYCD3. Plant Cell 15: 79–92 12509523 10.1105/tpc.004838 PMC143452

[embj201694561-bib-0027] Dewitte W , Scofield S , Alcasabas AA , Maughan SC , Menges M , Braun N , Collins C , Nieuwland J , Prinsen E , Sundaresan V , Murray JA (2007) *Arabidopsis* CYCD3 D‐type cyclins link cell proliferation and endocycles and are rate‐limiting for cytokinin responses. Proc Natl Acad Sci USA 104: 14537–14542 17726100 10.1073/pnas.0704166104 PMC1964848

[embj201694561-bib-0028] Dick FA , Rubin SM (2013) Molecular mechanisms underlying RB protein function. Nat Rev Mol Cell Biol 14: 297–306 23594950 10.1038/nrm3567 PMC4754300

[embj201694561-bib-0029] Dissmeyer N , Weimer AK , Pusch S , De Schutter K , Alvim Kamei CL , Nowack MK , Novak B , Duan GL , Zhu YG , De Veylder L , Schnittger A (2009) Control of cell proliferation, organ growth, and DNA damage response operate independently of dephosphorylation of the *Arabidopsis* Cdk1 homolog CDKA;1. Plant Cell 21: 3641–3654 19948791 10.1105/tpc.109.070417 PMC2798325

[embj201694561-bib-0030] Flynn RL , Zou L (2011) ATR: a master conductor of cellular responses to DNA replication stress. Trends Biochem Sci 36: 133–140 20947357 10.1016/j.tibs.2010.09.005 PMC3024454

[embj201694561-bib-0031] Friesner JD , Liu B , Culligan K , Britt AB (2005) Ionizing radiation‐dependent gamma‐H2AX focus formation requires ataxia telangiectasia mutated and ataxia telangiectasia mutated and Rad3‐related. Mol Biol Cell 16: 2566–2576 15772150 10.1091/mbc.E04-10-0890 PMC1087258

[embj201694561-bib-0032] Fulcher N , Sablowski R (2009) Hypersensitivity to DNA damage in plant stem cell niches. Proc Natl Acad Sci USA 106: 20984–20988 19933334 10.1073/pnas.0909218106 PMC2791609

[embj201694561-bib-0033] Furukawa T , Curtis MJ , Tominey CM , Duong YH , Wilcox BW , Aggoune D , Hays JB , Britt AB (2010) A shared DNA‐damage‐response pathway for induction of stem‐cell death by UVB and by gamma irradiation. DNA Repair 9: 940–948 20634150 10.1016/j.dnarep.2010.06.006

[embj201694561-bib-0034] Gehl C , Waadt R , Kudla J , Mendel RR , Hansch R (2009) New GATEWAY vectors for high throughput analyses of protein‐protein interactions by bimolecular fluorescence complementation. Mol Plant 2: 1051–1058 19825679 10.1093/mp/ssp040

[embj201694561-bib-0035] Gutzat R , Borghi L , Gruissem W (2012) Emerging roles of RETINOBLASTOMA‐RELATED proteins in evolution and plant development. Trends Plant Sci 17: 139–148 22240181 10.1016/j.tplants.2011.12.001

[embj201694561-bib-0036] Harashima H , Dissmeyer N , Schnittger A (2013) Cell cycle control across the eukaryotic kingdom. Trends Cell Biol 23: 345–356 23566594 10.1016/j.tcb.2013.03.002

[embj201694561-bib-0037] Harashima H , Sugimoto K (2016) Integration of developmental and environmental signals into cell proliferation and differentiation through RETINOBLASTOMA‐RELATED 1. Curr Opin Plant Biol 29: 95–103 26799131 10.1016/j.pbi.2015.12.003

[embj201694561-bib-0038] Harper JW , Elledge SJ (2007) The DNA damage response: ten years after. Mol Cell 28: 739–745 18082599 10.1016/j.molcel.2007.11.015

[embj201694561-bib-0039] Henriques R , Magyar Z , Monardes A , Khan S , Zalejski C , Orellana J , Szabados L , de la Torre C , Koncz C , Bogre L (2010) *Arabidopsis* S6 kinase mutants display chromosome instability and altered RBR1‐E2F pathway activity. EMBO J 29: 2979–2993 20683442 10.1038/emboj.2010.164 PMC2944053

[embj201694561-bib-0040] Hinds PW (2014) A little pRB can lead to big problems. Cancer Discov 4: 764–765 25002614 10.1158/2159-8290.CD-14-0518

[embj201694561-bib-0041] Hoeijmakers JH (2009) DNA damage, aging, and cancer. N Engl J Med 361: 1475–1485 19812404 10.1056/NEJMra0804615

[embj201694561-bib-0042] Horvath BM , Magyar Z , Zhang Y , Hamburger AW , Bako L , Visser RG , Bachem CW , Bogre L (2006) EBP1 regulates organ size through cell growth and proliferation in plants. EMBO J 25: 4909–4920 17024182 10.1038/sj.emboj.7601362 PMC1618091

[embj201694561-bib-0043] Hu Z , Cools T , De Veylder L (2016) Mechanisms used by plants to cope with DNA damage. Annu Rev Plant Biol 67: 439–462 26653616 10.1146/annurev-arplant-043015-111902

[embj201694561-bib-0044] Ianari A , Natale T , Calo E , Ferretti E , Alesse E , Screpanti I , Haigis K , Gulino A , Lees JA (2009) Proapoptotic function of the retinoblastoma tumor suppressor protein. Cancer Cell 15: 184–194 19249677 10.1016/j.ccr.2009.01.026 PMC2880703

[embj201694561-bib-0045] Johnston AJ , Kirioukhova O , Barrell PJ , Rutten T , Moore JM , Baskar R , Grossniklaus U , Gruissem W (2010) Dosage‐sensitive function of retinoblastoma related and convergent epigenetic control are required during the *Arabidopsis* life cycle. PLoS Genet 6: e1000988 20585548 10.1371/journal.pgen.1000988 PMC2887464

[embj201694561-bib-0046] Kobayashi K , Suzuki T , Iwata E , Nakamichi N , Suzuki T , Chen P , Ohtani M , Ishida T , Hosoya H , Muller S , Leviczky T , Pettko‐Szandtner A , Darula Z , Iwamoto A , Nomoto M , Tada Y , Higashiyama T , Demura T , Doonan JH , Hauser MT *et al* (2015) Transcriptional repression by MYB3R proteins regulates plant organ growth. EMBO J 34: 1992–2007 26069325 10.15252/embj.201490899 PMC4551348

[embj201694561-bib-0047] Kuwabara A , Gruissem W (2014) *Arabidopsis* RETINOBLASTOMA‐RELATED and Polycomb group proteins: cooperation during plant cell differentiation and development. J Exp Bot 65: 2667–2676 24638900 10.1093/jxb/eru069

[embj201694561-bib-0048] Lang J , Smetana O , Sanchez‐Calderon L , Lincker F , Genestier J , Schmit AC , Houlne G , Chaboute ME (2012) Plant gammaH2AX foci are required for proper DNA DSB repair responses and colocalize with E2F factors. New Phytol 194: 353–363 22339405 10.1111/j.1469-8137.2012.04062.x

[embj201694561-bib-0049] Liu L , Zhang Y , Tang S , Zhao Q , Zhang Z , Zhang H , Dong L , Guo H , Xie Q (2010) An efficient system to detect protein ubiquitination by agroinfiltration in *Nicotiana benthamiana* . Plant J 61: 893–903 20015064 10.1111/j.1365-313X.2009.04109.x

[embj201694561-bib-0050] Magyar Z , De Veylder L , Atanassova A , Bako L , Inze D , Bogre L (2005) The role of the *Arabidopsis* E2FB transcription factor in regulating auxin‐dependent cell division. Plant Cell 17: 2527–2541 16055635 10.1105/tpc.105.033761 PMC1197432

[embj201694561-bib-0051] Magyar Z , Horvath B , Khan S , Mohammed B , Henriques R , De Veylder L , Bako L , Scheres B , Bogre L (2012) *Arabidopsis* E2FA stimulates proliferation and endocycle separately through RBR‐bound and RBR‐free complexes. EMBO J 31: 1480–1493 22307083 10.1038/emboj.2012.13 PMC3321179

[embj201694561-bib-0052] Nagy SK , Darula Z , Kallai BM , Bogre L , Banhegyi G , Medzihradszky KF , Horvath GV , Meszaros T (2015) Activation of AtMPK9 through autophosphorylation that makes it independent of the canonical MAPK cascades. Biochem J 467: 167–175 25646663 10.1042/BJ20141176

[embj201694561-bib-0053] Naouar N , Vandepoele K , Lammens T , Casneuf T , Zeller G , van Hummelen P , Weigel D , Ratsch G , Inze D , Kuiper M , De Veylder L , Vuylsteke M (2009) Quantitative RNA expression analysis with Affymetrix Tiling 1.0R arrays identifies new E2F target genes. Plant J 57: 184–194 18764924 10.1111/j.1365-313X.2008.03662.x

[embj201694561-bib-0054] Narasimha AM , Kaulich M , Shapiro GS , Choi YJ , Sicinski P , Dowdy SF (2014) Cyclin D activates the Rb tumor suppressor by mono‐phosphorylation. Elife 3: e02872 10.7554/eLife.02872 PMC4076869 24876129

[embj201694561-bib-0055] Nowack MK , Harashima H , Dissmeyer N , Zhao X , Bouyer D , Weimer AK , De Winter F , Yang F , Schnittger A (2012) Genetic framework of cyclin‐dependent kinase function in *Arabidopsis* . Dev Cell 22: 1030–1040 22595674 10.1016/j.devcel.2012.02.015

[embj201694561-bib-0056] Polyn S , Willems A , De Veylder L (2015) Cell cycle entry, maintenance, and exit during plant development. Curr Opin Plant Biol 23: 1–7 25449720 10.1016/j.pbi.2014.09.012

[embj201694561-bib-0057] Preuss SB , Britt AB (2003) A DNA‐damage‐induced cell cycle checkpoint in *Arabidopsis* . Genetics 164: 323–334 12750343 10.1093/genetics/164.1.323 PMC1462560

[embj201694561-bib-0058] Reidt W , Wurz R , Wanieck K , Chu HH , Puchta H (2006) A homologue of the breast cancer‐associated gene BARD1 is involved in DNA repair in plants. EMBO J 25: 4326–4337 16957774 10.1038/sj.emboj.7601313 PMC1570427

[embj201694561-bib-0059] Ricaud L , Proux C , Renou JP , Pichon O , Fochesato S , Ortet P , Montane MH (2007) ATM‐mediated transcriptional and developmental responses to gamma‐rays in *Arabidopsis* . PLoS ONE 2: e430 17487278 10.1371/journal.pone.0000430 PMC1855986

[embj201694561-bib-0060] Riou‐Khamlichi C , Huntley R , Jacqmard A , Murray JA (1999) Cytokinin activation of *Arabidopsis* cell division through a D‐type cyclin. Science 283: 1541–1544 10066178 10.1126/science.283.5407.1541

[embj201694561-bib-0061] Rosen EM (2013) BRCA1 in the DNA damage response and at telomeres. Front Genet 4: 85 23802008 10.3389/fgene.2013.00085 PMC3689208

[embj201694561-bib-0062] Sadasivam S , DeCaprio JA (2013) The DREAM complex: master coordinator of cell cycle‐dependent gene expression. Nat Rev Cancer 13: 585–595 23842645 10.1038/nrc3556 PMC3986830

[embj201694561-bib-0063] Sakamoto T , Inui YT , Uraguchi S , Yoshizumi T , Matsunaga S , Mastui M , Umeda M , Fukui K , Fujiwara T (2011) Condensin II alleviates DNA damage and is essential for tolerance of boron overload stress in *Arabidopsis* . Plant Cell 23: 3533–3546 21917552 10.1105/tpc.111.086314 PMC3203421

[embj201694561-bib-0064] Schepers A , Ritzi M , Bousset K , Kremmer E , Yates JL , Harwood J , Diffley JF , Hammerschmidt W (2001) Human origin recognition complex binds to the region of the latent origin of DNA replication of Epstein‐Barr virus. EMBO J 20: 4588–4602 11500385 10.1093/emboj/20.16.4588 PMC125560

[embj201694561-bib-0065] Scheres B (2007) Stem‐cell niches: nursery rhymes across kingdoms. Nat Rev Mol Cell Biol 8: 345–354 17450175 10.1038/nrm2164

[embj201694561-bib-0066] Sherman MH , Bassing CH , Teitell MA (2011) Regulation of cell differentiation by the DNA damage response. Trends Cell Biol 21: 312–319 21354798 10.1016/j.tcb.2011.01.004 PMC3089693

[embj201694561-bib-0067] Shiloh Y , Ziv Y (2013) The ATM protein kinase: regulating the cellular response to genotoxic stress, and more. Nat Rev Mol Cell Biol 14: 197–210 23847781

[embj201694561-bib-0068] Shiloh Y (2014) ATM: expanding roles as a chief guardian of genome stability. Exp Cell Res 329: 154–161 25218947 10.1016/j.yexcr.2014.09.002

[embj201694561-bib-0069] Su TT (2006) Cellular responses to DNA damage: one signal, multiple choices. Annu Rev Genet 40: 187–208 16805666 10.1146/annurev.genet.40.110405.090428

[embj201694561-bib-0070] Trapp O , Seeliger K , Puchta H (2011) Homologs of breast cancer genes in plants. Front Plant Sci 2: 19 22629260 10.3389/fpls.2011.00019 PMC3355568

[embj201694561-bib-0071] Vanstraelen M , Baloban M , Da Ines O , Cultrone A , Lammens T , Boudolf V , Brown SC , De Veylder L , Mergaert P , Kondorosi E (2009) APC/C‐CCS52A complexes control meristem maintenance in the *Arabidopsis* root. Proc Natl Acad Sci USA 106: 11806–11811 19553203 10.1073/pnas.0901193106 PMC2710644

[embj201694561-bib-0072] Velez‐Cruz R , Manickavinayaham S , Biswas AK , Clary RW , Premkumar T , Cole F , Johnson DG (2016) RB localizes to DNA double‐strand breaks and promotes DNA end resection and homologous recombination through the recruitment of BRG1. Genes Dev 30: 2500–2512 27940962 10.1101/gad.288282.116 PMC5159665

[embj201694561-bib-0073] Waterworth WM , Drury GE , Bray CM , West CE (2011) Repairing breaks in the plant genome: the importance of keeping it together. New Phytol 192: 805–822 21988671 10.1111/j.1469-8137.2011.03926.x

[embj201694561-bib-0074] Welch D , Hassan H , Blilou I , Immink R , Heidstra R , Scheres B (2007) *Arabidopsis* JACKDAW and MAGPIE zinc finger proteins delimit asymmetric cell division and stabilize tissue boundaries by restricting SHORT‐ROOT action. Genes Dev 21: 2196–2204 17785527 10.1101/gad.440307 PMC1950858

[embj201694561-bib-0075] Wildwater M , Campilho A , Perez‐Perez JM , Heidstra R , Blilou I , Korthout H , Chatterjee J , Mariconti L , Gruissem W , Scheres B (2005) The RETINOBLASTOMA‐RELATED gene regulates stem cell maintenance in *Arabidopsis* roots. Cell 123: 1337–1349 16377572 10.1016/j.cell.2005.09.042

[embj201694561-bib-0076] Xiao H , Goodrich DW (2005) The retinoblastoma tumor suppressor protein is required for efficient processing and repair of trapped topoisomerase II‐DNA‐cleavable complexes. Oncogene 24: 8105–8113 16091739 10.1038/sj.onc.1208958 PMC2799250

[embj201694561-bib-0077] Xiong Y , McCormack M , Li L , Hall Q , Xiang C , Sheen J (2013) Glucose‐TOR signalling reprograms the transcriptome and activates meristems. Nature 496: 181–186 23542588 10.1038/nature12030 PMC4140196

[embj201694561-bib-0078] Yi D , Alvim Kamei CL , Cools T , Vanderauwera S , Takahashi N , Okushima Y , Eekhout T , Yoshiyama KO , Larkin J , Van den Daele H , Conklin P , Britt A , Umeda M , De Veylder L (2014) The *Arabidopsis* SIAMESE‐RELATED cyclin‐dependent kinase inhibitors SMR5 and SMR7 regulate the DNA damage checkpoint in response to reactive oxygen species. Plant Cell 26: 296–309 24399300 10.1105/tpc.113.118943 PMC3963576

[embj201694561-bib-0079] Yoshiyama K , Conklin PA , Huefner ND , Britt AB (2009) Suppressor of gamma response 1 (SOG1) encodes a putative transcription factor governing multiple responses to DNA damage. Proc Natl Acad Sci USA 106: 12843–12848 19549833 10.1073/pnas.0810304106 PMC2722309

[embj201694561-bib-0080] Yoshiyama KO , Kobayashi J , Ogita N , Ueda M , Kimura S , Maki H , Umeda M (2013a) ATM‐mediated phosphorylation of SOG1 is essential for the DNA damage response in *Arabidopsis* . EMBO Rep 14: 817–822 23907539 10.1038/embor.2013.112 PMC3790055

[embj201694561-bib-0081] Yoshiyama KO , Sakaguchi K , Kimura S (2013b) DNA damage response in plants: conserved and variable response compared to animals. Biology 2: 1338–1356 24833228 10.3390/biology2041338 PMC4009792

[embj201694561-bib-0082] Zalmas LP , Coutts AS , Helleday T , La Thangue NB (2013) E2F‐7 couples DNA damage‐dependent transcription with the DNA repair process. Cell Cycle 12: 3037–3051 23974101 10.4161/cc.26078 PMC3875678

[embj201694561-bib-0083] Zebell SG , Dong X (2015) Cell‐cycle regulators and cell death in immunity. Cell Host Microbe 18: 402–407 26468745 10.1016/j.chom.2015.10.001 PMC4609028

