# Supplementary material for: Arabidopsis RETINOBLASTOMA RELATED directly regulates DNA damage responses through functions beyond cell cycle control
Source: EMBO J. 2017 Mar 20;36(9):1261–78. doi: 10.15252/embj.201694561 (PMC5412863; doi:10.15252/embj.201694561)
Supplement: Supplementary file 1 — Appendix [file EMBJ-36-1261-s001.pdf]

APPENDIX

Table of Contents

Appendix Supplementary Methods

Appendix Supplementary References

Appendix Figure S1

Appendix Figure S2

Appendix Table S1

Appendix Table S2

Appendix Table S3

Appendix Table S4

Appendix Table S5

Appendix Supplementary Methods

Immuno-labelling and fluorescence microscopy

Primary and secondary antibodies used for immune-fluorescence labelling were diluted as follows: anti-γ-H2AX (Friesner *et al*, 2005, Amiard *et al*, 2010, kindly provided by Ch. I. White, Clermont Université, France); 1:600 for Alexa Fluor 488 and 1:1000 for Alexa Fluor 594 secondary antibody, anti-RBR1 (Agrisera) 1:7000, mouse and rabbit anti-GFP (Abcam) 1:250, 1:3000, respectively and anti-CenH3 (Abcam) 1:800. Alexa Fluor 488, Alexa Fluor 594, Alexa Fluor 647- conjugated anti-mouse, anti-rabbit and anti-chicken antibodies (Jackson ImmunoResearch Laboratories) were diluted 1:600, 1:800, 1:700, respectively. Chromatin was stained by DAPI.

For fluorescence microscopy Olympus IX-81 FV-1000 confocal imaging system was used with oil immersion objective 100x/1.45, and dry objective 40x/0.95 was used; DAPI excitation (ex) was 405 nm, and emission (em) was at 425-460 nm, Alexa488 ex 473 nm, em 485-545 nm; Alexa 594 ex 559 nm, em 575-640 nm; Alexa 647 ex 635 nm, em 655-755 nm. Laser scanning was performed using the sequential multi-track mode to avoid bleed-through. Chromatic shift and aberration of the optical system was determined and corrected with FV10 ASW2.0 (Olympus, Tokyo, Japan) software using TetraSpeck 0.21 µm beads

(Invitrogen) as fiducial markers. Images were analysed by FV10 ASW2.0 and prepared in Adobe Photoshop CS4 and Adobe Illustrator CS4. Counting of immune-labelled nuclei and foci was performed in Adobe Photoshop CS6 extended using objects counting functions.

EdU staining

5-Ethynyl-2´-deoxyuridine (EdU) labelling was performed in whole mount preparation of root tips. EdU pulse was applied in dilution of 1:1000 and seedlings incubated in dark for 1h. Seedlings were fixed in 3.7 % formaldehyde in MTSB, pH 6.9 for 1h. Samples were then washed in MTSB, treated with 0.5 % Triton in PBS for 15 min, washed and incubated for 40 min in Click-IT reaction mixture (Molecular Probes, Eugene, OR, USA).

Generating functional AtBRCA1 constructs

The full-length genomic fragment (from ATG to stop codon, 4457bp) was used to generate the GFP and 10xmyc markers labelled AtBRCA1 protein. To express the AtBRCA1-GFP protein the promoter region (-383 to -1) was used, while for overexpression, the GVX1090 promoter. The At*brca1-1* mutant was used as a genetic background for transformation. Several independent transformants were recovered and analysed functionally. The AtBRCA1-GFP construct was detectable via confocal microscopy after MMC induction, while Western blot analysis was carried out to study the presence of the AtBRCA1-10xmyc protein after β-estradiol induction (24 hrs, 5 µM β–estradiol, Appendix Fig S1).

Interaction of *in vitro* translated proteins

To test whether AtBRCA1 and RBR proteins interact, we translated them *in vitro* in wheat germ extract and used the translated E2FA and E2FB as positive controls. RBR was tagged N-terminally with biotin, while AtBRCA1, E2FA and E2FB were tagged with Glutathione-S-transferase (GST) at their N-termini. We performed co-immuno precipitations using streptavidin labelled magnetic beads and visualised proteins by Western blot using ExtrAvidin-POD (EA-POD) tagged RBR and GST antibody. RBR showed a clear and specific

interaction with AtBRCA1, compared to the GST control, but was weaker than the positive controls of E2FA (Fig 5). The strength of RBR/E2FB was similar to RBR/E2FA.

Isolation of root material for transcriptome analysis

In order to follow the dynamics underlying the phenotypic changes due to *RBR* silencing we have analysed the *rRbr* line at early time points of development. At 4 days after sowing (das) the organization of the stem cell niche in *rRBr* largely resembles the one in the wild-type control visualized by Lugol staining using differential interference contrast (DIC) microscopy (Fig EV1A). Labelling the cells with EdU (5-ethynyl-2'- deoxyuridine, 6h), however, we could show excess number of columella (CSC) and lateral root cap initials (LRC) in S-phase (Fig EV1B). The number of stem cell layers and the number of cells going through S-phase continued to increase at 6 and 10 das (Fig EV1A and B, respectively). The cell death inducing effect of *RBR* silencing was also detectable from 4 das onwards and the number of dead cells increased by time (Fig EV1C). Germination of seeds, Col-0 and *rRBr*, for the different time points started at the same time under the same conditions and were repeated three times. The phenotypic changes were followed in each experiment via Lugol staining. As the changes occur mainly in the stem cell niche we have dissected the root tips carefully under the microscope and collected material between 20-40mg. RNA isolation and the quality control were carried out according to the manufacture's recommendation (RNA cc. varied, 3-30 µg). The level of *RBR* silencing was determined by qRT-PCR from the 10das sample of each repeat and found to be around 80% of the control at the same time point.

Analysis of micro-array data

cDNA synthesis, labelling and hybridization to ATH1 Affymetrix Chips were performed at ServiceXS (Leiden, The Netherlands). At least, two biological replicas were used for each time point.

Microarray analysis was performed using the Affymetrix package within Bioconductor (www.bioconductor.org). Samples were normalized with the RMA algorithm and differential expression was assessed using the LIMMA package (Smyth, 2004) and the Benjamini and

Hochberg multiple testing correction (Benjamini *et al.*, 2001). In order to identify the common *rRBr* targets through the analysed time frame, samples were clustered in two groups (wild-type and *rRBr*). We defined differentially expressed genes when p-value < 0.01 was combined with a fold change ≥ 1.4.

The level of *RBR* reduction in the microarray samples matched well with the reduction measured by qRT-PCR in the dissected *rBRr* root tips compared to Col-0. No reduction was detectable when RNA was isolated from the entire meristem, confirming that our sample was enriched for cells within the *RCH1* expression domain where *RBR* silencing had taken place.

Gene ontology (GO) overrepresentation analysis was carried out using Fisher Exact Test with FDR correction (p<0.01) and background population Tair10 ATH1 at Virtual Plant 1.3 (Katari *et al.*, 2010). Co-expression networks were obtained from ATTED-II web server (Obayashi *et al.*, 2011).

Analysis of the differentially expressed genes

In total, 99 genes showed significant differential expression between *rRBr* and Col-0 during the studied 3 time points, using the above stringent parameters in statistical data analysis (AppendixTable S1). Most of the genes (82) were up-, and 17 were down-regulated, including *RBR* itself. Gene ontology (GO) analysis uncovered that the up-regulated genes within the *rRBr* set were significantly enriched for nuclear proteins and functionally related to the following major processes; (1) nucleosome and chromosome assembly and organisation (39%), (2) DNA replication (21%) and (3) DNA damage response and DNA repair (16%) (4) cell cycle checkpoint (6%), (Appendix Table S2). To reveal possible links among the genes in the three GO categories, we performed a co-expressional analysis, seeded around a well-characterized member of each cluster; the *HISTONE 2B* (*HTB9,* At3g45980, cluster 1, Appendix Fig S2A), the *RIBONUCLEOTIDE REDUCTASE* (*RNR*) *SMALL SUBUNIT* (*TSO2,* At3g27060, cluster 2, Appendix Fig S2B) and the *BREAST CANCER SUSCEPTIBILITY1* (*AtBRCA1,* At4g21070, cluster 3 Appendix Fig S2C). A large portion (11/27) of the *HISTONE* genes, mainly HISTONE2-type, showed co-expression (Appendix Fig S2A). The replication

related transcripts, the *PROLIFERATING CELL NUCLEAR ANTIGEN 2 (PCNA2)*, the *REPLICON PROTEIN A2* (*RPA2*), alongside some non-annotated transcripts, formed a co-expressional cluster. The *PROLIFERA*, the *STRUCTURAL MAINTENANCE OF CHROMOSOME 6A*, the *DNA POLYMERASE ALPHA* and its *CATALYTIC SUBUNIT* did not fall in this co-expressional cluster but their expression was also consistent with the regulatory function of RBR at the entry of replication (Appendix Fig S2B). Similarly, a large overlap (10 out of 14 transcripts) was found between the *AtBRCA1* co-expressional cluster and the *rRBr* gene set annotated as DNA damage response (DDR), among others the *RECOMBINASE PROTEIN51* (*RAD51*), *POLY(ADP-RIBOSE) POLYMERASE2* (*PARP2*), *GAMMA-IRRADIATION AND MITOMYCIN INDUCED 1* (*GMI1*) (Appendix Fig S2C). Genes involved in cell cycle checkpoint activation, such as the *CYCLIN DEPENDENT KINASE INHIBITORS*, the *KIP-RELATED PROTEIN3* and *5* (*KRP3* and *KRP5*) and *SIAMESE-RELATED4* (*SMR4*) and the related *SMR6*, were also up-regulated. At lower level of statistical stringency, another family member, *SMR5* also showed increased expression in *rRBr*. A large portion (58/82) of the up-regulated genes overlapped with the published dataset of inducible RBR silencing in leaves (Borghi *et al.*, 2010), including all the replication-related genes and part of the AtBRCA1 co-expressional genes (Appendix Table S1).

To relate the *rRBr* transcriptome to upstream and downstream regulatory networks of RBR and to the DNA damage response pathways, we performed a meta-analysis of micro-array experiments. We found a substantial overlap with our *rRBr* and the *CYCD3;1* overexpression (de Jager *et al.*, 2009, Menges *et al.*, 2006) or the *E2Fa-DPa* co-overexpression datasets (de Jager *et al.*, 2009, Menges *et al.*, 2006, Naouar *et al.*, 2009, Vandepoele *et al.*, 2005), mainly representing transcripts confined to the co-expressional clusters described above (Appendix Table S1). Eight out of the ten “BRCA1 co-expressional genes” are expressed in an ATM- and SOG1-dependent manner after gamma-irradiation (Appendix Table S1) and induced by genotoxic agents, such as hydroxyurea (Adachi *et al.*, 2011, Cools *et al.*, 2011) or bleomycin (Yi *et al.*, 2014). The same transcripts are also among

the CYCD3.1 regulated genes, suggesting that these transcripts are commonly regulated by the CYCD3;1-RBR and ATM-SOG1 pathways. In addition, some RBR regulated transcripts are ATM dependent, but independent of SOG1 and CYCD3;1 regulation, raising the possibility of a SOG1-independent pathway.

Expression analysis

RNA was extracted from 5-6 das seedlings or root samples (varied between 50-100) using the RNeasy Mini Kit (Qiagen). cDNA was synthesized with the QuantiTect Reverse Transcription Kit according to the manufacturer's recommendation (http://qiagen.com). Quantitative Real-Time PCR (qRT-PCR) was carried out using SYBR Green Jumpstart reaction mixture (Sigma) on 0,2 µg cDNA. Transcript levels were normalized to *ACTIN2* (*ACT*) level and analysed using Relative Expression Software Tool 2009 (REST 2009, Qiagen). Primer sequences are summarised in Appendix Table S3. Each treatment and mutant analysis was repeated at least twice (biological repeat, n) in different laboratory; the effect of genotoxic agent was controlled by confocal microscopy prior to RNA isolation.

Mutants and their phenotypic analysis

The Arabidopsis thaliana ecotype Landsberg erecta (*35S::CYCD3;1*) line G54 (Dewitte *et al.*, 2003, Riou-Khamlichi *et al.*, 1999) was introgressed to Col-0 to generate Col-0 (*CYCD3;1OE*). F3 batches which were homozygous for the T-DNA insertion and displayed Col-0 inflorescences were identified. PCR-based genotyping was carried out using primers listed in Appendix Table S3.

The influenza HA-tagged E2FB had been previously cloned into the pK7WG2 Gateway vector (Magyar *et al.*, 2005). Transgenic Arabidopsis plants over-expressing the dimerization partner A (DPA; (De Veylder *et al.*, 2002) were transformed with the HA-E2FB constructusing the flower-dip method. Thirteen transgenic T1 lines expressing both transgenes were identified, and a single T-DNA insertion line (line 10/15) for both transgenes was selected and used in this study.

The position of the different *e2fa* mutations is shown in Fig EV5A . The *e2fa-1*, *e2fa-2* mutants (MPIZ_244, GABI-348E09, respectively) were characterized earlier (Berckmans *et al.*, 2011); using a primer combination downstream of the relevant insertions, no mRNA was detected in either of the mutants, hence they did not produce the full-sized E2FA. However, the authors did not exclude that truncated protein could be synthesized. In our preliminary experiments, using a specific E2FA N-terminal-related antibody, indeed we could detect a specific, lower mobility protein in the *e2fa-2* mutant. Based on this, we assumed that *e2fa-1* and *e2fa-2* are loss-of-function rather than null mutants. The truncated *e2fa-1* and *e2fa-2* alleles differ in their 'marked-box' domain but both could dimerize with DPs. Thus, we suggested that the difference in the observed phenotype did not relate to a dominant negative effect sequestering DPs, but rather, the "marked-box' domain plays an important role. The *e2fa-3* mutant was isolated by (Xiong *et al.,* 2013) and described as a null allele. The introgressed *amiRBR;e2fa* mutants were genotyped for the *e2fa* mutations, while RBR silencing was analysed for via qRT-PCR (Fig EV5B).

The presence of *amiRBR* construct in the *amiRBR;brca1* mutants was first pre-screened for the presence of a GFP marker introgressed together with the *amiRBR* construct. The GFP marker was not a direct "readout" for the reduction of the *RBR* level, thus the same (individual) seedlings analysed for cell death were also tested for QC and stem cell maintenance; in addition, we counted the columella cell layers as an indication for extra stem cell division (Fig EV4F and G). EdU labelling on the homozygous F3 batch also confirmed that the *$35S_{pro}$:amiGORBR* (Cruz-Ramirez *et al.*, 2013) transgene was still functional (Fig EV4D). The double mutants were genotyped, and the absence of functional AtBRCA1 was controlled by expressional studies upon MMC treatment (Fig EV5C).

To follow whether spontaneous cell death upon RBR silencing depends on the SOG1 function, we transformed homozygous *sog1-1* plants (Preuss & Britt, 2003) with the *$35S_{pro}$:amiGORBR* construct (Cruz-Ramirez *et al.*, 2013). More than 20 independent transformants were generated, genotyped by sequencing of the *sog1-1* locus and analysed

for *RBR* silencing (Fig EV6C).

Cell death phenotype was quantified by scoring the number of dead cells in the QC, columella and lateral root cap stem and daughter cells. Parallel, we quantified the area of dead cells also in the proximal meristem. As this assay was very sensitive and was dependent on the age of the seedlings (5 to 6 days), the length of storage of the MMC, the affected area especially, in the case of the *Atbrca1* mutants differed from experiment to experiment. For this reason, cell death was quantified in each experiment from each line and the control at least on 15 seedlings. Though the relative values between different experiments varied, the ratio of cell death area comparing the control and mutants was nearly constant (Fig EV3E).

Protein complex isolation, LC-MS/MS identification and label free MS quantitation

Sample preparation

E2FA-GFP *(pE2FA:gE2FA-GFP)* and *p35:GFP* (Magyar *et al.*, 2012) seeds were germinated in normal growth conditions. For genotoxic treatment 6 das seedlings were transferred to liquid MS media with or without MMC (20 µg/ml) for 16 hours. 150-200 seedlings were harvested and processed. Total proteins were extracted as described earlier (Henriques *et al.*, 2010). The total protein extracts (4mg/IP) were immune-purified using anti-GFP antibody coupled with very small magnetic beads (MACS® Technology, Miltenyi) digested in column with trypsin, and analysed in a single run on the mass spectrometer (Hubner *et al.*, 2010).

Mass spectrometry

The resulting peptide mixture first was desalted (Omix C18 100 ul tips, Varian) then analysed by LC-MS/MS using a nanoflow RP-HPLC (Lc program: linear gradient of 3-40 % B in 100 min, solvent A: 0.1% formic acid in water, solvent B: 0.1% formic acid in acetonitrile) on-line coupled to a linear ion trap-Orbitrap (Orbitrap-Elite, Thermo Fisher Scientific) mass spectrometer operating in positive ion mode. Data acquisition was carried out in data-dependent fashion; the 10 most abundant, multiply charged ions were selected from each

MS survey for MS/MS analysis (MS spectra were acquired in the Orbitrap, and CID spectra in the linear ion trap).

Data interpretation

Raw data were converted into peak-lists using the in-house PAVA software (Guan *et al.*, 2011) and searched against the Swissprot database (version:16/04/2015, 548208 proteins) using the Protein Prospector search engine (v5.15.1) with the following parameters: enzyme: trypsin with maximum 1 missed cleavage; mass accuracies: 5 ppm for precursor ions and 0.6 Da for fragment ions (both monoisotopic); fixed modification: carbamidomethylation of Cys residues; variable modifications: acetylation of protein N-termini; Met oxidation; cyclization of N-terminal Gln residues allowing maximum 2 variable modifications per peptide. Acceptance criteria: minimum scores: 22 and 15; maximum E values: 0.01 and 0.05 for protein and peptide identifications, respectively. Another database search was also performed using the same search and acceptance parameters except that Uniprot.random.concat database (version:16/04/2015) was searched with Arabidopsis thaliana species restriction (52524 proteins) including additional proteins identified from the previous Swissprot search (protein score>50). False discovery rate was estimated using peptide identifications representing randomized proteins (2* #of random IDs/ total peptide IDs) = 2 times number of random IDs divided by peptide IDs.

Spectral counting was used to estimate relative abundance of individual proteins in the MMC-treated and control samples: peptide counts of the individual proteins were normalized to the total number of peptide identifications in each sample, then these normalised peptide counts were compared in the two samples. The median of these normalized peptide count ratios was 0.9715, therefore, the ratios were corrected with this value.

Appendix Supplementary References

Adachi S, Minamisawa K, Okushima Y, Inagaki S, Yoshiyama K, Kondou Y, Kaminuma E, Kawashima M, Toyoda T, Matsui M, Kurihara D, Matsunaga S, Umeda M (2011) Programmed induction of endoreduplication by DNA double-strand breaks in Arabidopsis. Proc Natl Acad Sci U S A 108: 10004-9

Benjamini Y, Drai D, Elmer G, Kafkafi N, Golani I (2001) Controlling the false discovery rate in behavior genetics research. Behav Brain Res 125: 279-84

Berckmans B, Vassileva V, Schmid SP, Maes S, Parizot B, Naramoto S, Magyar Z, Alvim Kamei CL, Koncz C, Bogre L, Persiau G, De Jaeger G, Friml J, Simon R, Beeckman T, De Veylder L (2011) Auxin-dependent cell cycle reactivation through transcriptional regulation of Arabidopsis E2Fa by lateral organ boundary proteins. Plant Cell 23: 3671-83

Borghi L, Gutzat R, Futterer J, Laizet Y, Hennig L, Gruissem W (2010) Arabidopsis RETINOBLASTOMA-RELATED is required for stem cell maintenance, cell differentiation, and lateral organ production. Plant Cell 22: 1792-811

Cools T, Iantcheva A, Weimer AK, Boens S, Takahashi N, Maes S, Van den Daele H, Van Isterdael G, Schnittger A, De Veylder L (2011) The Arabidopsis thaliana checkpoint kinase WEE1 protects against premature vascular differentiation during replication stress. Plant Cell 23: 1435-48

Cruz-Ramirez A, Diaz-Trivino S, Wachsman G, Du Y, Arteaga-Vazquez M, Zhang H, Benjamins R, Blilou I, Neef AB, Chandler V, Scheres B (2013) A SCARECROW-RETINOBLASTOMA protein network controls protective quiescence in the Arabidopsis root stem cell organizer. PLoS Biol 11: e1001724

Culligan KM, Robertson CE, Foreman J, Doerner P, Britt AB (2006) ATR and ATM play both distinct and additive roles in response to ionizing radiation. Plant J 48: 947-61

de Jager SM, Scofield S, Huntley RP, Robinson AS, den Boer BG, Murray JA (2009) Dissecting regulatory pathways of G1/S control in Arabidopsis: common and distinct targets of CYCD3;1, E2Fa and E2Fc. Plant Mol Biol 71: 345-65

De Veylder L, Beeckman T, Beemster GT, de Almeida Engler J, Ormenese S, Maes S, Naudts M, Van Der Schueren E, Jacqmard A, Engler G, Inze D (2002) Control of proliferation, endoreduplication and differentiation by the Arabidopsis E2Fa-DPa transcription factor. EMBO J 21: 1360-8

Dewitte W, Riou-Khamlichi C, Scofield S, Healy JM, Jacqmard A, Kilby NJ, Murray JA (2003) Altered cell cycle distribution, hyperplasia, and inhibited differentiation in Arabidopsis caused by the D-type cyclin CYCD3. Plant Cell 15: 79-92

Guan S, Price JC, Prusiner SB, Ghaemmaghami S, Burlingame AL (2011) A data processing pipeline for mammalian proteome dynamics studies using stable isotope metabolic labeling. Mol Cell Proteomics 10: M111 010728

Henriques R, Magyar Z, Monardes A, Khan S, Zalejski C, Orellana J, Szabados L, de la Torre C, Koncz C, Bogre L (2010) Arabidopsis S6 kinase mutants display chromosome instability and altered RBR1-E2F pathway activity. EMBO J 29: 2979-93

Hubner NC, Bird AW, Cox J, Splettstoesser B, Bandilla P, Poser I, Hyman A, Mann M (2010) Quantitative proteomics combined with BAC TransgeneOmics reveals in vivo protein interactions. J Cell Biol 189: 739-54

Katari MS, Nowicki SD, Aceituno FF, Nero D, Kelfer J, Thompson LP, Cabello JM, Davidson RS, Goldberg AP, Shasha DE, Coruzzi GM, Gutierrez RA (2010) VirtualPlant: a software platform to support systems biology research. Plant Physiol 152: 500-15

Kobayashi K, Suzuki T, Iwata E, Nakamichi N, Suzuki T, Chen P, Ohtani M, Ishida T, Hosoya H, Muller S, Leviczky T, Pettko-Szandtner A, Darula Z, Iwamoto A, Nomoto M, Tada Y, Higashiyama T, Demura T, Doonan JH, Hauser MT et al. (2015) Transcriptional repression by MYB3R proteins regulates plant organ growth. EMBO J

Magyar Z, De Veylder L, Atanassova A, Bako L, Inze D, Bogre L (2005) The role of the Arabidopsis E2FB transcription factor in regulating auxin-dependent cell division. Plant Cell 17: 2527-41

Magyar Z, Horvath B, Khan S, Mohammed B, Henriques R, De Veylder L, Bako L, Scheres B, Bogre L (2012) Arabidopsis E2FA stimulates proliferation and endocycle separately through RBR-bound and RBR-free complexes. EMBO J 31: 1480-93

Menges M, Samland AK, Planchais S, Murray JA (2006) The D-type cyclin CYCD3;1 is limiting for the G1-to-S-phase transition in Arabidopsis. Plant Cell 18: 893-906

Naouar N, Vandepoele K, Lammens T, Casneuf T, Zeller G, van Hummelen P, Weigel D, Ratsch G, Inze D, Kuiper M, De Veylder L, Vuylsteke M (2009) Quantitative RNA expression analysis with Affymetrix Tiling 1.0R arrays identifies new E2F target genes. Plant J 57: 184-94

Obayashi T, Nishida K, Kasahara K, Kinoshita K (2011) ATTED-II updates: condition-specific gene coexpression to extend coexpression analyses and applications to a broad range of flowering plants. Plant Cell Physiol 52: 213-9

Preuss SB, Britt AB (2003) A DNA-damage-induced cell cycle checkpoint in Arabidopsis. Genetics 164: 323-34

Riou-Khamlichi C, Huntley R, Jacqmard A, Murray JA (1999) Cytokinin activation of Arabidopsis cell division through a D-type cyclin. Science 283: 1541-4

Smyth GK (2004) Linear models and empirical bayes methods for assessing differential expression in microarray experiments. Stat Appl Genet Mol Biol 3: Article3

Vandepoele K, Vlieghe K, Florquin K, Hennig L, Beemster GT, Gruissem W, Van de Peer Y, Inze D, De Veylder L (2005) Genome-wide identification of potential plant E2F target genes. Plant Physiol 139: 316-28

Xiong Y, McCormack M, Li L, Hall Q, Xiang C, Sheen J (2013) Glucose-TOR signalling reprograms the transcriptome and activates meristems. Nature 496: 181-6

Yi D, Alvim Kamei CL, Cools T, Vanderauwera S, Takahashi N, Okushima Y, Eekhout T, Yoshiyama KO, Larkin J, Van den Daele H, Conklin P, Britt A, Umeda M, De Veylder L (2014) The Arabidopsis SIAMESE-RELATED Cyclin-Dependent Kinase Inhibitors SMR5 and SMR7 Regulate the DNA Damage Checkpoint in Response to Reactive Oxygen Species. Plant Cell 26: 296-309

Yoshiyama K, Conklin PA, Huefner ND, Britt AB (2009) Suppressor of gamma response 1 (SOG1) encodes a putative transcription factor governing multiple responses to DNA damage. Proc Natl Acad Sci U S A 106: 12843-8

Legends to Appendix Supplementary Figures and Tables

Appendix Fig S1. Elevated level of AtBRCA1 is not sufficient to induce cell death response.

(**A)** Accumulation of AtBRCA1-10xmyc protein after 24h β-estradiol (5 μM) induction (+) compared to the non-induced control (-). * indicates lines 3, 4, 5 and 7 used for microscopical studies.

(**B**) No spontaneous cell death response was detected after 24h β-estradiol induction, arrow indicates QC position.

Appendix Fig S2. Co-expression modules of differentially expressed transcripts.

Three co-expression modules created by ATTED-II centred around (**A**) Histone HTB9 (At3g45980), (**B**) TSO2 (At3g27060) and (C) AtBRCA1 (At4g21070). Solid black edges connecting the genes indicate co-expression and brown edges refer to conserved co-expression between Arabidopsis and at least one of three mammalian species (human, mouse and rat) used for comparison. The three Histone highlighted with a black box can form a connection between the overlapping two clusters.

Appendix Table S1. Differentially expressed genes upon RBR silencing comparing the transcriptome of *rRBr* and Col-0

(**A** and **B**) Annotation and (**C**) locus of up-and downregulated transcripts. Upregulated genes are ordered according to the adjusted p-value (**F**), while downregulated genes are listed according to the level of changes (**D**).

(**D**) Fold change (FC) threshold (>1.4) determining differential expression.

(**E**) Average intensity

(**F**) Adjusted p-value for multiple testing correction using Benjamini-Hochbert approach

(**G**) Co-expressional analysis using ATTED-II centred around HTB9, At3g45980 (A); TSO2, At3g27060 (B) and At4g21070=AtBRCA1 (C). Labelling (A), (B) and (C) refers to images in Appendix Fig S2. Colour indicates GO ontology clusters blue colours refers to DNA repair (column **I**), while red colour illustrates DNA replication (columns **H**).

(**H**) GO ontology cluster; Nucleosome assembly: GO0006334

(**I**) GO ontology cluster DNA repair and GO0006281 and GO0000724, coloured blue, if overlaps with co-expressional category centred around AtBRCA1:

(**J**) GO ontology cluster: DNA dependent DNA replication, GO0006261, labelled red, if overlaps with co-expressional category centred around TSO2.

(**K**) Cell-cycle related GO clusters: GO0010389, GO0051726

Genes showing differential expression (**L**) in *sog1-1* (Yoshiyama *et al.*, 2009) and (**M**) *atm-2* (Culligan *et al.*, 2006) mutants.

(**N**) Genes harbouring a potential E2F motif (8bp), using prediction published by Naouar *et al*, 2009, Table S4) in a 1-kb promoter region.

Overlapping differentially expressed genes to (**O**) *CYCD3OE* (de Jager *et al.*, 2009), (**P**) *E2FA-DPaOE* (Naouar *et al.*, 2009) and (**Q-X**) inducible *RBR* RNAi line (Borghi et al, 2010), the induction time and values refer to the article.

Appendix Table S2. Enrichment of Gene Ontology (GO) terms of differentially expressed genes comparing the transcriptome of *rRBr* and Col-0

Appendix Table S3. List of primers used in this study

Appendix Table S4. Label free MS quantitation of E2FA interaction with RBR, DPA and DPB with and without MMC treatment
Seedlings with GFP-tagged E2FA (7das) were treated with and without MMC for 16h, GFP-pull downs were analysed by MS and E2FA, RBR, DPA, DPB were quantitated label-free.

Appendix Table S5. Interaction of E2FA and E2FB with DREAM complex components
Seedlings expressing either GFP-tagged E2FA or E2FB and GFP (7 das) were collected. Pull-downs were performed and analysed by LC-MS/MS. Identified DREAM complex components (Kobayashi *et al.*, 2015) are shown by the number of unique peptides and sequence coverage. None of the identified peptides were detected in the control expressing GFP alone. *representative result from independent experiments (n>10).

Appendix Figure S1

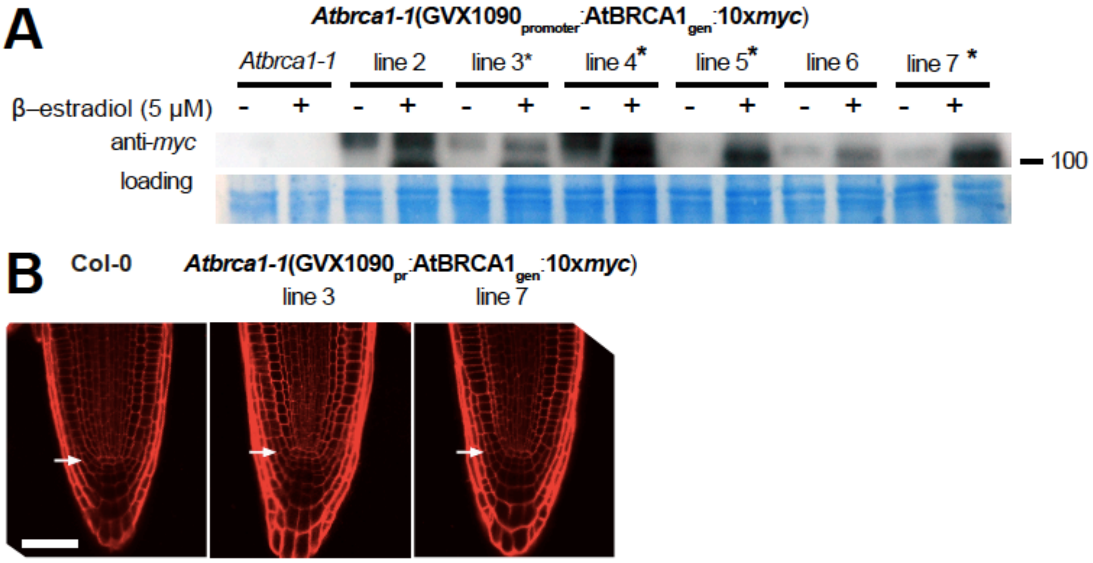

Appendix Figure S2

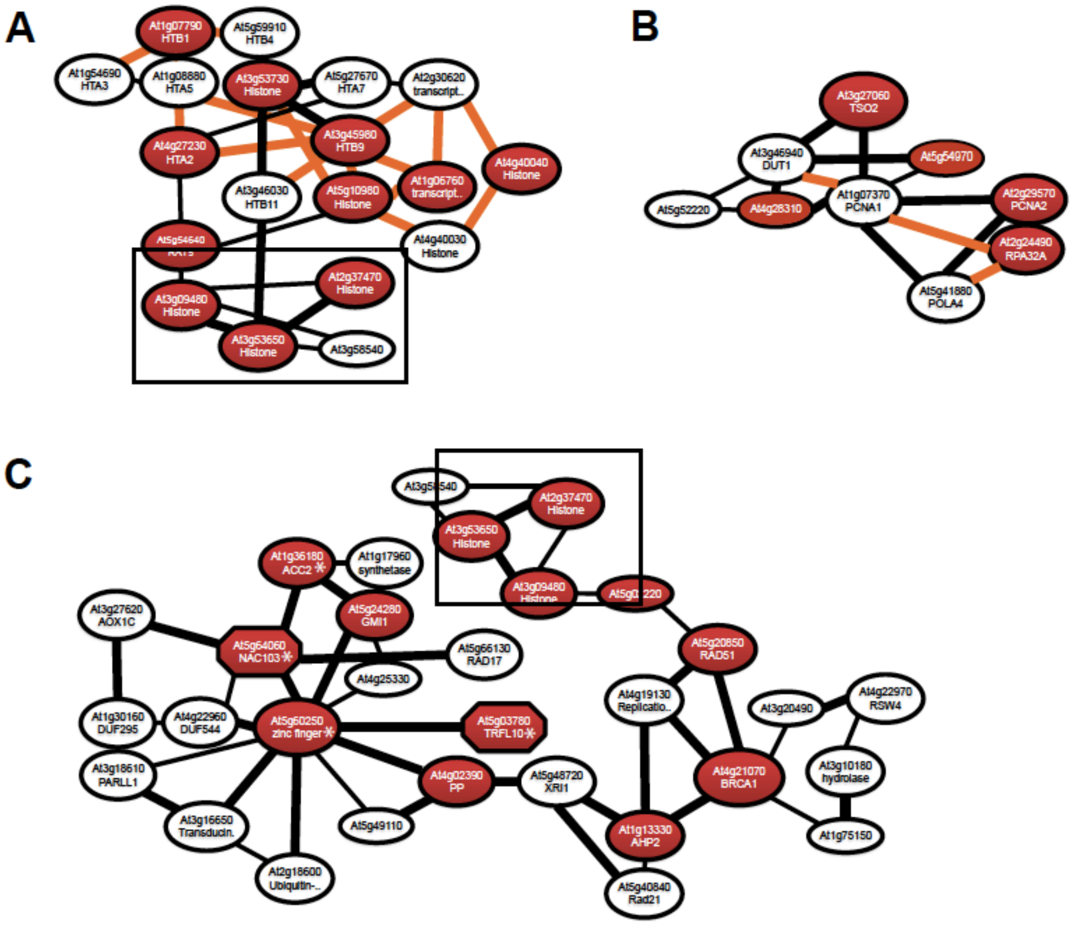

**Appendix Table S1 –Differentially expressed genes comparing transcriptome of *rRBr* and Col-0**																							
											sog1-	*atm-2*	*E2F*	*CYCD3*	*E2Fa*	inducible		RBR	RNAi				
													motif	*OE*	OE								
	Annotation	Locus	FC		adj. p-value											3hai	6hai	12ha	24hai	3hai	6hai	12hai	24hai
	**Upregulated**		(≥1.4)			ATEDII										up	up	up	up	down	down	down	down
1	Histone superfamily protein	At1g09200	1.62	11.74	1.53E-05		x						x	xxx	#	0	0	7	9	0	0	0	0
2	Acetyl-CoA carboxylase 2 (ACC2)	At1g36180	3.12	5.68	2.44E-05	C		x								0	0	0	0	0	0	0	0
3	Histone H2A protein (HTA13)	At3g20670	1.93	11.11	2.44E-05		x							xxx	#	0	0	8	9	0	0	0	0
4	Unknown protein	At5g05180	5.66	6.15	2.44E-05									xxx	#	0	0	0	5	0	0	0	0
5	Chromatin remodeling factor CHR31)	At1g05490	1.5	3.96	4.74E-05								x		#	0	0	0	0	0	0	0	0
6	Zinc finger (C3HC4-type RING finger) family protein	At5g60250	4.04	5.15	4.76E-05	C		x			x	xx	x	xxx	#	0	0	0	0	0	0	0	0
7	Histone superfamily protein	At3g53650	1.87	8.33	1.46E-04	AC	x	x					x	xxx	#	0	0	9	9	0	0	0	0
8	Histone2A protein (HTA1)	At5g54640	1.94	9.26	1.46E-04	A	x							xxx	#	0	0	6	0	0	0	0	0
9	Histone 2B protein (HTB9)	At3g45980	1.49	12.12	1.46E-04	A	x						x	xxx	#	0	0	2	9	0	0	0	0
10	Histone superfamily protein	At2g37470	1.48	9.25	1.48E-04	AC	x							xxx		0	0	5	9	0	0	0	0
11	Histone superfamily protein	At3g27360	1.63	10.57	1.84E-04		x							xxx		0	0	7	9	0	0	0	0
12	Histone superfamily protein	At1g07820	1.51	10.99	2.12E-04		x						x	xxx	#	0	0	2	6	0	0	0	0
13	Homolog of Drosophila timeless (ATIM)	At5g52910	1.56	6.85	2.35E-04				x				x	xxx	#	0	0	9	9	0	0	0	0
14	Unknown protein	At3g48490	1.99	7.24	2.44E-04								x	xxx	#	0	0	9	9	0	0	0	0
15	Siamese-related, SMR4	At5g02220	4.3	7.22	2.44E-04	C		x			x	xx	x	xxx	#	0	0	1	0	0	0	0	0
16	Histone H2A protein (HTA2)	At4g27230	1.74	10.46	2.64E-04	A	x							xxx	#	0	0	7	6	0	0	0	0
17	Siamese-related, SMR6	At5g40460	1.57	6.26	2.64E-04								x		#	0	0	0	0	0	0	0	0

18	Histone superfamily protein	At5g59690	1.55	13.04	3.48E-04		x						x	xxx	#	0	0	3	9	0	0	0	0
19	Histone H2A protein (HTA6)	At5g59870	1.4	12	3.48E-04		x						x	xxx	#	0	0	5	9	0	0	0	0
20	Histone H1.1 protein	At1g06760	1.52	11	4.91E-04	A	x									0	0	0	9	0	0	0	0
21	Homolog to breast cancer susceptibility gene 1 (BRCA1)	At4g21070	2.04	6.59	5.23E-04	C		x			x	xx	x	xxx	#	0	0	8	7	0	0	0	0
22	Histone 2B protein (HTB1)	At1g07790	1.56	10.73	5.85E-04	A	x									0	0	7	8	0	0	0	0
23	Histone superfamily protein	At5g10400	1.53	10.63	6.45E-04		x						x	xxx	#	0	0	7	9	0	0	0	0
24	Histone superfamily protein	At2g28720	2.71	7.63	6.68E-04		x									0	0	7	8	0	0	0	0
25	Histone H2A protein (HTA11)	At3g54560	1.56	10.72	6.86E-04		x						x	xxx	#	0	0	0	9	0	0	0	0
26	Kip-related protein (KRP3)	At5g48820	1.68	7.31	7.21E-04				x	x					#	0	0	0	3	0	0	0	0
27	Histone superfamily protein	At3g09480	1.75	8.54	7.85E-04	AC	x									0	0	8	7	0	0	0	0
28	Adenine nucleotide alpha hydrolases-like superfamily protein	At1g44760	1.68	7.87	8.39E-04											0	0	0	0	0	0	0	0
29	Histone superfamily protein	At5g10980	1.45	12.32	1.02E-03	A	x									0	0	0	1	0	0	0	0
30	Histone superfamily protein	At5g65360	1.39	11.29	1.02E-03		x					xx	x	xxx	#	0	0	4	9	0	0	0	0
31	Homolog of yeast RAD51	At5g20850	1.42	6.95	1.05E-03	C		x			x	xx			#	0	0	6	0	0	0	0	0
32	Histone superfamily protein	At5g02570	1.67	6.54	1.19E-03		x									0	0	3	0	0	0	0	0
33	High mobility group B (HMGB6)	At5g23420	1.58	8.08	1.55E-03				x				x	xxx	#	0	0	7	9	0	0	0	0
34	WRKY DNA-binding protein 21 (WRKY21)	At2g30590	1.54	7.47	1.61E-03										#	0	0	0	0	0	0	0	0

35	Minichromosome maintenance complex (MCM7), Prolifera	At4g02060	1.54	9.21	1.82E-03				x				x	xxx	#	0	0	9	9	0	0	0	0
36	Histone superfamily protein	At3g53730	1.41	11.73	1.91E-03	A	x						x	xxx	#	0	0	7	9	0	0	0	0
37	Histone superfamily protein	At4g40040	1.66	12.06	2.00E-03	A	x						x	xxx		0	0	0	0	0	0	0	0
38	DNA dependent nuclear poly (ADP-ribose) polymerase (PARP2)	At4g02390	1.68	6.68	2.09E-03	C		x			x	xx			#	0	0	2	1	0	0	0	1
39	Histone 3 11 (HTR11)	At5g65350	2.25	5.65	2.50E-03		x						x			0	0	0	0	0	0	0	0
40	Protein kinase superfamily protein	At4g35030	1.57	6.21	2.60E-03							xx	x			0	0	0	0	0	0	0	3
41	Unknown protein	At1g35780	1.49	9.18	2.60E-03								x	xxx		0	0	0	0	0	0	0	0
42	Unknown protein	At5g54970	1.49	10.26	2.71E-03	B			x				x	xxx	#	0	0	9	9	0	0	0	0
43	Catalytic subunit of the DNA polymerase alpha, putative (ICU2)	At5g67100	1.5	7.42	3.29E-03			x	x	x			x	xxx	#	0	0	0	9	0	0	0	0
44	Histone superfamily protein	At5g50930	1.44	5.97	3.47E-03									xxx	#	0	0	6	6	0	0	0	0
45	AT hook motif DNA-binding family protein (AHP1)	At2g33620	1.46	7.2	3.47E-03											0	0	0	0	0	0	0	0
46	Ribonucleotide reductase (RNR) small subunit gene (TSO2)	At3g27060	1.5	10.55	3.50E-03	B		x	x	x		xx	x	xxx	#	0	0	8	9	0	0	0	0
47	D-mannose binding lectin protein	At5g03700	1.4	7.47	3.50E-03								x		#	0	0	0	0	0	0	7	4
48	TESMIN/TSO1-like CXC 2 (TCX2)	At4g14770	1.99	7.72	3.50E-03				x				x	xxx	#	0	0	8	9	0	0	0	0
49	Structural Maintenance of Chromosomes 6A (SMC6A)	At5g07660	1.78	4.53	3.50E-03			x	x				x		#	0	0	7	0	0	0	0	0
50	Transducin/WD40 repeat-like superfamily protein	At3g27640	1.37	7.21	3.50E-03				x				x		#	0	0	7	7	0	0	0	0

51	Member of TRFL family 2 (TRFL10)	At5g03780	1.44	5.67	3.58E-03	C		x			x	xx			#	0	0	0	0	0	0	0	0
52	Glycoside Hydrolase Family 16 (XTH28)	At1g14720	1.61	7.01	4.05E-03							xx	x			0	0	0	0	0	0	0	0
53	Actin-binding formin homology 2	At3g07540	1.73	5.51	4.08E-03											0	0	0	0	0	0	8	4
54	Histone superfamily protein	At3g45930	1.49	10.9	4.09E-03		x						x	xxx	#	0	0	9	9	0	0	0	0
55	Proliferating Cell Nuclear Antigen 2 (PCNA2)	At2g29570	1.41	10.81	4.43E-03	B			x				x	xxx	#	0	0	9	9	0	0	0	0
56	Histone 2B protein (HTB2)	At5g22880	1.66	9.33	4.43E-03		x						x	xxx	#	0	0	6	9	0	0	0	0
57	ATP binding microtubule motor family protein	At3g63480	1.6	6.39	4.61E-03				x						#	0	0	7	0	0	0	0	0
58	Histone 4	At2g28740	1.39	9.88	4.61E-03		x						x		#	0	0	6	8	0	0	0	0
59	Cytochrome P450 superfamily protein	At1g73340	1.88	7.63	4.77E-03											0	0	0	0	0	0	0	0
60	NAC domain containing protein 103 (NAC103)	At5g64060	1.72	5.36	5.02E-03	C		x			x	xx		xxx	#	0	0	0	0	0	0	0	0
61	Unknown protein	At4g28310	1.41	9.32	5.34E-03	B			x				x	xxx	#	0	0	9	9	0	0	0	0
62	Agenet domain-containing protein	At1g26540	1.65	4.85	5.68E-03								x		#	0	0	5	3	0	0	0	0
63	Histone superfamily protein	At5g10390	1.43	9.96	5.78E-03		x						x	xxx	#	0	0	7	9	0	0	0	0
64	Cytokinin response factor (CRF6)	At3g61630	1.52	4.59	5.78E-03							xx	x			0	0	3	0	0	0	4	7
65	DNA polymerase alpha 2 (POLA2)	At1g67630	1.55	7.69	5.78E-03			x	x	x				xxx	#	0	0	9	9	0	0	0	0
66	Chromatin remodeling factor17 (CHR17)	At5g18620	1.54	9.4	5.78E-03				x				x	xxx	#	0	0	5	9	0	0	0	0
67	Cystatin/monellin family protein	At5g05110	1.76	6.25	5.78E-03									xxx		0	0	0	0	0	0	0	0
68	GATA transcription factor (GATA5)	At5g66320	1.53	6.22	5.78E-03											0	0	5	9	0	0	0	0

69	Homolog of homologous -pairing protein2 hop2 (AHP2)	At1g13330	1.97	4.94	6.18E-03	C		x	x			xx		xxx	#	0	0	6	0	0	0	0	0
70	NAC domain protein (BRN2)	At4g10350	2.52	7.65	6.27E-03								x			0	0	0	0	0	0	0	0
71	Homolog of the human centromeric protein C (CENP-C)	At1g15660	1.48	8.87	6.58E-03				x	x			x	xxx	#	0	0	1	7	0	0	0	0
72	Protein of unknown function (DUF239)	At1g70550	1.68	7.4	6.72E-03									xxx		0	0	0	0	2	0	0	0
73	Gamma-irradiation and mitomycinin C induced 1 (GMI1)	At5g24280	2.09	5.28	7.10E-03	C		x			x	xx		xxx	#	0	0	3	0	0	0	0	0
74	Unknown protein	At3g01860	1.42	5.23	7.25E-03								x			0	0	0	0	0	0	0	0
75	Senescence/dehydration-associated protein-related	At4g35985	1.47	6.5	7.25E-03								x			0	0	1	0	0	0	1	0
76	High mobility group A (HMGA)	At1g14900	1.5	9.43	7.53E-03		x						x	xxx		0	0	3	0	0	0	0	0
77	F-box family protein	At4g35930	1.49	6.42	8.55E-03								x		#	0	0	0	3	0	0	0	0
78	TRAM, LAG1 and CLN8 (TLC) lipid-sensing domain containing protein	At1g21790	1.5	6.68	8.56E-03											0	0	0	0	0	0	0	4
79	WRKY transcription factor (WRKY48)	At5g49520	1.76	4.66	8.56E-03								x		#	0	0	0	0	0	0	8	6
80	Replicon Protein A2 (RPA2)	At2g24490	1.45	9.24	8.63E-03	B			x	x			x	xxx	#	0	0	9	9	0	0	0	0
81	DNA primase, large subunit family	At1g67320	1.43	7.96	9.31E-03				x	x			x	xxx	#	0	0	6	9	0	0	0	0
82	Kip-related protein (KRP5)	At3g24810	1.6	6	9.31E-03					x						0	0	0	6	0	0	0	0
														46					58			7	
	**Down-regulated**																						
1	Phosphatidylinositol 3- and 4-kinase	At5g24240	-4.31	5.89	2.57E-06								x	xxx		0	0	0	0	0	0	0	0

2	HSP20-like chaperones superfamily protein	At5g47600	-3.93	6.15	3.58E-03											0	0	0	0	0	0	0	0
3	ARF GTPase family (ARFB1B)	At5g17060	-3.71	7.06	2.36E-08											0	0	0	0	0	0	0	0
4	Unknown protein	At5g15725	-3.65	5.21	1.46E-04											0	0	0	0	0	0	0	0
5	TRICHOME BIREFRINGENCE-LIKE (TBL6)	At3g62390	-3.09	6.34	1.53E-05											0	0	0	0	0	0	0	6
6	Retinoblastoma-related protein (RBR)	At3g12280	-2.26	7.86	7.85E-06				x	x			x	xxx	#	0	0	0	0	0	4	9	7
7	emp24/gp25L/p24 family/GOLD family protein	At3g10780	-2.22	5	6.92E-03								x			0	0	0	0	0	0	0	0
8	QUA-QUINE STARCH (QQS)	At3g30720	-1.92	4.77	2.64E-04											0	4	0	1	0	0	3	0
9	Putative receptor serine/threonine kinase PR5K (PR5K)	At5g38280	-1.8	5.49	1.37E-03											0	0	0	0	0	0	0	0
10	D-type cyclin CYCD4 (CYCD4;1)	At5g65420	-1.69	5.63	4.61E-03					x						0	0	1	0	0	0	0	0
11	WNK protein kinases (WNK7)	At1g49160	-1.61	5.17	5.78E-03											0	0	0	0	0	0	0	0
12	MRP subfamily (MRP11)	At2g07680	-1.51	6.29	1.19E-03											0	0	0	0	0	0	0	0
13	BRGs (BOI-related gene) involved in resistance to Botrytis cinerea (BRG2)	At1g79110	-1.48	4.21	6.94E-03								x		#	0	0	2	8	0	0	0	0
14	D-type cyclin CYCD4 (CYCD4;2)	At5g10440	-1.48	4.63	8.39E-04					x						0	0	0	0	0	0	0	0
15	Oxidoreductase activity, acting on the CH-CH group of donors	At1g18180	-1.46	7.08	3.47E-03											0	0	0	0	0	0	1	0
16	RNA-binding KH domain-containing protein	At3g32940	-1.46	6.85	3.58E-03											0	0	0	0	0	0	0	0

17	Purple acid phosphatase 27 (PAP27)	At5g50400	-1.45	7.35	4.77E-03											0	0	0	0	0	0	0	0
							32	14	19	6	8	14	53	48	56			2				3	

Appendix Table S2

Enrichment of Gene Ontology (GO) terms of differentially expressed genes

	GO term	GO ID
Up-regulated		
	Nucleosome assembly	GO:0006334
	Chromatin organization	GO:0006325
	Chromosome organization	GO:0051276
	Nucleosome	GO:0000786
	DNA binding	GO:0003677
	Cellular component organization at cellular level	GO:0071842
	Nucleus	GO:0005634
	DNA metabolic process	GO:0006259
	DNA repair	GO:0006281
	Response to DNA damage stimulus	GO:0006974
	Response to ionizing radiation	GO:0010212
	Cellular process	GO:0009987
	DNA-dependent ATPase activity	GO:0008094
	Cyclin-dependent protein kinase inhibitor activity	GO:0004861
Down-regulated		
	Regulation of cell cycle	

Appendix Table S3
Primers used in this study

Gene / primer name	Accession no. / sequence
**A. Chromatin immunoprecipitation**	
**At4g21070**	**AtBRCA1**
BRCA1F	gtttcatcgcagatcgttca
BRCA1R	ttgcaagatttgaacccatt
BRCA2F	taggggcaaaacgaaaattg
BRCA2R	agatgacgaagcgtgtcctt
BRCA3F	ttctcttgatttcagtgcgtgt
BRCA3R	aatcatcaaactgcaaacttagg
**At1g07370**	**PCNA1**
PCNA4F	aatgaccaaaaatatccatccaa
PCNA4R	cggctattttggaaagtgga
**IR**	**At3g03660-70**
IR1F	cattgaacacctattggtaggaa
IR1R	actgttttggtgccagattttcag
**B. qRT-PCR**	
**At3g18780**	**ACTIN2**
qActinF	CGCTGACCGTATGAGCAAAG
qActinR	TTCATGCTGCTTGGTGCAA
**At3g12280**	**RETINOBLASTOMA-RELATED**
qRBRF2	ATAATAAGCCTGAAGGTCAATGTC
qRBRR2	TAAACATTGTGCACTGCAGATACT
**At2g21070**	**BREAST CANCER ASSOCIATED-1**
qBRCA1F2	TCATGGGAGATTTCGAGCTT
qBRCA1R2	ATTTAGCCAAGGCTTCAGCA
qBRCA330F	TGGAAGATGCTTCTGGGATT
qBRCA480R	CTCGTTCCTCTTGGATGCTC
qBRCA2210F	TGCACTCTCAGCCTAAACAAG
qBRCA2380R	ACTCCAGACAGTTCCGCAAA
**At4g02390**	**POLY-(ADP-RIBOSE) POLYMERASE2**
qPARP2F	TCGAGAGCTGTTGAAGCTGA
qPARP2R	GGAGCTATTCGCAGACCTTG
**At1g09200**	**HISTONE3.1**
qH3.1F	AGCAGACGGCTAGGAAATCA
qH3.1R	CAACAGTTCCGGGTCTGAAT
**At5g02220**	**SMR4, SIAMESE-RELATED 4**
At5g02220F	GGCGTCTGTTTGTCCACC
At5g02220R	CCCTAAACATGTATCTACAGAGAAG
**At1g07500**	**SMR5, SIAMESE-RELATED 5**
At1g07500F	AAACTACGACGACGGAGATACG
At1g07500R	GCTACCACCGAGAAGAACAAGT
**At5g24280**	**GMI, GAMMA-IRRADIATION AND MITOMYCIN C INDUCED**
qGMIF	CCTCATTGTTGGATCCTTGG
qGMIR	TCCCAGCATAAGCTCCTCTC
**At5g20850**	**RAD51**
qRAD51F	TTGCTGGTCCCCAATTTAAG
qRAD51R	CAAACATGGCGAGCTTATCA
**At5g10440**	**CYCD4.2**
qCYCD4.2F	GCAGTGACACCGTGCTCTTA
qCYCD4.2R	ACTGCAGCAGCAATCTCTGA

**At5g24240**	**PHOSPHOTIDYLINOSITOL 3 and 4-KINASE**
qPIPF	GCACTATGGCTTCTGCATCA
qPIPR	GCACTATGGCTTCTGCATCA

C. Genotyping

brca1-1	**At4g21070**
BRCA1-1F(LP)	agagtcgctttgttcctgattc
BRCA1-1R(RP)	gatgctcggcccttccta
LBb1.3	attttgccgatttcggaac
brca1-1/brca1-3	
brca1koF	tggagaggatgggaagagaa
brca1koR	cactgcctttgtttcgttca
e2fb-2	**At5g22220**
E2FB-RP	gtgcctttacagctatcagcg
E2FB-LP	ttggattccttccatttgatg
LBb1.3	attttgccgatttcggaac

**Col-0*(CYCD3OE)***	
Forward(CaMV35S)	TCCGGAAACCTCCTCGGA (CaMV35S)
Reverse	GCTGCGGCAACTACTGATGG
CYCD3;1 FLANKING	
Forward	TAAACTCAGCCGTCCGATCAC
Reverse	TGAGATTCATGGTGATAACCTCG

D. Cloning:

pBRCAF1	gtttcatcgcagatcgttca
pBRCAR2	tttcgatcttcactcagag
gBRCA1F1	ATGGCGGACACTAGTCACC
gBRCA1R1	TATCAAGACTAAAATTTGGC

BiFC cDNA	
BRCA1cDNAF	ggggacaagtttgtacaaaaaagcaggctgta* (vector)
	*TGGCGGACACTAGTCACCTGGAGAGGATGGGAAGAG
BRCA1cDNAR	ggggaccactttgtacaagaaagctgggtt*(vector)
	*TATCAAGACTAAAATTTGGCAACCTGCAATAGAAT**
	**CCAAAACCCATCCAAAACCC

Appendix Table S4

Label free MS quantitation of E2FA interaction with RBR, DPA and DPB -/+ MMC

Identified DREAM-complex proteins		Number of unique/total peptide identifications			
Name	AGI number	E2FA untreated		E2FA treated (MMC)	
		unique	total	unique	total
E2FA	AT2G36010	12	28	9	23
RBR1	AT3G12280	31	44	32	52
DPB	AT5G03415	8	18	6	13
DPA	AT5G02470	7	11	6	9

Appendix Table S5

Interaction of E2FA or E2FB with DREAM complex components

Identified DREAM-complex proteins		UNIQUE PEPTIDES/ % COVERAGE	
Name	AGI number	E2FA*	E2FB*
E2FA	AT2G36010	12/28.2%	-
E2FB	AT5G22220	-	17/44.3%
RBR1	AT3G12280	28/31.8%	37/37.2%
DPB	AT5G03415	9/30.9	7/25.2%
DPA	AT5G02470	6/19.2%	5/18.2%
ALY3	AT3G21430	-	2/4.3%
TCX5	AT4G29000	-	3/3%
MSI1	AT5G58230	-	3/8.7%

*Representative result from independent experiments (n>10)
