# Supplementary material for: Arabidopsis RETINOBLASTOMA RELATED directly regulates DNA damage responses through functions beyond cell cycle control
Source: EMBO J. 2017 Mar 20;36(9):1261–78. doi: 10.15252/embj.201694561 (PMC5412863; doi:10.15252/embj.201694561)
Supplement: Supplementary file 2 — Expanded View Figures PDF [file EMBJ-36-1261-s002.pdf]

Expanded View Figures

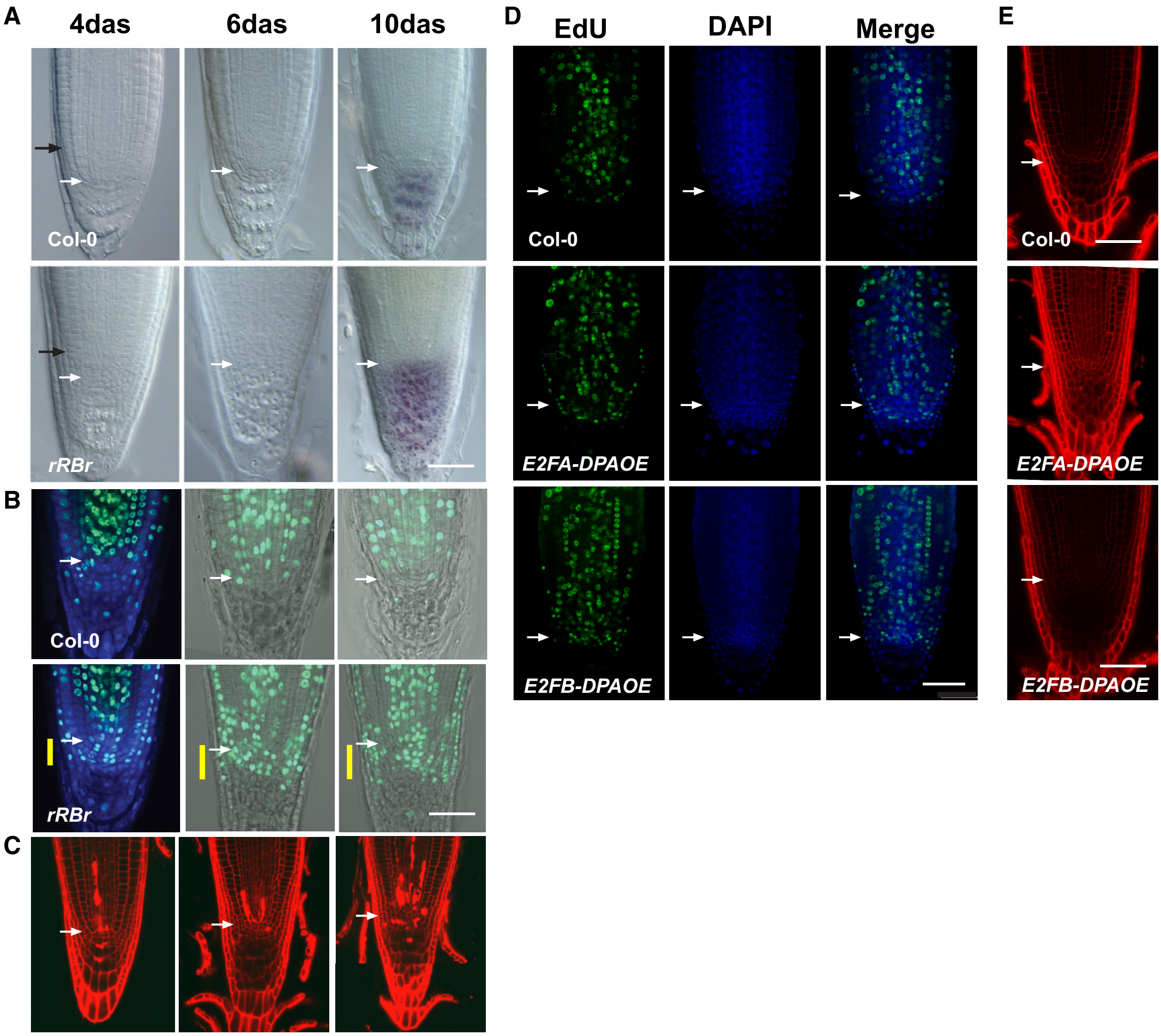

Figure EV1. Both local silencing of RBR and overexpression of E2FA-DPA and E2FB-DPA result in extra S-phase entry but only RBR silencing triggers cell death response.

- A Differential interference contrast (DIC) microscopy images using Lugol staining to detect differentiated columella cells. Note the increased number of columella cell layers upon local reduction of RBR. Black arrow indicates the position of the dissection used to collect material for micro-array analysis.
- B Confocal microscopy images (CM) of root tips after EdU staining (green, 2 h) at 4 das counterstained with DAPI (blue) and at 6 and 10 das using bright field. Yellow bar indicates the region with extra columella stem cell layers.
- C CM images of PI-stained root samples from *rRBr* seedlings showing accumulation of cell death in time.
- D Representative CM images of whole mount EdU-labelled (green) root tips of 6 das Col-0, Col-0(*E2FA/DPAOE*) and Col-0(*E2FB/DPAOE*) seedlings; DNA was stained by DAPI.
- E Representative PI-stained CM images of 12 das root tips from the genotypes indicated. Note that no cell death response was detected at any time point analysed.

Data information: Images were taken in median sections. Scale bars: 50 μm, genotype as indicated in the images. White arrows: QC position in each image.

© 2017 The Authors

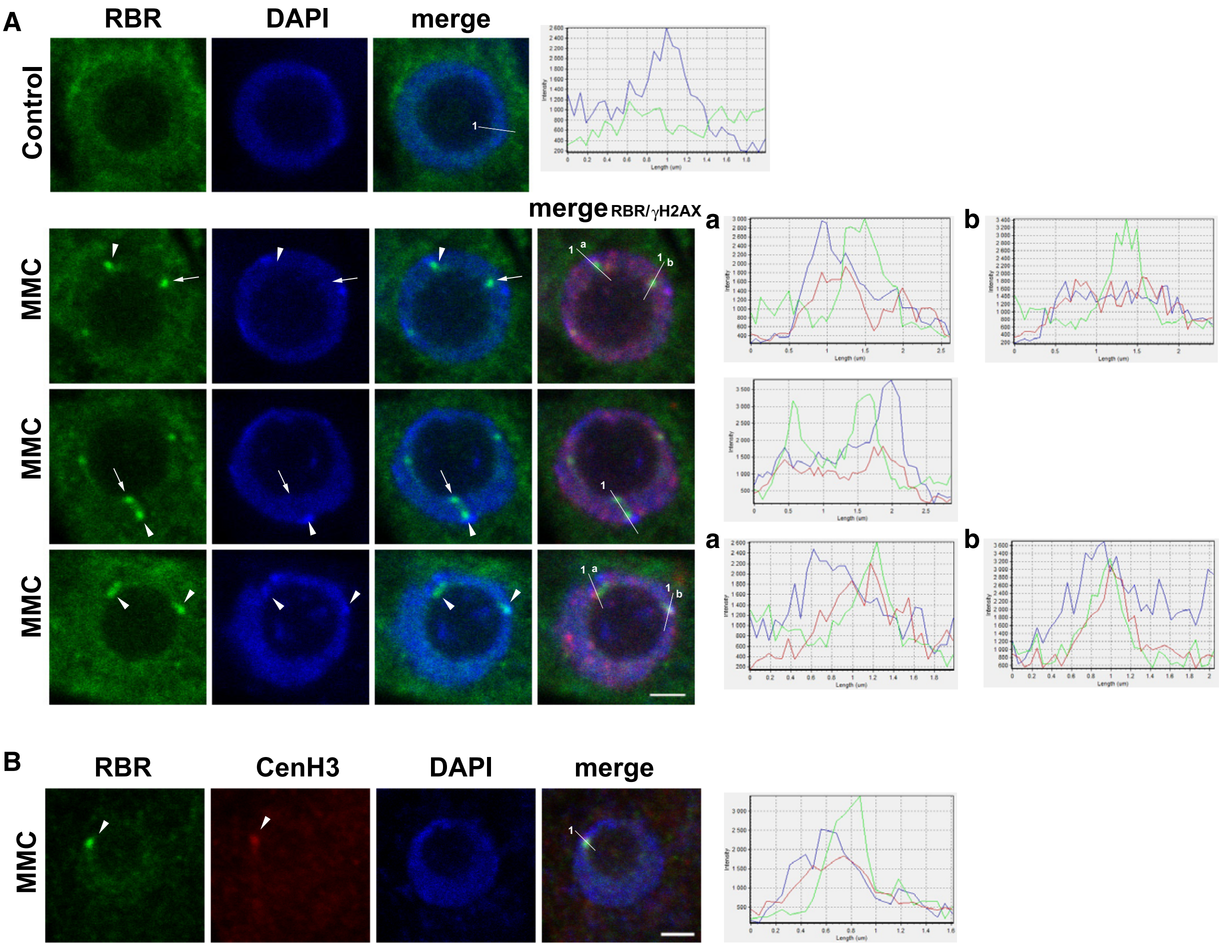

Figure EV2. RBR nuclear foci can localise with condensed heterochromatin and CenH3.

A Representative CM images of nuclei (single section) of Col-0 6 das root tips after 16 h of MMC treatment immunolabelled for RBR (green), γH2AX (red) and DAPI (blue). RBR foci localised at γH2AX-positive sites and with DNA heterochromatin spots labelled by arrowheads, while RBR foci localised independently of condensed chromatin are marked by arrows and (a) and (b) illustrate intensity profiles for a section as given in merged images.

B Representative CM image of nuclei (single section) showing localisation of RBR foci to CenH3-labelled region (arrowheads) in 6 das Col-0 root tips after 16 h of MMC treatment (RBR: green, CenH3: red, DAPI: blue).

Data information: In (A and B) intensity profiles: *x*-axis shows length in μm measured from 1; *y*-axis shows relative intensity. Scale bars: 2 μm. $N > 3$, $n = 3$. n = biological repeats, N = samples per biological repeat.

 © 2017 The Authors

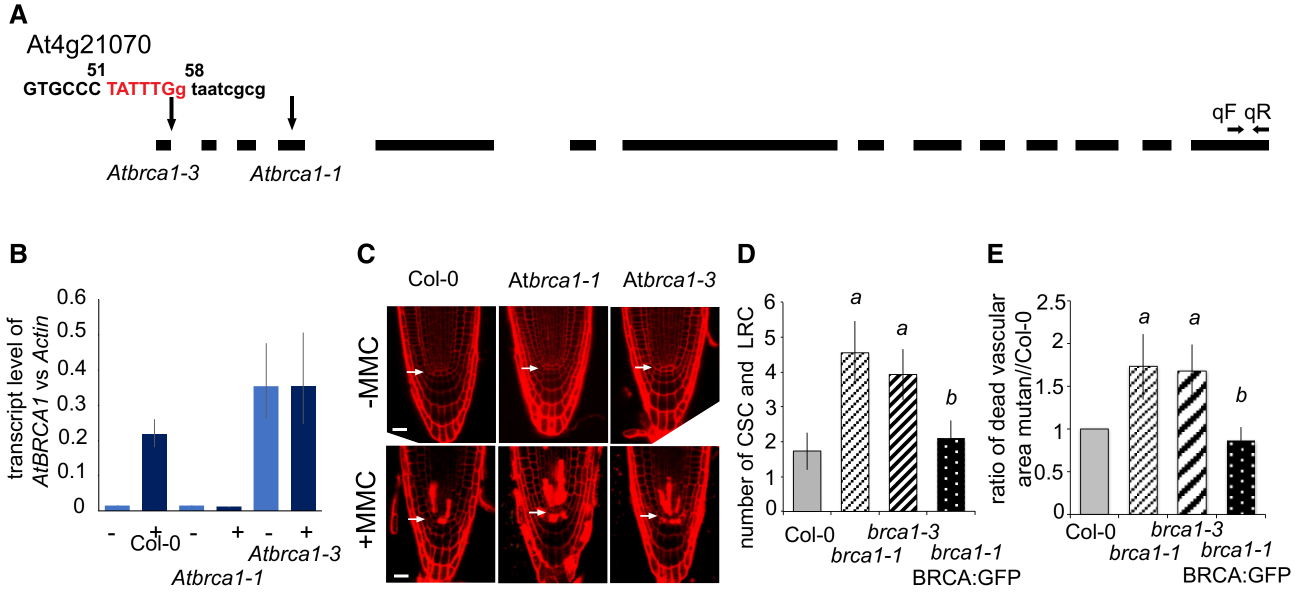

Figure EV3. The *Atbrca1-3* mutant, similarly to *Atbrca1-1*, also shows hypersensitivity upon genotoxic stress.

A Position of the T-DNA insertion in *Atbrca1-1* (Reidt *et al*, 2006) and *Atbrca1-3* mutants. The sequence indicates the insertion in *Atbrca1-3*, and difference in letter type shows the exon–intron border. Arrows depict position of forward (qF) and reverse (qR) primers used for qRT–PCR reactions.

B Expression level of the *AtBRCA1* transcript in Col-0, *Atbrca1-1* and *Atbrca1-3* alleles compared to the *AtACTIN2* transcript level in Col-0 in normal growth conditions. To control the inducibility of the transgenes, the alleles and Col-0 were treated with MMC (+) and compared to non-treated seedlings (−). The graph shows that genotoxic stress influenced the *AtBRCA1* transcript level only in the control, but not in the alleles. However, neither of the *Atbrca1* alleles are null alleles. $n > 2$, $N > 100$ seedlings (6 das) for each genotype and treatments.

C CM images of PI-stained root tips of Col-0, *Atbrca1-1* and *Atbrca1-3* 6 das seedlings grown without (−MMC) and treated with MMC (+MMC) for 16 h. Scale bar: 20 µm, arrow: QC position in each image.

D, E Functional analysis of the ($AtBRCA1_{pro}$:$AtBRCA1_{gen}$:*GFP*) construct following cell death response in the introgressed line *Atbrca1-1*(pgBRCA:GFP) compared to Col-0, *Atbrca1-1* and *Atbrca1-3*. (D) Cell death response was quantified in the distal stem cell region after 16 h of MMC treatment. Dead columella stem and daughter cells (CSC) and lateral root initials and their descendants (LRC) were counted in median section as shown in (C); $n = 3$, $N > 15$. (E) Ratio of PI-stained area in the proximal meristem comparing mutants and the complementing line to Col-0. PI-stained area was measured in each experiment from $N > 15$ mutants and Col-0, and then means and ratio were calculated. Finally, the mean of the different experiments/ratios ($n = 3$–4) was calculated and depicted.

Data Information: In (B, D and E), values represent means ± standard deviation. *a* indicates significant difference around 1% confidence using Student's *t*-test comparing *Atbrca1-1* and *Atbrca1-3* to Col-0, and *b* indicates 99% significance between *Atbrca1-1*(AtBRCA1:GFP) and *Atbrca1-1*. *n* = biological repeats, *N* = samples per biological repeat.

© 2017 The Authors

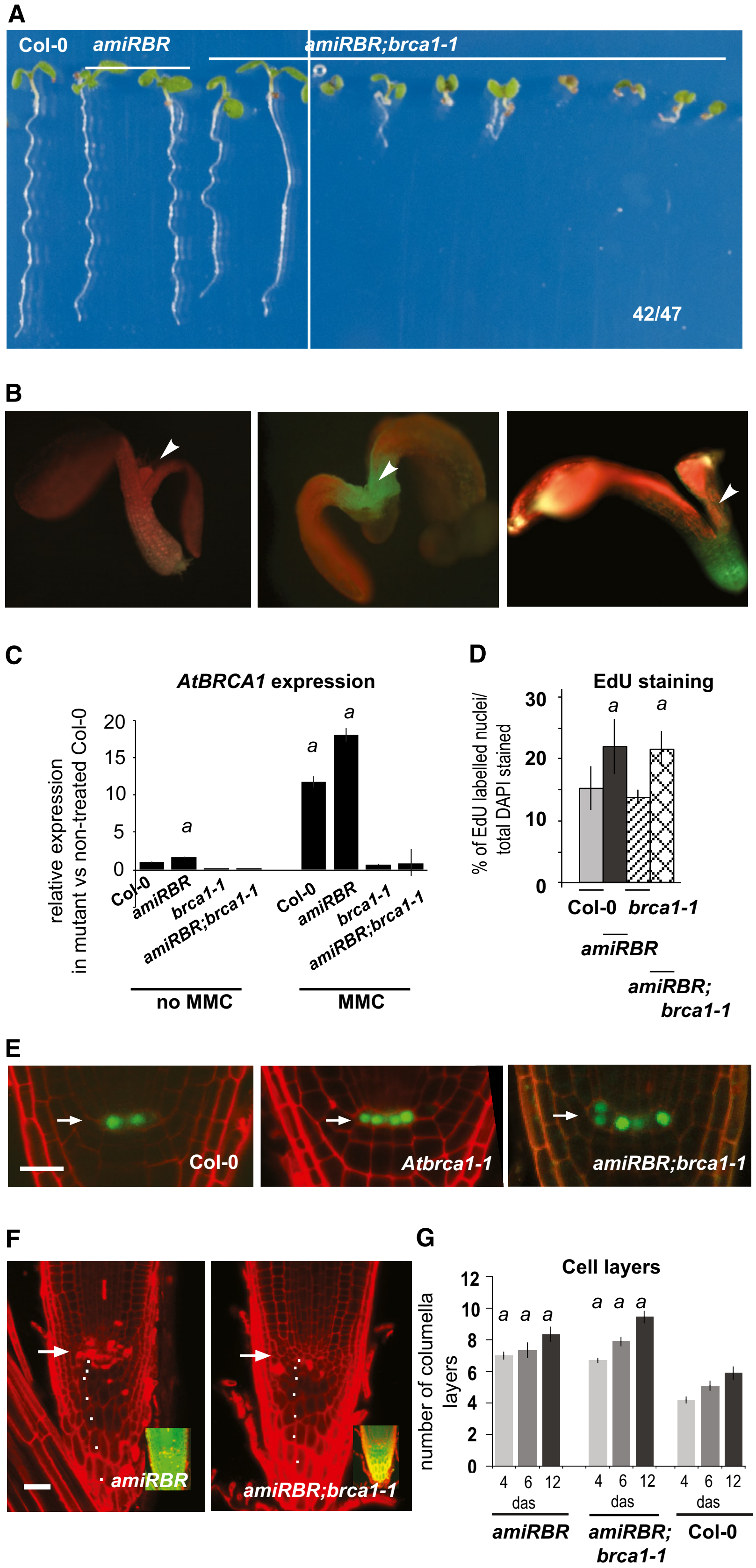

Figure EV4. Lack of AtBRCA1 in conjunction with RBR silencing results in partially penetrant developmental arrest and suppresses cell death response, but does not influence extra stem cell division and S-phase entry induced by RBR silencing in surviving individuals.

A Segregation (47 severe/49 survival) and growth habit of *amiRBR;Atbrca1-1* homozygous seedlings (F3).

B Developmental defects in germinating seedlings, arrowheads point to missing primary leaves.

C Relative transcript level of *AtBRCA1* in *amiRBR*, *Atbrca1-1* and *amiRBR;brca1-1* compared to Col-0, where the level of expression was set arbitrarily to 1 in non-treated samples. Upon MMC treatment, the graph shows the ratio of expression between treated and non-treated samples. Values represent mean ± SD, $n > 2$, $N > 100$. *a*: $P < 0.05$ shows significant increase in expression compared to Col-0 untreated control using Student's *t*-test.

D Frequency (%) of EdU-labelled nuclei (10-min pulse) compared to total DAPI-stained nuclei, *a*: $P < 0.001$, all compared to Col-0 using Student's *t*-test, $n > 2$, $N > 10$; error bars indicate ± SD.

E $WOX5_{pro}$-$WOX5_{gen}$-3xGFP expression in the mutant lines showing QC division in the *amiRBR; brca1-1* compared to Col-0 and *Atbrca1-1*. Arrow indicates the position of the QC, scale bar: 20 µm.

F Confocal images of *amiRBR* and *amiRBR;brca1-1* root tips of 12 das seedlings showing columella and stem cell layers (white dots). Arrow: QC position. Inset is showing the incorporation and presence of the *amiRBR* construct. Scale bar: 20 µm.

G Quantification of the number of columella and stem cell layers of 4-, 6- and 12-day-old roots from *amiRBR*, *amiRBR;brca1-1* and Col-0. Values represent means ± SD, $N > 15$ for each mutant and Col-0 (n = 3–4). *a*: $P < 0.01$ between the given genotype and Col-0 at a given time point using Student's *t*-test.

Data information: n = biological repeats, N = samples per biological repeat.

 © 2017 The Authors

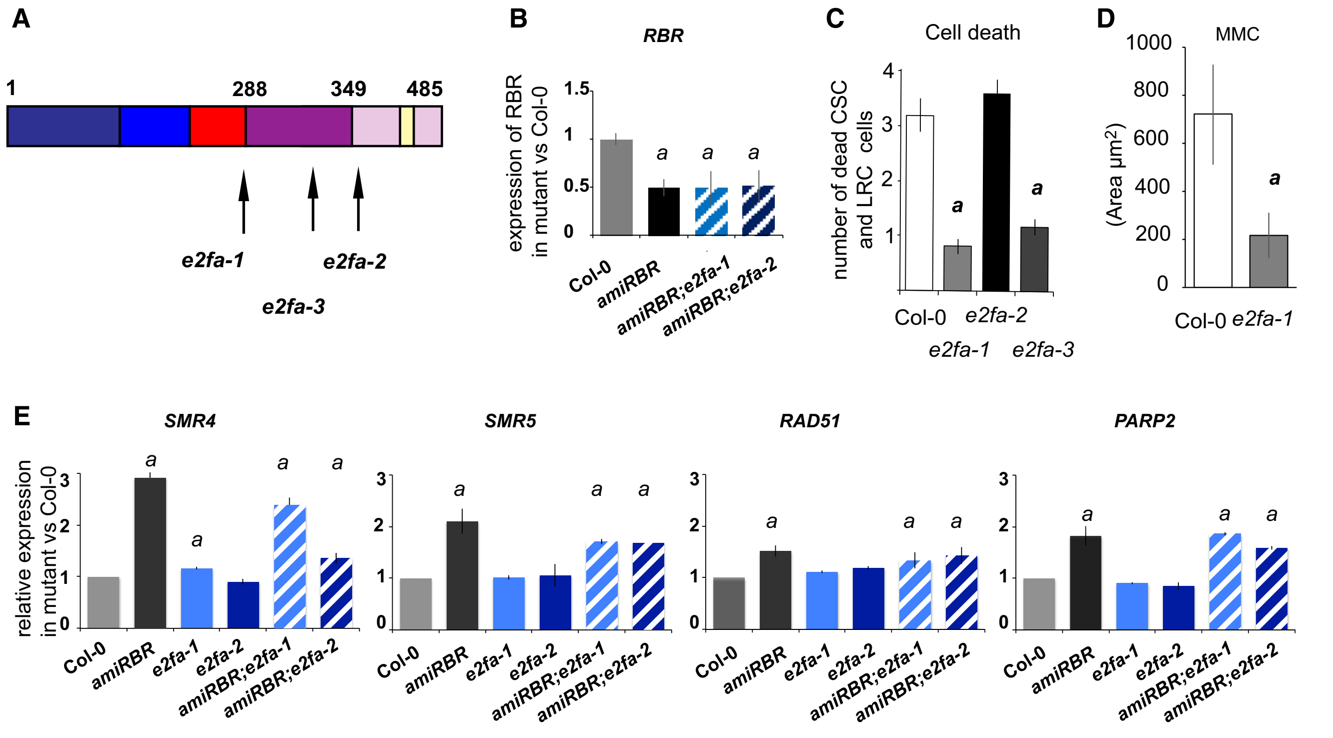

Figure EV5. Both transcription of *AtBRCA1* and *SMR4* upon RBR silencing and cell death response upon genotoxic stress are dependent of E2FA.

A Position of different T-DNA insertions in AtE2FA, colours represent different domains: dark blue, N-terminal; light blue, DNA-binding domain; red, dimerisation domain; purple, marked box; lilac, transactivation domain; yellow, RBR binding domain. Drawing based on Magyar *et al* (2012).

B Relative transcript level of RBR in *amiRBR* and *amiRBR;e2fa-1* and *amiRBR;e2fa-2* double mutants.

C Cell death response in MMC-treated 6 das seedlings of different *e2fa* alleles; total number of dead columella stem cells (CSC), lateral root cap initials (LRC) and their descendants were counted.

D Quantification of cell death by measuring the area of dead vasculature (μm^2) in the presence of MMC for 16 h. No cell death response was observed in non-treated samples.

E Relative transcript level of *SMR4*, *SMR5*, *RAD51* and *PARP2* in *amiRBR*, *e2fa-1*, *e2fa-2* and double mutants compared to Col-0, where the level of expression was set arbitrarily to 1. *a*: $P < 0.05$ significance between mutant versus Col-0 using Student's *t*-test; values represent mean of relative expression.

Data information: In (B–E), bars represent mean ± SD, $n > 2$, $N > 10$ seedlings in (C and D) and $N > 100$ in (B and E). *a*: $P < 0.05$ significance between different *e2fa* mutants versus Col-0 using Student's *t*-test. n = biological repeats, N = samples per biological repeat.

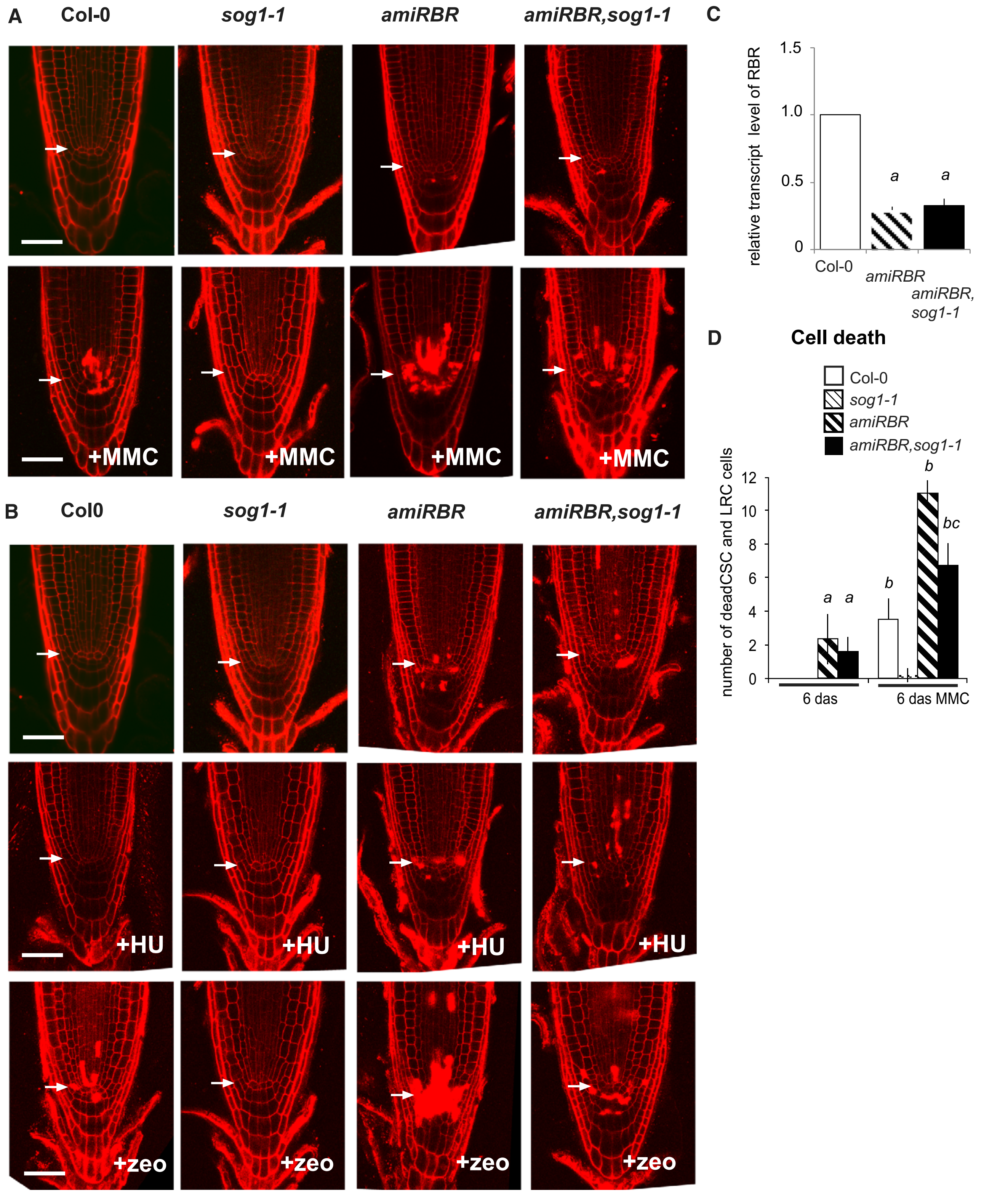

Figure EV6.

 © 2017 The Authors

◀ **Figure EV6. Hypersensitive cell death response after genotoxic stress only partially depends on SOG1 upon RBR silencing.**

A CM images of PI-stained root tips from *sog1-1*, *amiRBR* and *amiRBR,sog1-1* lines compared to Col-0 as indicated above the columns. Images were taken in median section of 6 das seedlings treated with and without MMC (10 μg/ml). Scale bar: 50 μm, arrow: QC position in each image.

B Representative images of 9 das seedlings showing cell death response after hydroxyurea (+HU, 1 mM) or zeocin (+zeo, 20 μg/ml) treatment. Scale bar: 50 μm, arrow: QC position in each image.

C Relative *RBR* transcript level in *amiRBR* and *amiRBR,sog1-1* lines, taking mean of several independent lines. *a*: $P < 0.05$ significance genotypes versus Col-0 using Student's *t*-test.

D Cell death response upon MMC treatment in *sog1-1*, *amiRBR* and *amiRBR,sog1-1* lines compared to Col-0. The total number of dead columella stem and daughter cells (CSC), lateral root cap initials (LRC) and their descendants were counted in median section as shown in (A). *a*: $P < 0.05$ significance genotypes versus Col-0, *b*: $P < 0.05$ comparison of treated samples to non-treated counterparts, *c*: $P < 0.05$ significance between *amiRBR* versus *amiRBR,sog1-1* using Student's *t*-test. Note, that Col-0 at 6 das and *sog1-1* at 6 and 12 das developed no dead cells.

Data information: Values represent mean ± SD, $n = 3$, $N > 15$ in (A, B and D) and $N > 100$ in (C) for each mutant and Col-0. n = biological repeats, N = samples per biological repeat.

© 2017 The Authors
